# Oncogenic YAP mediates changes in chromatin accessibility and activity that drive cell cycle gene expression and cell migration

**DOI:** 10.1093/nar/gkad107

**Published:** 2023-03-02

**Authors:** Maria Camila Fetiva, Franziska Liss, Dörthe Gertzmann, Julius Thomas, Benedikt Gantert, Magdalena Vogl, Nataliia Sira, Grit Weinstock, Susanne Kneitz, Carsten P Ade, Stefan Gaubatz

**Affiliations:** Theodor Boveri Institute and Comprehensive Cancer Center Mainfranken, Biocenter University of Wuerzburg, Wuerzburg 97074, Germany; Theodor Boveri Institute and Comprehensive Cancer Center Mainfranken, Biocenter University of Wuerzburg, Wuerzburg 97074, Germany; Theodor Boveri Institute and Comprehensive Cancer Center Mainfranken, Biocenter University of Wuerzburg, Wuerzburg 97074, Germany; Theodor Boveri Institute and Comprehensive Cancer Center Mainfranken, Biocenter University of Wuerzburg, Wuerzburg 97074, Germany; Theodor Boveri Institute and Comprehensive Cancer Center Mainfranken, Biocenter University of Wuerzburg, Wuerzburg 97074, Germany; Theodor Boveri Institute and Comprehensive Cancer Center Mainfranken, Biocenter University of Wuerzburg, Wuerzburg 97074, Germany; Theodor Boveri Institute and Comprehensive Cancer Center Mainfranken, Biocenter University of Wuerzburg, Wuerzburg 97074, Germany; Theodor Boveri Institute and Comprehensive Cancer Center Mainfranken, Biocenter University of Wuerzburg, Wuerzburg 97074, Germany; Theodor Boveri Institute and Comprehensive Cancer Center Mainfranken, Biocenter University of Wuerzburg, Wuerzburg 97074, Germany; Theodor Boveri Institute and Comprehensive Cancer Center Mainfranken, Biocenter University of Wuerzburg, Wuerzburg 97074, Germany; Theodor Boveri Institute and Comprehensive Cancer Center Mainfranken, Biocenter University of Wuerzburg, Wuerzburg 97074, Germany

## Abstract

YAP, the key protein effector of the Hippo pathway, is a transcriptional co-activator that controls the expression of cell cycle genes, promotes cell growth and proliferation and regulates organ size. YAP modulates gene transcription by binding to distal enhancers, but the mechanisms of gene regulation by YAP-bound enhancers remain poorly understood. Here we show that constitutive active YAP5SA leads to widespread changes in chromatin accessibility in untransformed MCF10A cells. Newly accessible regions include YAP-bound enhancers that mediate activation of cycle genes regulated by the Myb-MuvB (MMB) complex. By CRISPR-interference we identify a role for YAP-bound enhancers in phosphorylation of Pol II at Ser5 at MMB-regulated promoters, extending previously published studies that suggested YAP primarily regulates the pause-release step and transcriptional elongation. YAP5SA also leads to less accessible ‘closed’ chromatin regions, which are not directly YAP-bound but which contain binding motifs for the p53 family of transcription factors. Diminished accessibility at these regions is, at least in part, a consequence of reduced expression and chromatin-binding of the p53 family member ΔNp63 resulting in downregulation of ΔNp63-target genes and promoting YAP-mediated cell migration. In summary, our studies uncover changes in chromatin accessibility and activity that contribute to the oncogenic activities of YAP.

## INTRODUCTION

The evolutionary conserved Hippo pathway plays important roles in development, cell proliferation, organ size control and cancer (1,2–4). The hippo cascade involves multiple kinases, such as MST1/2 and LATS which ultimately phosphorylate YAP and its close paralog TAZ, triggering their cytoplasmic retention and degradation via the proteasome (5). In contrast, when Hippo is inactive, unphosphorylated YAP/TAZ enter the nucleus and act as transcriptional coactivators by binding to TEAD transcription factors (1,2,6). Deregulation of the upstream hippo tumor suppressors can cause uncontrolled growth and cancer (3,4). Aberrant activation of YAP is known to contribute to cancer initiation, progression and drug resistance and is generally correlated with a poor outcome (7).

Although YAP induces the expression of a number of target genes through binding to promoters, recent studies have shown that YAP primary regulates gene expression by binding to distal transcriptional enhancers, regulatory DNA elements that activate the expression of their distant target genes by the formation of chromatin loops (8–11). Enhancers are highly abundant in the mammalian genome and they play important roles in spatiotemporal regulation of gene expression (12). In cancer cells, enhancers have been shown to be critical to reprogram gene expression and to promote oncogenic activities (13–15). Deregulated enhancers also play a role in the establishment and maintenance of transcriptional addiction, a dependency on transcription factors and chromatin regulators for sustained proliferation of cancer cells (16). YAP is recruited to enhancers mainly by TEAD transcription factors, but is also known to cooperate with other transcription factors and co-activators. For example, many YAP-regulated enhancers contain both TEAD and AP-1 motifs where YAP synergizes with JUN/FOS to promote tumor cell proliferation and transformation (9,17,18). In addition, YAP interacts with the mediator and with the bromodomain protein BRD4 to promote assembly of the pre-initiation complex and trigger transcriptional elongation (19,20).

In previous work, we have shown that YAP cooperates with the B-MYB transcription factor to activate G2/M cell cycle genes (21,22). B-MYB (also called MYBL2) binds to the MuvB core complex to form the Myb-MuvB (MMB) complex, which activates late cell cycle genes (23–27). Mechanistically, by binding to distant enhancers YAP promotes the association of B-MYB with MuvB, leading to the formation of the MMB-complex at the TSS of cell cycle genes and resulting in induction of target gene expression (21). In addition, YAP also enhances the expression of B-MYB, contributing to an increased rate of mitosis and hyperproliferation (21,22,28).

Here, we investigated epigenetic changes by oncogenic YAP using untransformed human breast epithelial MCF10A cells expressing constitutive active YAP5SA. We found that YAP5SA leads to global chromatin changes resulting in thousands of newly opened and closed genome regions. By CRISPR-interference and ChIP, we identified a role for YAP in increasing levels of Ser5-phosphorylated RNA Pol II at the CDC20 promoter. ChIP and biochemical experiments demonstrate that YAP5SA leads to the enrichment of Ser5-phosphorylated RNA Pol II at the promoters of MMB-target genes and provide evidence that this modification is mediated by CDK7. We also demonstrate that YAP leads to closing and inactivation of enhancers bound by the ΔNp63 tumor suppressor. We show that the loss of ΔNp63 chromatin binding and downregulation of ΔNp63 target genes is critical for cell migration by oncogenic YAP.

## MATERIALS AND METHODS

### Cell lines

MCF10A-YAP5SA cells have been described previously (29). MCF10A cells were cultured in DMEM/F-12 supplemented with 5% horse serum, 1% penicillin/ streptomycin, 10 μg/ml insulin, 500 ng/ml hydrocortisone, 20 ng/ml EGF and 100 ng/ml cholera toxin. The expression of YAP5SA was induced in MCF10A-YAP5SA cells by the addition of 0.5 μg/ml doxycycline. MCF10A-YAP5SA-ΔNp63 and MCF10A-ER-YAP2SA-ΔNp63 cells were generated by infection with pINDUCER20-ΔNp63 lentivirus and selection with 1 mg/ml neomycin. For simultaneous induction of YAP5SA and ΔNp63, MCF10A-YAP5SA-ΔNp63 cells were treated with 0.2 μg/ml doxycycline.

### ATAC-seq

100 000 MCF10A cells were washed with ice cold PBS and lysed in ATAC lysis buffer (10 mM Tris pH 7.4, 10mM NaCl, 3 mM MgCl_2_, 0.1% Tween 20 and freshly added protease inhibitor cocktail (Sigma)) by incubating on ice for 10 min. Nuclei were collected by spinning at 500 g for 10 min at 4°C. The transposition reaction mix (25 μl 2× TD buffer, 2.5 μl TDE1 Nextera transposase (Illumina), 16.5 μl PBS, 0.5 μl 1% digitonin, 0.5 μl 10% Tween-20 and 5 μl of nuclease free water) was added to nuclei and incubated at 37°C for 30 min. Next, the reaction was cleaned using the MinElute PCR purification kit (Qiagen), and a PCR with 10–13 cycles was performed using the NEBNext High Fidelity 2X PCR Master Mix (NEB) and Ad1_noMX and Ad2.1–2.12 barcoded primers described in (30). Size selection of the libraries was performed with Agencourt AMPure XP beads (Beckman Coulter). Library quality and fragment size distribution was analyzed on a fragment analyzer (Advanced Analytical). Paired end 2 × 75 bp sequencing was performed on the NextSeq 500 platform (Illumina).

### CUT&RUN

CUT&RUN was carried out as described (31,32). Briefly, 500,000 cells were washed twice with wash buffer ( 20  mM HEPES pH 7.5,  150  mM NaCl and 0.5  mM spermidine containing protease inhibitor) and captured with 20 μl conacavallin A magnetic beads (Polyscience). Cells were resuspended with antibody buffer (wash buffer with 0.005% digitonin and 2 mM EDTA) and incubated with 2 μg of YAP-antibody (Novus Biologicals, NB110-58358) for 2 h at room temperature. Samples were then washed twice with wash buffer containing 0.005% digitonin and incubated with 700 ng/ml of purified protein-A/G-MNase fusion (pA/G-MNase) on a shaker at 4°C for 1 h followed by two more washes in digitonin wash buffer and once in low salt rinse buffer (20 mM HEPES pH 7.5, 0.5 mM spermidine, 0.005% digitonin). To activate protein A-MNase, incubation buffer (3.5 mM HEPES, 10 mM CaCl_2_, 0.005% digitonin) was added and the DNA was digested for 30 min on ice. The incubation buffer was discarded and the reaction was stopped by resuspension in STOP buffer (170 mM NaCl, 20 mM EDTA, 0.005% digitonin, 50 μg/ml RNAseA, 25 μg/ml glycogen). The protein–DNA complex was released by incubation at 37°C for 30 min, the supernatant was transferred to a fresh tube and then digested by proteinase K at 50°C for 1 h. DNA was extracted by ethanol precipitation. Libraries were made with 6 ng of CUT&RUN DNA fragments using the NEBNext Ultra II DNA Library Prep Kit for Illumina (NEB #E7645S). The manufacturer's protocol was adjusted to account for shorter DNA fragments as described previously (33). Briefly, end prep was performed at 20°C for 30 min followed by 50°C for 1 h. The adaptor was used at a concentration of 0.5 μM. 15 PCR cycles were performed at the following conditions: Initial denaturation: 98°C for 30 s. Denaturation 98°C for 10 s; annealing/extension 65°C for 10 s and final Extension 65°C for 5 min.

### Transwell migration assay

MCF10A-YAP5SA and MCF10A-YAP5SA-ΔNP63 cells were starved for 24h in medium supplemented with 0.25% horse serum. Membrane well inlets (OMNILAB) were equilibrated and the top chamber of the transwell was loaded with 500 μl cell suspension (40,000 cells/ml). The lower chamber was filled with 600 μl of MCF10A complete medium. Cells were incubated at 37°C in 5% CO_2_ for 40 h. Transwell inlets were removed and rinsed in PBS and cells on the upper side of the transwell were wiped off with cotton swabs. Migrated cells on the lower side were fixed for 10 min in ice-cold methanol and stained with crystal violet 2% in methanol for 20 min, followed by three washing steps with 1× PBS. Migrated cells were photographed by an inverted Leica DMI 6000B microscope. Crystal violet was solubilized by the addition of 33% acetic acid and measured at 595 nm in a Multiscan Ascent microtiter plate reader (Labsystems).

### Mammosphere assay

Cells were trypsinized and resuspended in mammosphere medium (DMEM/F12 supplemented with 1% penicillin/streptomycin, 52 μg/ml bovine pituitary extract (Thermo Fisher Scientific), 0.5 μg/ml hydrocortisone (Sigma), 5 μg/ml insulin (Sigma), 100 ng/ml EGF (Sigma) and 1× B27 supplement (Thermo Fisher Scientific). Single-cell suspensions were obtained by resuspending the cells 8 times using a 10 ml syringe (25G needle). Finally, 2,000 cells were seeded into the wells of 24-well plates in mammosphere medium. Mammospheres were counted after 7 days.

### siRNA transfection

Double-stranded RNA was purchased from Eurofins or Thermo Fischer Scientific. siRNAs were transfected in a final concentration of 30 nM using RNAiMAX (Thermo Fisher Scientific) according to the manufacturer's protocol. siRNAs are listed in Supplementary Table S3.

### Lentiviral production and infection

Lentiviral particles were generated in HEK293T cells co-transfected with psPAX2, pCMV-VSV-G and a lentiviral vector. Filtered viral supernatant was diluted 1:1 with culture medium and supplemented with 4 μg/ml polybrene (Sigma). Infected cells were selected 48 h after infection with 300 μg/ml neomycin for 7 days.

### CRISPRi

MCF10A-YAP5SA cells expressing Cas9-KRAB were generated by infection with lentiviral vector pHAGE TRE dCas9-KRAB (Addgene #50917; (34)) and selection with neomycin. Individual clones were isolated and screened by immunostaining for homogenous nuclear expression of dCas9-KRAB using HA-antibodies. Guide RNAs were designed with the sgRNA designer of the Broad Institute (https://portals.broadinstitute.org) (35). Guide RNAs were first cloned individually into lenti-sgRNA-blast (Addgene #104993) (36) which contains an U6 promoter and a sgRNA scaffold. To express all five guide RNA cassettes from a single lentiviral vector, we created multiplex-lenti-sgRNA-blast by replacing the KpnI–EcoRI fragment of lenti-sgRNA-blast with annealed oligos SG2790 and SG2791. Next, sgRNA-cassettes were amplified by PCR and assembled into multiplex-lenti-sgRNA-blast to generate multiplex-lenti-sgRNA-blast-CDC20. Guide RNA sequences and PCR primer sequences are listed in Supplementary Table S3. MCF10A-YAP5SA-dCas9-KRAB cells were infected with either multiplex-lenti-sgRNA-blast-CDC20 or with lenti-sgRNA-blast with a nonspecific control guide RNA (lenti-sgRNA-blast-control) and selected with blasticidine.

### RT-qPCR

Total RNA was isolated using peqGOLD TriFast (Peqlab) according to the manufacturer's instructions. RNA was transcribed using 100 units RevertAid reverse transcriptase (Thermo Fisher Scientific). Quantitative real-time PCR reagents were from Thermo Fisher Scientific and real-time PCR was performed using a Mx3000 (Stratagene) and qTower3G (Analytik Jena). Expression differences were calculated as described before (27). Primer sequences are listed in Supplementary Table S3.

### ChIP-qPCR and ChIP-seq

Cells were cross-linked with 1% formaldehyde (Sigma) for 10 min at room temperature. The reaction was stopped by adding 125 mM glycine (Sigma). After cells were lysed for 10 minutes on ice [5 mM PIPES pH 8.0, 85 mM KCl, 0.5% NP40, 1 mM PMSF, protease inhibitor cocktail (Sigma)], nuclei were resuspended in RIPA buffer [50 mM HEPES pH 7.9, 140 mM NaCl, 1 mM EDTA, 1% Triton X-100, 0.1% sodium deoxycholate, 0.1% SDS, 1 mM PMSF, protease inhibitor cocktail (Sigma)]. Chromatin was fragmented to an approximate length of 150 to 300 bp using a Branson sonifier. Antibodies (3 μg for ChIP-qPCR and 9 μg for ChIP-seq) were coupled to protein G dynabeads (Thermo Fisher Scientific) for 6 h at 4°C and then incubated with fragmented chromatin over night at 4°C. Beads were washed in total twelve times with wash buffer I (50 mM Tris–HCl pH8, 0.15 M NaCl, 1 mM EDTA, 0.1% SDS, 1% Triton X-100, 0.1% sodium deoxycholate), wash buffer II (50 mM Tris–HCl pH8, 0.5 M NaCl, 1 mM EDTA, 0.1% SDS, 1% Triton X-100, 0.1% sodium deoxycholate), wash buffer III (50 mM Tris–HCl pH 8, 0.5 M LiCl_2_, 1 mM EDTA, 1% Nonidet *P*-40, 0.7% sodium deoxycholate) and wash buffer IV (10 mM Tris–HCl pH 8, 1 mM EDTA). 1 mM PMSF and protease inhibitor cocktail (Sigma) was added freshly to all buffers. Chromatin was eluted in (10 mM Tris–HCl pH8, 0.3 M NaCl, 5 mM EDTA, 0.5% SDS, 10 μg/ml RNAseA) and crosslink was reversed at 65°C over night. Proteins were digested by adding 200 μg/ml proteinase K at 55°C for 2 h. DNA was purified using the QIAquick PCR Purification Kit (QIAGEN) and eluted in 50 μl EB buffer. ChIP samples were analyzed by qPCR or subjected to library preparation according to the manufacturer's protocol (NEBNext Ultra II DNA Library Prep Kit for Illumina, NEB) using Dual Index Primers (NEBNext Multiplex Oligos for Illumina, NEB). Libraries were sequenced on the NextSeq 500 platform (Illumina). Antibodies used for ChIP and primers for ChIP-qPCR are listed in Supplementary Tables S3 and S4. The GAPDHS promoter was analyzed as a control region (CR).

### Immunoblotting and immunoprecipitation

Cells were lysed in TNN [50 mM Tris (pH 7.5), 120 mM NaCl, 5 mM EDTA, 0.5% NP40, 10 mM Na_4_P_2_O_7_, 2 mM Na_3_VO_4_, 100 mM NaF, 1 mM PMSF, 1 mM DTT, 10 mM ß-glycerophosphate, protease inhibitor cocktail (Sigma)]. Proteins were separated by SDS-PAGE, transferred to PVDF membrane and detected by immunoblotting.

For co-immunoprecipitation of endogenous proteins from nuclear extracts, cells were first lysed in [10 mM HEPES pH 7.4, 10 mM NaCl, 3 mM MgCl_2_, protease inhibitor cocktail (Sigma)] for 20 min on ice. Pelleted nuclei were resuspended in nuclei lysis buffer [20 mM HEPES pH 7.4, 400 mM NaCl, 1.5 mM MgCl_2_, 0.1 mM EDTA, 15% glycerol, 0.5 mM DTT, protease inhibitor cocktail (Sigma)] mixed 1:1 with TNN buffer for another 20 min on ice. After spinning at full speed for 10 min, the supernatant was mixed with 20 mM HEPES pH 7.4 and used for immunoprecipitation overnight at 4°C in constant rotation. Complexes were collected with protein G-dynabeads (Thermo Fisher Scientific) for 2 h at 4°C. Beads were washed three times with nuclei lysis buffer mixed 1:1 with 20 mM HEPES pH 7.4. Proteins were eluted from beads and bound proteins were detected by immunoblotting. Antibodies are listed in Supplementary Table S4.

### Immunostaining

Cells were fixed with 3% paraformaldehyde and 2% sucrose in PBS for 10 min at room temperature. Cells were permeabilized using 0.2% Triton X-100 (Sigma) in PBS for 5 min and blocked with 3% BSA in PBS-T (0.1% Triton X-100 in PBS) for 30 min. Primary antibodies were diluted in 3% BSA in PBS-T and incubated with the cells for 1 hour at room temperature or overnight. After three washing steps with PBS-T, secondary antibodies conjugated to Alexa 488 and 594 (Thermo Fisher Scientific) and Hoechst 33258 (Sigma) were diluted 1:500 in 3% BSA in PBS-T and incubated with the coverslips for 30–60 min at room temperature. Finally, slides were washed three times with PBS-T and mounted with Immu-Mount™ (Thermo Fisher Scientific). Pictures were taken with an inverted Leica DMI 6000B microscope equipped with a Prior Lumen 200 fluorescence light source and a Leica DFC350 FX digital camera.

### PLA

PLA was performed using the Duolink In Situ Kit (Sigma) according to the manufacturer's instructions. Antibodies are listed in Supplementary Table S4. IgG isotype controls were added to the control samples with single specific antibodies. A double IgG control (without specific antibodies) was also performed. Pictures were taken with an inverted Leica DMI 6000B microscope equipped with a Prior Lumen 200 fluorescence light source and a Leica DFC350 FX digital camera. Pictures were analyzed using the open source software Fiji/ImageJ. Nuclei and overlapping PLA dots were separated by using Watershed, and the number of PLA dots per nuclei was determined by using speckle inspector from Biovoxxel toolbox.

### Bioinformatics

Base calling was performed with Illumina's CASAVA software or FASTQ Generation software v1.0.0 and overall sequencing quality was tested using the FastQC script. Read files were imported to the Galaxy Web-based analysis portal (37). Within Galaxy, ATC-seq and ChIP-seq reads were mapped to the human genome (hg19 assembly) using Bowtie2 with default parameters (38). For ATAC-seq properly paired reads were filtered using a Phred score cutoff of 30 and mitochondrial reads were removed. ATACseq peaks were called using Genrich (https://github.com/jsh58/Genrich). Differential peak analysis was performed within Galaxy with Limma-voom (39). Nucleosome calling was carried out with NucleoATAC (40). To create heat maps and density profiles of ATAC-seq, ChIP-seq and CUT&RUN data, DeepTools 2 was used (41). First, normalized bigWig files were created using bamcoverage with a bin size of 10 and normalizing to counts per million reads mapped (CPM). BigWig files were used to compute reads centered on ATAC-seq peak summits (called with Genrich), YAP peak summits or on the TSS of MMB-target genes (42) using computeMatrix. Heatmaps and profiles were created with the plotHeatmap and plotProfile tools. Metagene plots of PolII enrichment across MMB target genes were created with plotProfiles. ChIP-seq and ATAC-seq data were visualized with the Integrated Genome Viewer (43). For ChIP-seq of H3K4me1, H3K4me3, H3K27Ac, B-MYB and LIN9 in control MCF10A cells and YAP5SA cells, we reanalyzed our previously published datasets which are available under GEO accession number GSE115787 (21)

### Quantification and statistical analysis

Statistical analyses were performed using Prism 9 (GraphPad). Tests used to determine statistical significance are indicated in the figure legends. Comparison of two groups was done by a two-sided Student's *t* test. Comparison of multiple groups was performed with ANOVA. *P*-values <0.05 were considered statistically significant. * *P* < 0.05;** *P* < 0.01; *** *P* < 0.001; **** *P* < 0.0001.

## RESULTS

### YAP5SA results in widespread changes in chromatin accessibility

**Figure 1.**
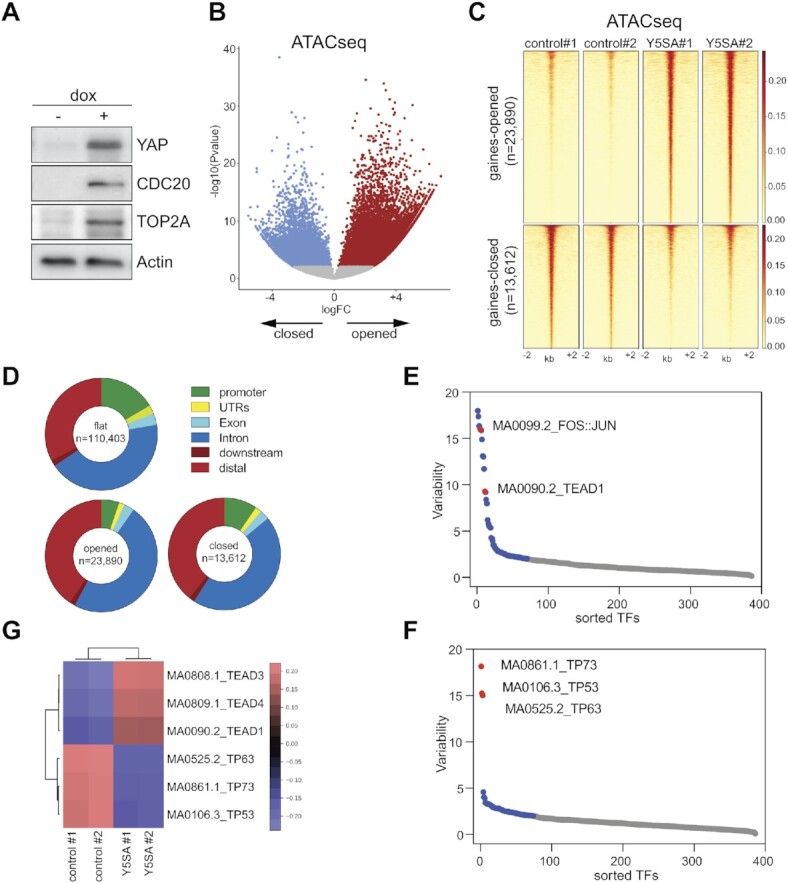
Expression of YAP5SA results in genome-wide changes in chromatin accessibility. (**A**) MCF10A-YAP5SA cells were treated for 48 h with doxycycline. The expression of the indicated proteins was analyzed by immunoblotting. ß-Actin served as a loading control. dox = doxycycline. (**B**) Volcano plot of ATAC-seq data after induction of YAP5SA in MCF10A cells expressing doxycycline-inducible YAP5SA. 23,890 regions were newly opened, 13,612 newly closed, 110,403 were unchanged (flat) at *q* < 0.035. Two biological replicates per condition. (**C**) Heatmap of upregulated and downregulated ATAC-seq peaks in a window of –2 kb to +2 kb centered on the middle of the peak. (**D**) Distribution of ATAC-seq peaks relative to known genes in the genome. (**E**, **F**) ChromVAR chromatin variability scores for ATAC-seq data of YAP5SA expressing MCF10A cells, indicating TEAD and p53-family binding sites as the most variable motifs in gained open (E) and gained closed regions (F), respectively. (**G**) Heatmap showing motif enrichment for open and closed regions.

To recapitulate events in YAP induced tumorigenesis, we used untransformed MCF10A cells expressing doxycycline-inducible, oncogenic YAP5SA. Immunostaining and subcellular fractionation experiments showed that MCF10A cells express very low levels of endogenous YAP, which is predominantly cytoplasmic and inactive (Supplementary Figure S1A, B). The Hippo kinases LATS1 and LATS2 are known to suppress YAP activity by phosphorylating YAP at five serine residues within conserved HXRXXS motifs (44). Mutation of these five serines to alanine (5SA) creates a constitutively active, tumorigenic YAP mutant that cannot be inhibited by the Hippo kinases (44,45). The YAP5SA mutant has been widely used to mimic Hippo pathway inactivation. *In vivo*, the expression of YAP5SA is able to induce liver tumors in mice (46), similar to the liver specific inactivation of the Hippo upstream regulators Sav1 or Mst1,2 (47–49). YAP5SA was robustly induced by doxycycline treatment of MCF10A-YAP5SA cells for 48 h and localized to the nucleus (+dox) (Figure 1A, Supplementary Figure S1A, B). YAP5SA also induced the expression of CDC20 and TOP2A, two genes that are co-regulated by YAP and the MMB complex (Figure 1A and see below). To determine whether YAP leads to changes in chromatin accessibility, we performed assay for transposase-accessible chromatin with sequencing (ATAC-seq). We observed that the expression of YAP5SA resulted in widespread changes in chromatin accessibility in MCF10A cells (Figure 1B, C). Overall, we identified 23,890 newly accessible ATAC-seq peaks ‘opened’ and 13,612 less accessible peaks ‘closed’ in YAP5SA expressing cells compared to control cells. In contrast, 110,403 ‘flat’ peaks were accessible in both conditions and did not change upon expression of YAP5SA. The vast majority of opened and closed peaks mapped to intergenic and intronic regions and only very few peaks were found in promoter regions of annotated genes (Figure 1D).

For an unbiased identification of transcription factor motifs associated with regions with differential chromatin accessibility following expression of YAP5SA we used chromVAR (50). The top motifs enriched in chromatin regions that are gained accessible after expression of YAP5SA correspond to the binding sites for TEAD proteins and for the Activator Protein-1 (AP-1) family of transcription factors (Figure 1E, G, Supplementary Table S1). This is consistent with the previous finding that YAP is recruited to the chromatin by TEAD proteins and that TEAD and AP-1 interact at enhancers to drive the expression of YAP-dependent genes (17). Interestingly, binding motifs for the p53 family of transcription factors were highly enriched in regions that became less accessible in YAP5SA expressing cells, suggesting that the p53 family, comprised of the three members p53, p63 and p73, could play a role in shaping the YAP-mediated enhancer landscape (Figure 1F, G, Supplementary Table S2) (see below). Although most regions that exhibit changes in accessibility are in distal enhancer regions, a fraction of promoters also became more or less accessible upon expression of YAP5SA. We investigated the transcription factor binding sites in these promoter regions and found that the top motif enriched in opened promoters was the TEAD motif (Supplementary Figure S2), whereas in closed promoters the motifs for the zinc finger proteins ZNF93, ZNF610 and EGR1 were highly enriched (Supplementary Figure S3). Notably the binding motif for LIN54, the DNA-binding subunit of MuvB complexes was not enriched in promoters that are opened by YAP5SA. In contrast, the LIN54 motif was the second most enriched sequence motif in the promoters of a set of high confidence MMB target genes which was used as a control for MEME-ChIP (Supplementary Figure S4) (51).

### YAP5SA invades a subset of enhancers leading to their opening and hyperactivation

**Figure 2.**
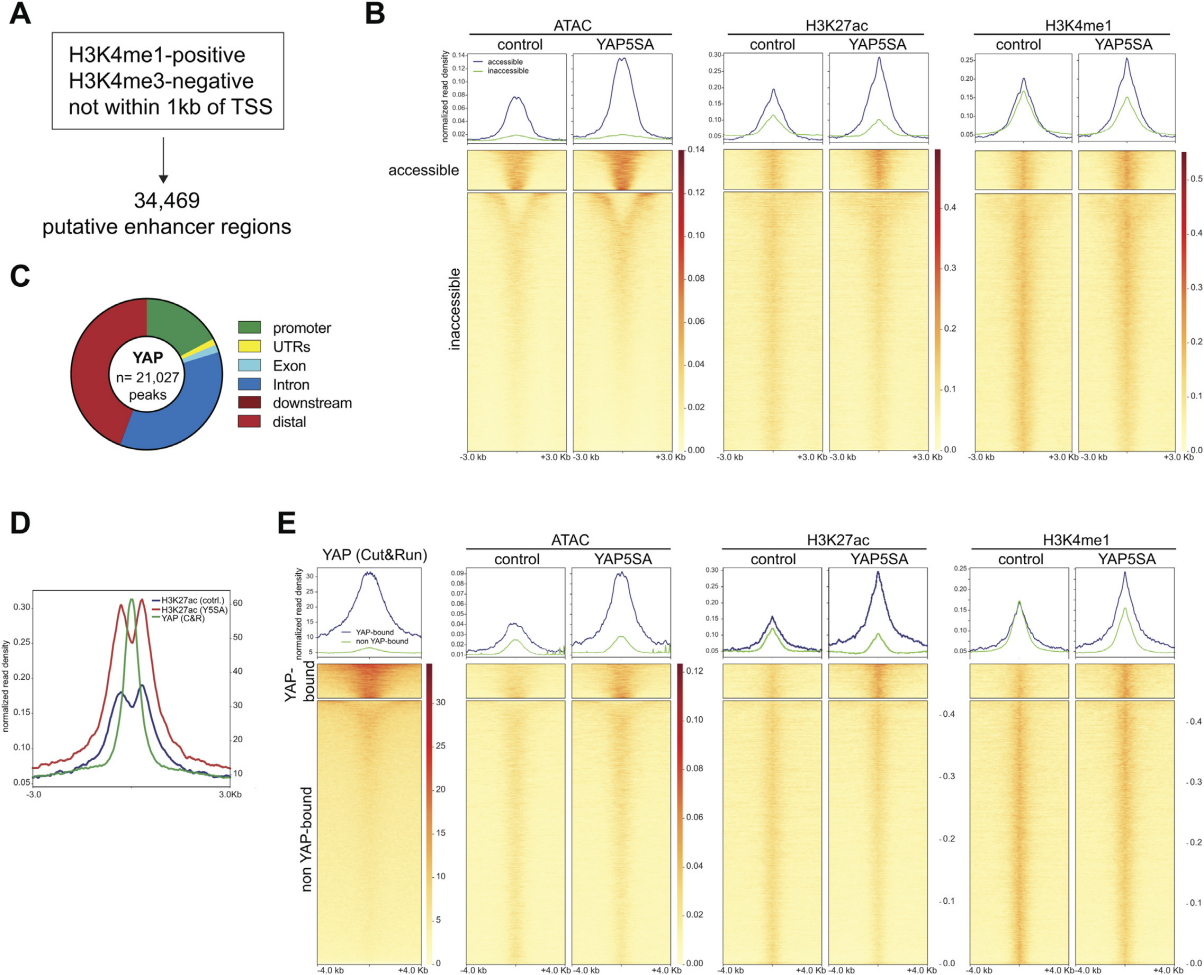
YAP5SA invades a subset of enhancers leading to their opening and hyperactivation. (**A**) Identification of 34 469 putative enhancer regions by analysis of H3K4me1 and H3K4me3 ChIP-seq data. (**B**) Line profiles and heatmap displaying ATAC-seq and ChIP-seq data. *k*-means clustering was performed according to ATAC-seq data. All data sets are arranged to match the order of enhancers found by clustering according to accessibility. (**C**) Binding sites for YAP5SA in MCF10A cells as determined by CUT&RUN. The location of YAP peaks relative in relation to genomic features is shown. (**D**) Line plot of enrichment of YAP and H3K27Ac at YAP enhancer binding sites showing that the YAP-peaks are not randomly distributed in relation to H3K27Ac but that the center of the YAP peak is flanked by two H3K27Ac peaks that gain acetylation when YAP5SA is expressed. (**E**) Line profiles and heatmap displaying YAP CUT&RUN, ATAC-seq and ChIP-seq data. k-means clustering according to YAP enrichment at the enhancer regions identified YAP-bound and non-YAP bound enhancers. All data sets are arranged to match the order of enhancers found by clustering according to YAP enrichment.

We first focused on intergenic regions that become more accessible in cells expressing YAP5SA. We used our previous ChIP-seq data sets to identify enhancers in MCF10A cells. Enhancers were defined as H3K4me1-positive/H3K4me3-negative regions that are not within 1 kb of a transcription start site, which includes active enhancers and enhancers in a primed state before activation (Figure 2A). By this approach we identified a total of 34,469 putative enhancers in control cells and YAP5SA expressing cells. We clustered these enhancers in two categories based on accessibility following YAP5SA expression. We found that previously accessible enhancers are further opened upon expression of YAP5SA (Figure 2B). Opened enhancer regions also become activated as indicated by increased levels of H3K27Ac, which was used as an established indicator of enhancer activity.

We next profiled YAP by Cleavage Under Targets and Release Using Nuclease (CUT&RUN), which has a better signal to noise ratio compared to ChIP-seq and is particularly suited for factors that do not directly bind to DNA (31). We identified 21,027 high confidence YAP peaks compared to the 5,630 peaks previously identified by ChIP-seq in MCF10A cells, confirming the higher sensitivity of the CUT&RUN method. YAP5SA binds mostly to distal and intergenic regions, consistent with previous data (Figure 2C). Plotting the enrichment of H3K27Ac at YAP enhancer binding sites shows that the YAP peaks are not randomly distributed in relation to H3K27Ac but that the center of the YAP peak is flanked by two H3K27Ac peaks that gain acetylation when YAP5SA is expressed (Figure 2D). Clustering of primed and active enhancers based on YAP enrichment by CUT&RUN revealed that accessibility at YAP-bound regions increased upon expression of YAP5SA, while it did not change at the non-YAP-bound regions (Figure 2E). Specifically, of 5,668 YAP peaks that overlap with ATAC-peaks, 2,826 (49.9%) are opened and 138 (2.4%) are closed. Of the 24,397 peaks in the non-YAP-bound cluster, only 3,310 (13.6%) were opened and 3,159 (12.9%) closed.

Notably, baseline accessibility at YAP-bound enhancers in control cells was higher compared to non-YAP-bound regions, suggesting that YAP preferentially binds to regions that are partially accessible but no to completely closed chromatin. It is possible that low levels of YAP expressed in control cells contribute to basal accessibility. YAP-binding not only increased enhancer accessibility but also resulted in an increase in H3K4me1 and in enhancer hyper-activation based on the H3K27Ac ChIP-seq signal. In contrast, non-YAP-bound enhancers were not further activated by YAP5SA. Collectively, these data suggest that YAP5SA invades a significant subset of all enhancers in MCF10A cells leading to their opening and hyperactivation.

### YAP5S does not increase chromatin accessibility at MMB-regulated promoters

To explore how YAP5SA-activated enhancers control gene expression, we focused on cell cycle genes co-regulated by YAP and by the Myb-MuvB (MMB) complex. We have previously shown that MMB and the transcriptional coactivator YAP co-regulate an overlapping set of late cell cycle genes (21). Mechanistically, by binding to distant enhancers, YAP promotes the recruitment of the B-MYB subunit of MMB to MMB-bound promoters, resulting in increased expression of mitotic genes such as CDC20, AURKB or KIF23. Functional studies showed that pro-tumorigenic functions of YAP, such as cell cycle entry and mammosphere formation depend on activation of MMB by YAP (21). Furthermore, the expression of genes coactivated by YAP and B-MYB is associated with poor survival of cancer patients (21).

To investigate whether expression of YAP5SA leads to changes in accessibility at MMB target genes, we plotted the ATAC-seq signal at the TSS of high confidence MMB target genes described by Fisher et al. (51). We found that the TSS of MMB targets is accessible in control cells, although nucleosome occupancy at the -1 nucleosome was slightly reduced when YAP5SA was expressed (Supplementary Figure S5A). In contrast, at the TSS of 1,233 genes with gained ATAC-seq peaks in the promoter (see Figure 1D), accessibility increased and nucleosome occupancy decreased (Supplementary Figure S5B). Nucleosome positioning at the TSS of MMB-target genes and at genes with gained ATAC-seq peaks did not change when YAP5SA was expressed (Supplementary Figure S5A, B). Plotting the ChIP-seq signal for LIN9, a subunit of the MuvB core, revealed that LIN9 is present at the TSS of MMB target genes, but not at genes which opened ATAC-seq regions. Notably, LIN9 was present at the TSS of MMB-target genes before they were activated by YAP5SA, providing a possible explanation for the constitutive accessibility of the TSS. The accessible region at the TSS of MMB target genes also overlaps with the binding sites for B-MYB and FOXM1, which are recruited to the TSS following expression of YAP5SA.

Overall, these data suggest that activation of MMB-target genes is regulated through a different mechanism rather than opening and remodeling of the chromatin at the TSS of these genes.

### YAP regulated enhancers facilitate RNA pol II ser5 phosphorylation at the CDC20 locus

**Figure 3.**
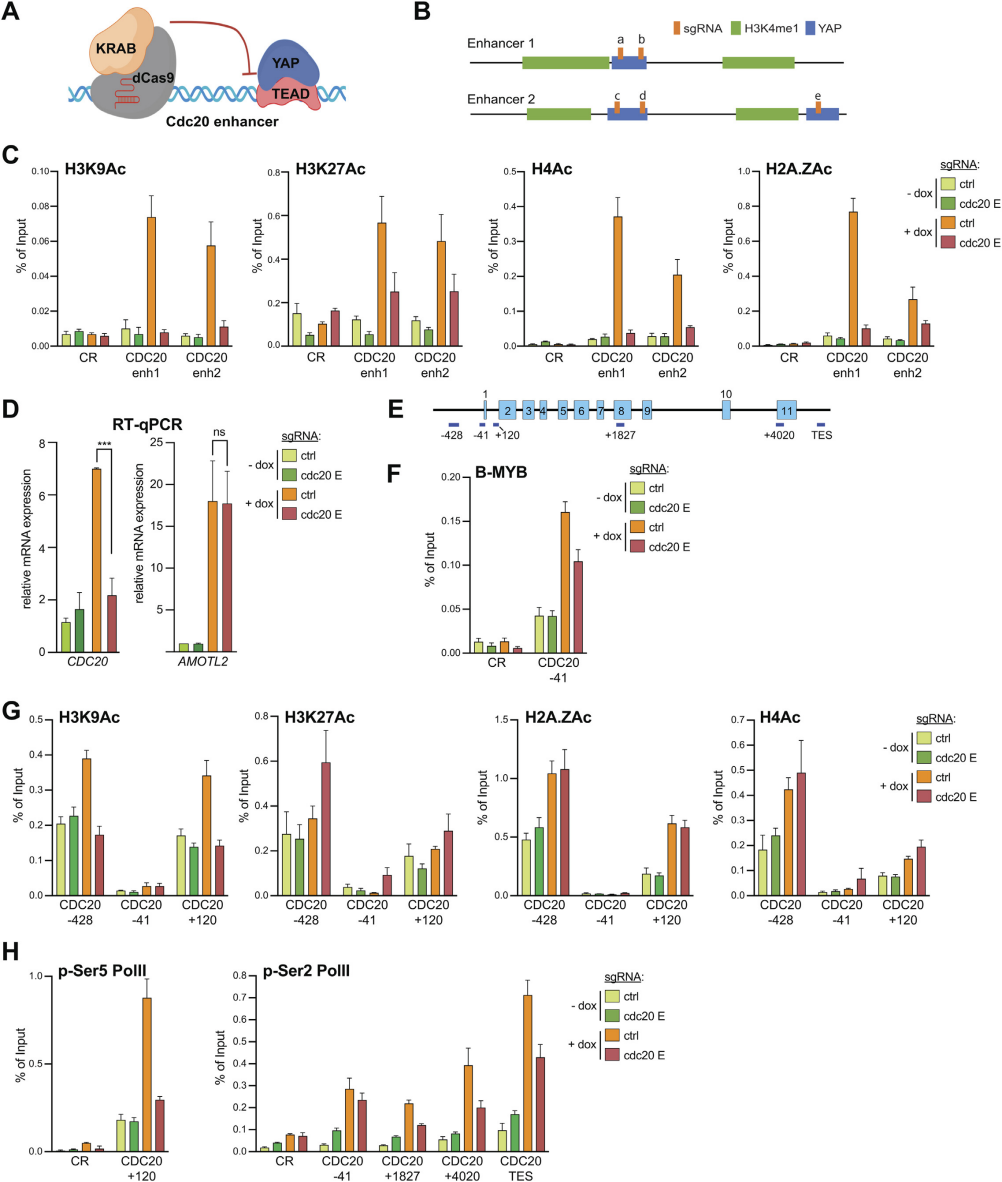
A role for YAP-bound enhancers in histone acetylation and RNA Pol II Ser5 phosphorylation at the CDC20 locus. (**A**) Illustration of the CRISPR-interference (CRSPRi) system to inhibit YAP-bound enhancers. (**B**) Scheme depicting the two CDC20 enhancers (E1 and E2) and position of sgRNAs (A–E) in relation to the YAP (blue) and H3K4me1 (green) peaks as determined by ChIP-seq. (**C**) ChIP-qPCR for H3K9Ac, H3K27Ac, H4Ac and H2A.ZAc at the two CDC20 enhancers before and after YAP5SA induction in cells expressing either a control guide RNA or enhancer-specific guide RNAs demonstrating that targeted CRISPRi interferes with enhancer activation by YAP5SA. CR: negative control region. (**D**) MCF10A-YAP5SA-Cas9-KRAB cells expressing either control or enhancer-specific guide RNAs were treated with doxycycline (+dox) to induce the expression of YAP5SA and Cas9-KRAB or were left untreated (–dox). The expression of *CDC20* and *AMOTL2* relative to *GAPDH* was analyzed by RT-qPCR. Error bars: represent SD. *N* = 3 independent replicates. Student's *t*-test. ****P< 0.0001, ns = not significant. (**E**) Scheme of the CDC20 locus and the position of amplicons used for ChIP-qPCR. (**F**–**H**) ChIP-qPCRs at CDC20 indicated locus for (F) B-MYB, for (G) H3K9Ac, H3K27Ac, H4Ac and H2A.ZAc for (H) p-Ser5 Pol ll, p-Ser2 Pol ll and before and after YAP5SA induction in MCF10A-Cas9-KRAB cells expressing either a control guide RNA or enhancer-specific guide RNAs. CR: negative control region. For all ChIP-qPCR assays the mean and SDs of technical triplicates of a representative experiment (*n* = 2–3 biological replicates) are shown. Panel A was created with Biorender.com.

We next investigated regulation of*CDC20* as an example of a YAP/MMB co-regulated gene. YAP activates*CDC20* expression by binding to two distal enhancers that interact with the *CDC20* promoter by chromatin looping (21) and Supplementary Figure S5C). Accessibility and acetylation of H3K27Ac at these two enhancers increased in cells expressing YAP5SA (Supplementary Figure S5C, D). To better understand how the YAP-bound enhancers control the expression of *CDC20*, we inactivated the enhancers by CRISPR interference (CRISPRi), a CRISPR/Cas9 epigenetic tool based on catalytically-inactive dCas9 fused to the KRAB transcriptional repressor domain (dCas9-KRAB) (Figure 3A). We created MCF10A-YAP5SA cells stably expressing doxycycline-inducible dCas9-KRAB together with either a nonspecific control guide RNA or a set of five guide RNAs that target dCas9-KRAB to the two*CDC20* enhancers (Figure 3B). Western blotting and immunostaining confirmed doxycycline-dependent expression of dCas9-KRAB and YAP5SA (Supplementary Figure S6A,B). To investigate whether dCas-Cas9 prevents enhancer activation, we performed ChIP assays with antibodies specific for acetylated H3K9, H3K27, H4 and H2A.Z, chromatin marks that are associated with transcriptional activation (Figure 3C). Corresponding control ChIP assays with IgG as a control are shown in Supplementary Figure S7. YAP5SA resulted in increased histone acetylation at the two YAP-bound enhancers, which was prevented when Cas9-KRAB was co-expressed with enhancer-specific guide RNAs (Figure 3C). This not only confirms that the*CDC20* enhancers are activated when YAP5SA is expressed, but also shows that Cas9-KRAB targeted to the enhancers interferes with YAP-induced enhancer activation by preventing histone acetylation. Importantly, YAP-mediated induction of *CDC20* mRNA expression was abolished when the enhancers were epigenetically silenced, indicating that the identified enhancers are required for YAP5SA-mediated expression of *CDC20* (Figure 3D). As a control, silencing of the*CDC20* enhancers did not affect induction of *AMOTL2* by YAP5SA, a gene regulated by binding of YAP to the promoter.

We next explored how silencing of the enhancers affects the*CDC20* locus (Figure 3E). By ChIP-qPCR, YAP5SA increased the binding of B-MYB to the*CDC20* TSS, as previously shown (Figure 3F). Silencing the enhancers reduced binding of B-MYB, but did not completely prevent it. This suggests that enhancer-activation only partially controls the recruitment of B-MYB, consistent with the dual role of YAP in promoting the chromatin binding of B-MYB as well as increasing the mRNA expression of *MYBL2* (21).

To further investigate the mechanism by which enhancer activation results in*CDC20* promoter activation, we investigated acetylation of H2A.Z, H3K9, H3K27 and H4 by ChIP (Figure 3G). Acetylated histones showed the expected bimodal distribution around the transcriptional start site of*CDC20*. The signal for H2A.ZAc, H4Ac and H3K9Ac was increased in cells expressing YAP5SA. Notably, only the increase in acetylation of H3K9 was dependent on enhancer activity while acetylation of H2A.Z and H4 was not prevented and H3K27 acetylation was increased by disruption of enhancer activation. Enhanced acetylation of H4 has been reported before after inhibition of CDK7, the kinase that is responsible for phosphorylating RNA Pol II at Ser5 (52). Similarly, depletion of ARID1A, which regulates promoter proximal pausing leads to increased H3K27 acetylation at the +1 nucleosome, likely due to an imbalance between Ser5-phosphorlylated RNA Poll and productive elongation (53). Taken together these data suggest that YAP5SA-mediated*CDC20* enhancer activation has a more direct impact on H3K9-acetylation at the*CDC20* promoter than on acetylation of H4, H2A.Z and H3K27.

To further investigate the functional role of the YAP-bound enhancers on RNA Pol II dynamics, we next focused on the recruitment of RNA Pol II phosphorylated at Ser5 (p-Ser5), which is associated with the transition from initiation to elongation and phospho-Ser2 (p-Ser2), which occurs during pause release and is associated with the elongating RNA Pol II throughout gene bodies. As YAP has primarily been implicated in stimulating transcriptional elongation through controlling the pause-release step (19), we expected an increase in the levels of p-Ser2 and reduced Ser5-phosphorylation of Pol II in cells expressing YAP5SA and possibly accumulation of p-Ser5 Pol II upon enhancer-inhibition. Instead, the expression of YAP5SA caused a robust increase in p-Ser5 Pol II at the TSS of *CDC20* and, importantly, this increase was prevented by CRISPRi-mediated enhancer inhibition (Figure 3H). YAP5SA also caused an enrichment of RNA Pol II p-Ser2 in the*CDC20* gene body and at the TES, which was partially rescued by CRISPRi (Figure 3H). Taken together these observations suggest that enhancer inhibition prevents accumulation of paused Pol II at the *CDC20* promoter by YAP5SA. Inhibition of the enhancer also reduced Pol II Ser2 phosphorylation and transcriptional elongation, but this might be indirect to the effect on initiation and promoter escape.

**Figure 4.**
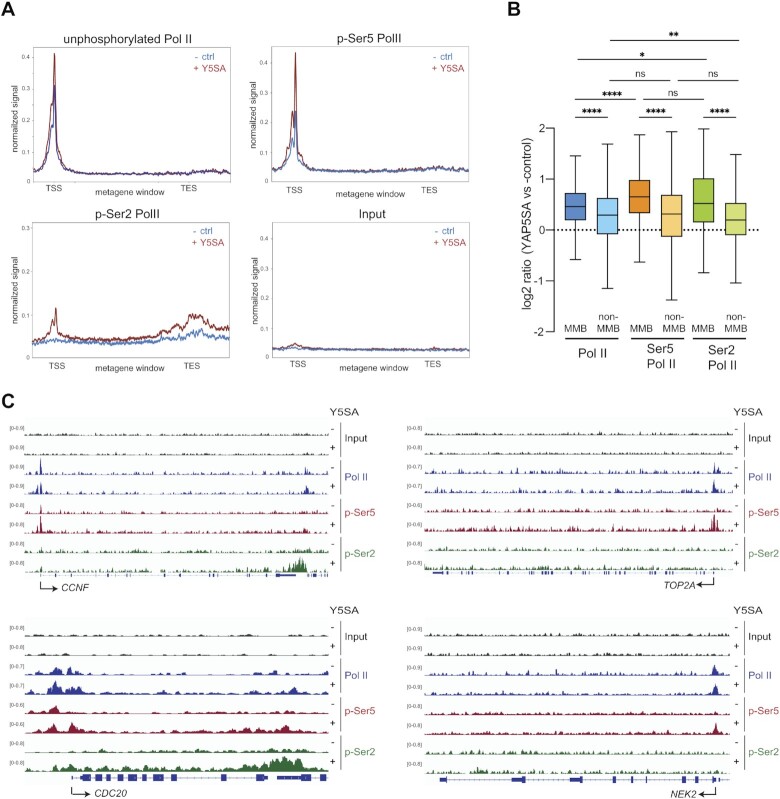
Enrichment of RNA Pol II phosphorylated at Ser5 at MMB-target genes in cells expressing YAP5SA. (**A**) Metagene plots of unphosphorylated RNA Pol II, RNA Pol II phosphorylated at serin 5 (p-Ser5 Pol ll) or phosphorylated at serine 2 (p-Ser2 Pol ll) or Input at MMB-regulated genes. (**B**) Boxplot representing the fold enrichment of unphosphorylated Pol ll and p-Ser5 Pol ll in a window of –500 bp to +500 bp at the TSS or of p-Ser2 Pol lI in a window of TSS + 500 to TES + 3000 bp at MMB-targets and non-MMB target genes in cells expressing YAP5SA vs control cells. Student's *t*-test. **P< 0.01, ****P< 0.0001. (**C**) Genome browser ChIP-seq tracks of *CCNF*,*TOP2A*, *NEK2* and *CDC20* demonstrating enhanced phosphorylation of Ser5 Pol II at the TSS by YAP5SA.

To assess changes in Pol II occupancy not only at the*CDC20* gene but at a larger panel of MMB target genes, we measured genome-wide occupancy of Pol II phosphorylated at Ser5 by ChIP-seq. RNA Pol II p-Ser5 was increased at the TSS-proximal regions of MMB-targets after YAP5SA expression. Using ChIP-seq and an antibody that primarily recognizes unphosphorylated Pol II, we found that levels of unphosphorylated Pol II were also increased at MMB targets by YAP5SA albeit to a lesser extent compared to Ser5 phosphorylated Pol II (Figure 4A). ChIP-seq for RNA Pol II phosphorylated at Ser2 revealed higher levels of p-Ser2 Pol II in gene bodies and at the TES of MMB targets after expression of YAP5SA, consistent with previous studies that linked YAP to transcriptional elongation. Plotting the fold enrichment of unphosphorylated Pol II, p-Ser5 Pol II and p-Ser2 Pol II showed that p-Ser5 boasted the biggest increase at the TSS of MMB-target genes in cells expressing YAP5SA (Figure 4B). A much weaker effect was observed at the TSS of non-MMB target genes. Genome browser tracks of the MMB target genes *CCNF*, *TOP2A*, *NEK2* and *CDC20* illustrating these findings are shown in Figure 4C. Taken together these observations suggest that YAP stabilizes the initiating or paused RNA Pol II at MMB-target genes, extending previously published studies that suggested YAP primarily regulates the pause-release step and transcriptional elongation.

### A role for CDK7 in YAP-mediated activation of MMB-target genes

**Figure 5.**
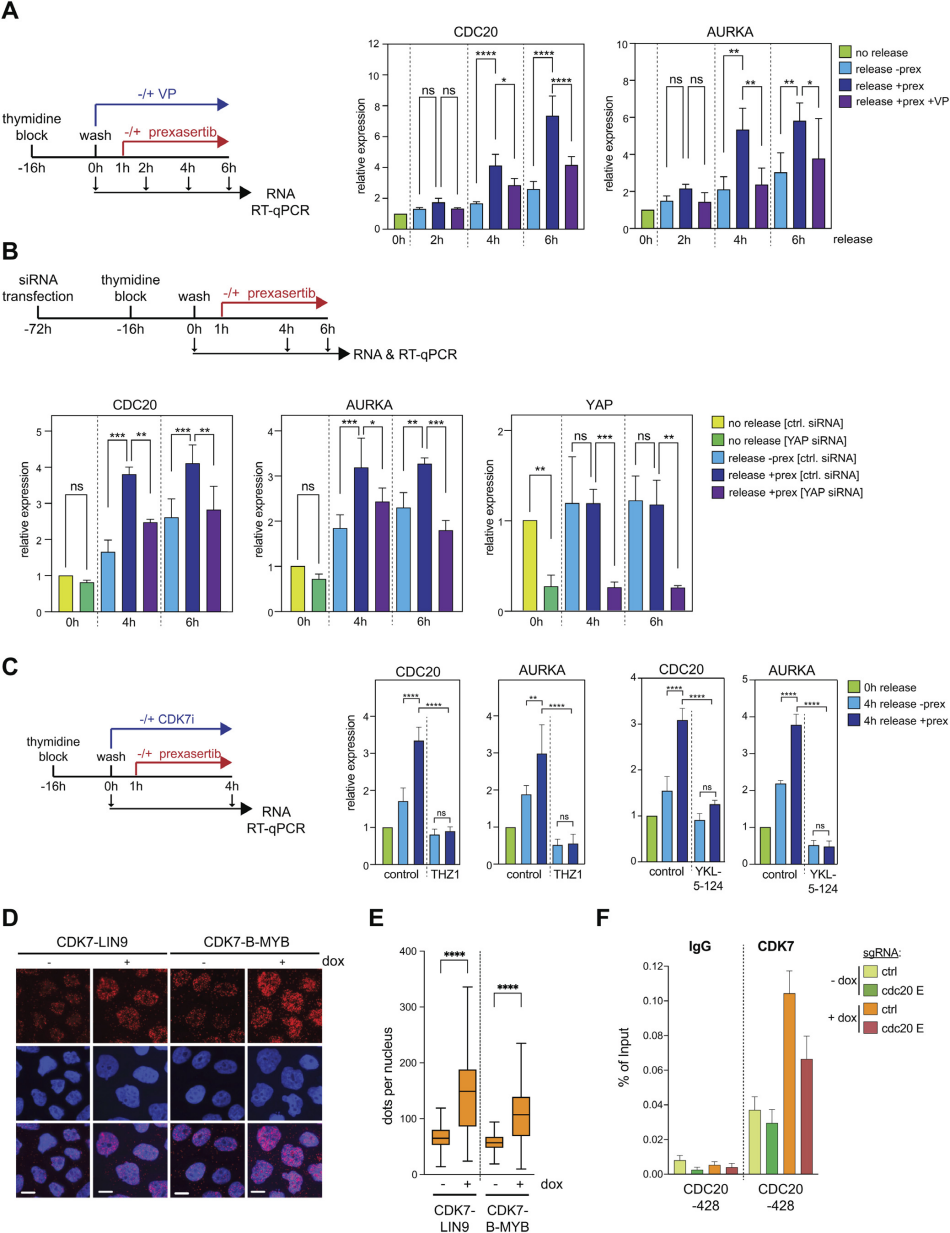
A role for CDK7 in YAP-mediated activation of MMB-target genes. (**A**) Scheme and results of the synchronization experiment with MDA-MB-231 released from a single thymidine block in the presence or absence of verteporfin. One hour after the release, cells were treated with prexasertib or left untreated. 2, 4 and 6 h after the release, RNA was isolated and subjected to RT-qPCR. (**B**) MDA-MB231 cells were transfected with a control siRNA (siCtrl) or with siRNA specific for YAP. Cells were synchronized with a single thymidine block and released for 4 and 6 h. One hour after the release, cells were treated with prexasertib or left untreated. RNA was isolated and subjected to RT-qPCR. (**C**) MDA-MB-231 were released from a single thymidine block in the absence or presence of the CDK7 inhibitor THZ1 or YKL-5-124. One hour after the release, cells were treated with prexasertib or left untreated. Four hours after the release, RNA was isolated and subjected to RT-qPCR. (**D**) Proximity ligation assays (PLA) of CDK7-B-MYB and of CDK7-LIN9 in MCF10A-YAP5SA cells treated with and without doxycycline. Example microphotographs are shown. Scale bar: 10 μm. (**E**) Quantification of the PLA is shown in (D). Shown is a single-cell analysis of one representative replicate (*n* = 3). (**F**) ChIP-qPCRs of CDK7 binding to the CDC20 locus before and after YAP5SA induction in MCF10A-Cas9-KRAB/ YAP5SA cells expressing either a control guide RNA or CDC20-enhancer specific guide RNAs. Precipitations with IgG served as a control. Mean and SDs of technical replicates of a representative experiment (*n* = 3).

Given that YAP5SA increases the recruitment of Ser5 Pol II at MMB-target genes, we next asked whether there is a functional link between CDK7, the main kinase that phosphorylate RNA Pol II and the expression of these genes by using small molecule CDK7 kinase inhibitors. To exclude indirect effects due to long CDK inhibition, we used ATR-CHK1 pathway inhibition as a tool to achieve rapid activation of MMB-target genes. These experiments were performed with MDA-MB-231 cells because they are known to express high levels of endogenous YAP/TAZ activity, which makes them more suitable to study the effects of inhibiting YAP (54). It has previously been shown that the ATR-CHK1 pathway limits the activity of MMB and FOXM1 to prevent premature expression of mitotic genes during DNA -replication in tumor cells (55,56). We first confirmed that inhibition of CHK1 by prexasertib during release from a G1/S block leads to the hyperactivation of MMB-target genes CDC20 and AURKA between 2 and 6 h after the release (Figure 5A). Importantly, co-treatment of cells released from a G1/S block with verteporfin, a drug that disrupts the YAP-TEAD interaction (57), prevented the prexasertib-mediated induction of MMB-targets in S-phase, indicating that this process is YAP-dependent (Figure 5A). The prexasertib-mediated hyperactivation of CDC20 and AURKA was also prevented by siRNA mediated depletion of YAP, further validating that it is a YAP-dependent step (Figure 5B). Importantly, inhibition of CDK7 by THZ1 also abolished hyperactivation of MMB targets by prexasertib, revealing a role for CDK7 in this process (Figure 5C). Because THZ1 is known to have off-target activity towards CDK12/13, we repeated the experiment with the more specific CDK7-inhibitor YKL-5-124 (58). Similar to THZ1, YKL-5–124 also prevented the prexasertib-mediated induction of MMB targets, which confirms a role for CDK7 in activation of MMB targets (Figure 5C). Although CDK7 has recently been reported to stabilize YAP (59), YAP levels were not affected in our experimental system by short-term inhibition of CDK7 (Supplementary Figure S8A). Thus, reduced levels of YAP do not account for the lower induction of MMB target genes when CDK7 is inhibited. Although co-immunoprecipitation experiments showed no detectable biochemical interaction between CDK7 and B-MYB (Supplementary Figure S8B), we reasoned that YAP could enhance the proximity of CDK7 and MMB. To address this possibility, we performed proximity ligation assays (PLA) using antibodies directed at CDK7 and the LIN9 and the B-MYB subunits of MMB. The proximity between CDK7 and LIN9 and between CDK7 and B-MYB was strongly increased when YAP5SA was expressed (Figure 5D, E, Supplementary Figure S8C, D). Consistent with these data, ChIP assays showed that binding of CDK7 to the CDC20 promoter was increased in YAP5SA expressing cells and this was abolished by CRISPRi mediated silencing of the CDC20 enhancers (Figure 5F, Supplementary Figure S8E). YAP5SA also increased the binding of CDK7 to the promoters of the MMB target genes KIF23, TOP2A and NCAPH, but not to the promoters of the DREAM targets RRM1 and CDC6 (Supplementary Figure S8F). Taken together these data suggest a role for the YAP-bound enhancers in the recruitment of CDK7 to MMB-regulated promoters and in the subsequent phosphorylation of Pol II at Ser5.

### YAP5SA triggers the loss of ΔNp63 from enhancers resulting in reduced chromatin accessibility

**Figure 6.**
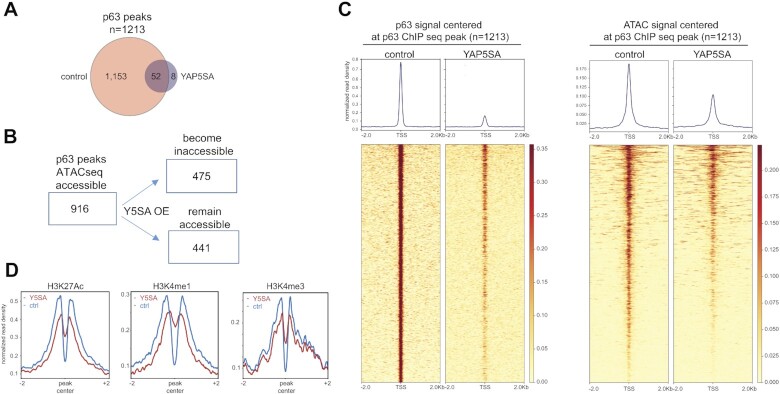
YAP5SA overexpression reduces the chromatin accessibility at ΔNp63 binding sites. (**A**) The genome wide localization of p63 was determined by ChIP-seq with p63 antibodies in MCF10A cells before and after expression of YAP5SA. The number of peaks identified in the two conditions is shown (**B**) Comparison of p63 binding sites with chromatin accessibility obtained by ATAC-seq after the expression of YAP5SA. (**C**) Heatmaps showing p63 enrichment and chromatin accessibility at p63 ChIP-seq peaks before and after YAP5SA expression in a window of –2 kb to +2 kb centered on the middle of the peak. (**D**) Line plots depicting the enrichment of the H3K27Ac, H3K4me1, and H3K4me3 signal at p63 binding sites in MCF10A cells after and before YAP5SA induction.

As described above, binding motifs for the p53 family of transcription factors were highly enriched in regions that became less accessible in YAP5SA expressing cells (see Figure 1F, G). Because p53 is expressed at low levels in unstressed cells and because MCF10A cells are known to express p63 but not p73 (60,61), we next focused on p63 as a possible mediator of the reduced chromatin-accessibility following expression of YAP5SA. By ChIP-seq we observed a strong overall reduction in chromatin-binding of p63 after expression of YAP5SA compared to control cells (Figure 6A). Comparison with ATAC-seq revealed that 916 of the 1,213 identified high confidence p63 binding sites (*q*-value < 0.01) were in open chromatin regions in control cells and about 50% of those became inaccessible after YAP5SA expression (Figure 6B). Loss of p63 binding correlated with reduced accessibility (Figure 6C). We next used our previous ChIP-seq data of histone modifications of control and YAP5SA expressing MCF10A cells to determine whether YAP5SA changes the chromatin status at p63 sites. We observed a decrease in H3K27 acetylation at p63 sites, suggesting reduced activity of p63-bound enhancers upon YAP5SA expression (Figure 6D). Taken together these data suggest that p63-binding is required to keep the chromatin at p63-binding sites accessible. Alternatively, chromatin accessibility at these sites may be reduced indirectly by YAP5SA and accessibility may be required for binding of p63.

### YAP5SA inhibits the expression of ΔNp63

**Figure 7.**
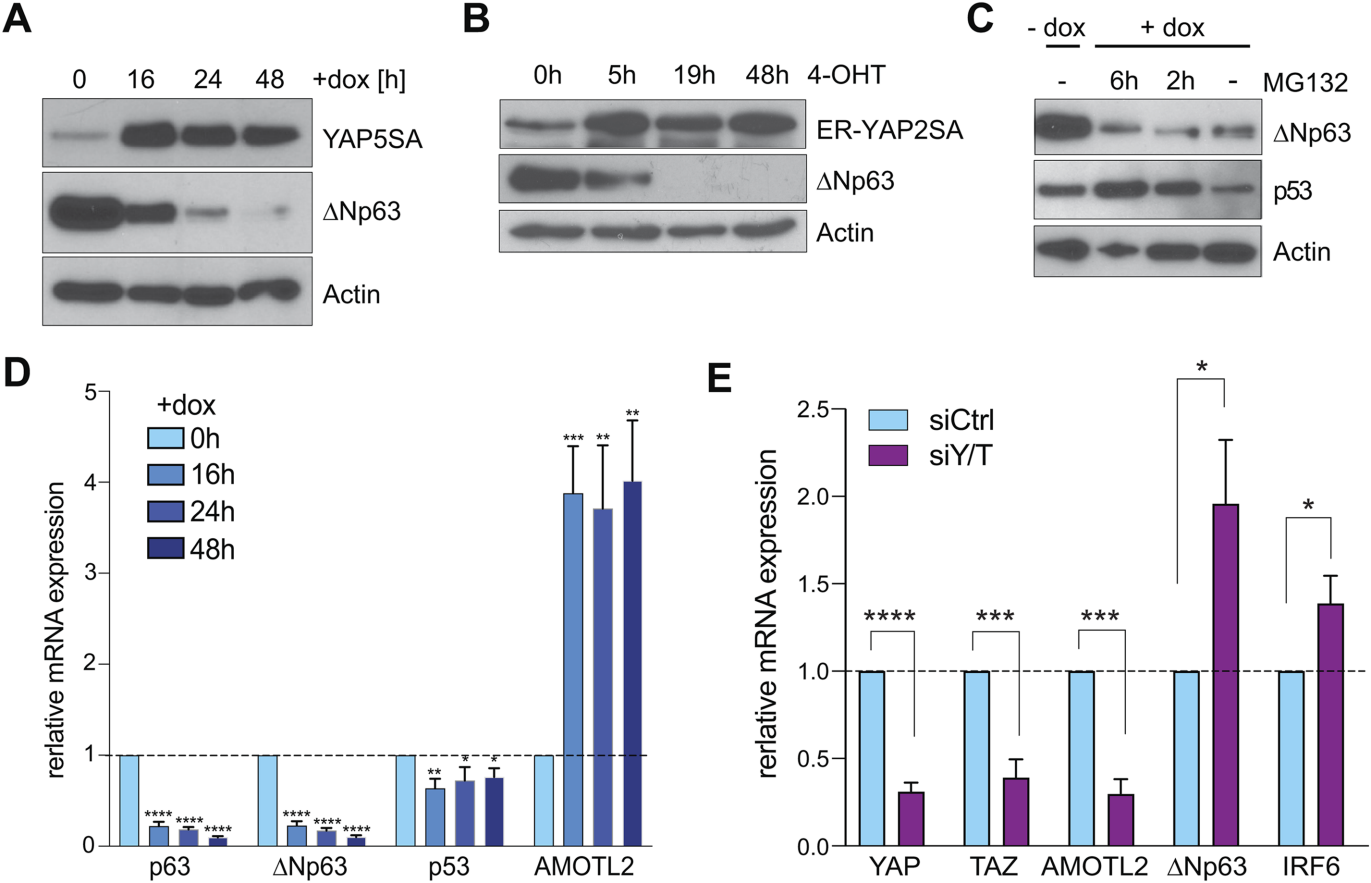
YAP5SA expression leads to downregulation of ΔNp63. (**A**) MCF10A-YAP5SA cells were untreated (-dox) or treated with doxycycline to induce YAP5SA expression for the indicated time points. Expression of the indicated proteins was analyzed by immunoblotting. Actin served as a loading control. (**B**) MCF10A-ER-YAP2SA were untreated or treated with 4-OHT to activate ER-YAP2SA for the indicated time points. The expression of the indicated proteins was analyzed by immunoblotting. Actin served as a loading control. (**C**) MCF10A-YAP5SA cells were untreated (–dox) or treated with doxycycline (+dox) to induce YAP5SA and simultaneously treated with the proteasome-inhibitor MG132. The expression of the indicated proteins was analyzed by immunoblotting. Actin was used as a loading control. (**D**) RT-qPCR in MCF10A-YAP5SA cells before and after YAP5SA induction for the indicated time points. The expression of the indicated genes was analyzed relative to *GAPDH*. Data presented as means from biological triplicates, error bars represent SDs (*n* = 3). (**E**) MCF10A-YAP5SA cells were transfected with a control siRNA (siCtrl) or with siRNAs specific for YAP and TAZ (Y/T) for 72 h. The expression of the indicated genes was analyzed by RT-qPCR. Data presented as means from biological triplicates, error bars represent SDs (*n* = 3). Student's *t*-test. *P< 0.05, **P< 0.01, ***P< 0.001, ns = not significant.

We next tested whether YAP has any effect on the expression p63 that could explain the reduced chromatin-association of p63 in cells expressing YAP5SA. The induction of YAP5SA by doxycycline strongly reduced the protein expression of ΔNp63 in a time-dependent manner (Figure 7A). A robust downregulation of ΔNp63 was also observed in MCF10A cells stably expressing a hormone inducible ER-YAP2SA fusion protein (Figure 7B). In ER-YAP2SA, YAP2SA is fused to a mutant ligand binding domain of the estrogen receptor which sequesters the fusion protein in the cytoplasm in the absence of ligand (62). ER-fusion proteins can be activated by the addition of the ligand, 4-hydroxytamoxifen (4-OHT). After treatment of MCF10A-ER-YAP2SA with 4-OHT to activate ER-YAP2SA, levels of ΔNp63 were sharply reduced, confirming reduced protein expression of ΔNp63 by YAP. The downregulation of ΔNp63 by YAP5SA was also observed in the presence of the pharmacological proteasome inhibitor MG132, suggesting that the reduced abundance of ΔNp63 is not a consequence of its increased turnover by the proteasome (Figure 7C). As a control, MG132 stabilized p53, which is known to be regulated by ubiquitination and proteasome-dependent degradation. Because YAP5SA does not affect the protein stability of ΔNp63, we next asked whether YAP5SA regulates the mRNA levels of ΔNp63. Expression of YAP5SA significantly decreased levels of total p63 and isoform specific ΔNp63 mRNA expression while it had little effect on the mRNA expression of p53 (Figure 7D). Conversely, siRNA mediated co-depletion of endogenous YAP and the related TAZ resulted in upregulation of ΔNp63 (Figure 7E). Because YAP does not directly bind to the ΔNp63 promoter, the regulation of ΔNp63 transcription is likely an indirect effect of YAP regulating other transcription factors involved in ΔNp63 expression.

### Repression of ΔNp63 by YAP5SA is linked to cell migration

To find out how downregulation of p63 by YAP affects gene expression, we integrated our previous RNA-seq data of MCF10A cells expressing YAP5SA with p63 ChIP-seq data. Of the 1,216 genes downregulated by YAP5SA (*q* < 0.05), we identified 97 (8%) genes that were also associated with a nearby p63 ChIP-seq peak. This number likely underestimates the real number of genes co-regulated by YAP and p63 as enhancers and their target genes often interact over long distances that may be missed when target gene identification is based on the nearest binding site. GO analysis showed that the 97 YAP-downregulated/ p63 bound genes were enriched for categories involved in transcription, wound healing, cell spreading, cell adhesion and cell migration (Supplementary Figure S9A).

**Figure 8.**
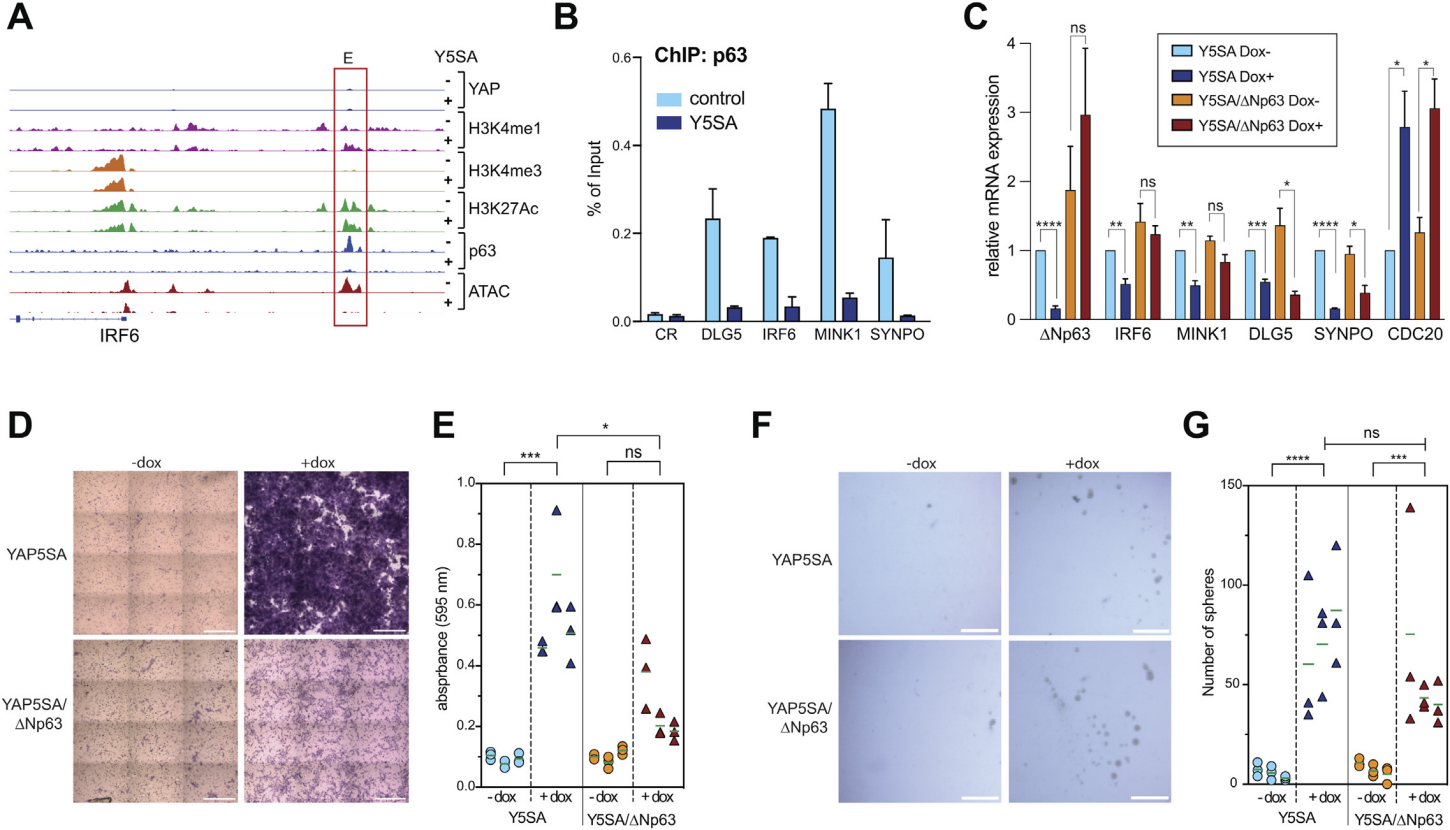
YAP5SA promotes cell migration by inhibiting ΔNp63 expression. (**A**) Genome browser track of the *IRF6* locus, showing ChIP-seq and ATAC-seq data in control MCF10A cells (–) and after expression of YAP5SA (+). E: enhancer. (**B**) ChIP-qPCR demonstrating the binding of ΔNP63 to the enhancers of selected target genes in MCF10A-YAP5SA cells before and after the induction of YAP5SA. Mean and SDs of technical replicates of a representative experiment (*n* = 2). (**C**) MCF10A-YAP5SA and MCF10-YAP5SA-ΔNp63 cells were treatment with doxycycline to either induce YAP5SA or simultaneously induce YAP5SA and ΔNp63. The expression of the indicated genes was analyzed relative to *GAPDH*. Means from three independent biological replicates. Error bars represent SEM. (**D**) Transwell migration assay of MCF10A-YAP5SA and MCF10A-YAP5SA-ΔNp63 treated as described in (C). Representative images from crystal-violet stained transwell layers. Scale bar: 150 μm. (**E**) Quantification of the transwell migration assay shown in (D). Three biological replicates, each performed in technical replicates. (**F**) Primary mammosphere formation in MCF10A-YAP5SA and MCF10-YAP5SA-ΔNp63 treated as described in C. Representative images are shown. (**G**) Quantification of mammospheres. Mean and SDs of three biological replicates. Scale bar: 80 μm. Student's *t*-test. *P< 0.05, **P< 0.01, ***P< 0.001, ns = not significant.

Examples of p63-target genes that are downregulated by YAP and that are involved in cell adhesion and migration are IRF6, DLG5, MINK1 and SYNPO. Genome browser tracks of the*IRF6* and *MINK1* loci illustrates that YAP5SA triggers the loss of p63 binding from the enhancers, which was accompanied by reduced chromatin accessibility and reduced H3K27 acetylation (Figure 8A and Supplementary Figure S9B). ChIP-qPCR verified that p63 binding to the*IRF6*,*DLG5*, *MINK1* and*SYNPO* enhancers is lost upon ectopic expression of YAP5SA (Figure 8B). YAP5SA causes decreased expression of the corresponding p63-target genes while lentiviral restoration of ΔNp63 expression rescued YAP-mediated downregulation IRF6 and partially rescued MINK1 and SYNPO expression (Figure 8C). ChIP assays confirmed that binding of ΔNp63 to the enhancers of the analyzed genes was restored by re-expression of ΔNp63 (Supplementary Figure S9C). Taken together, these data suggest that YAP inhibits the chromatin-binding of ΔNp63 to suppress the expression of ΔNp63 target genes. As the re-expression of ΔNp63 did not rescue all genes to the same extend, additional pathways likely contribute to the downregulation of these targets in response to YAP5SA expression.

To investigate the significance of ΔNp63 downregulation by YAP5SA for cell migration we performed transwell migration assays and found that expression of YAP5SA strongly induced migration of MCF10A cells (Figure 8D, E). Importantly, migration was rescued by restoration of ΔNp63 expression, confirming that downregulation of ΔNp63 expression is directly implicated in YAP-mediated migration. Oncogenic YAP is also known to promote mammosphere formation of MCF10A cells (9). However, and in contrast to migration, YAP-induced mammosphere formation was not rescued by ectopic ΔNp63 expression (Figure 8F, G). Altogether our data suggest that YAP inhibits ΔNp63 mRNA expression resulting in loss of ΔNp63 from enhancers, triggering a decrease in chromatin accessibility and histone acetylation at these sites and leading to the downregulation of ΔNp63 target genes. Thus, loss of ΔNp63 contributes to the global changes in the enhancer landscape upon expression of oncogenic YAP5SA. Downregulation of ΔNp63 enables cell migration in response to YAP5SA expression.

## DISCUSSION

Previous studies have shown that YAP regulates gene expression by binding to distant enhancers (63). In the present study we find that YAP5SA, a well-established constitutive active allele of YAP, leads to widespread global changes in the enhancer landscape of MCF10A cells that promote the oncogenic properties of YAP. These YAP-mediated chromatin changes occur relatively rapidly within two days of YAP5SA induction. We find that YAP invades a subset of partially open enhancers leading to their further opening and hyperactivation. We also identified a link between enhancer activation by YAP and the early steps of transcription by RNA Pol II. Specifically, we demonstrate that YAP-mediated enhancer activation leads to the recruitment of RNA Pol II and the subsequent phosphorylation of Pol II at Ser5 at the TSS of MMB-regulated cell cycle genes. Phosphorylation of Pol II at Ser5 is associated with promoter escape and pausing, which have been identified as fundamental steps in transcriptional regulation (64). Our findings extend previous studies that have linked YAP to Pol II recruitment and post-recruitments steps in transcription, namely the stimulation of pause-release and productive elongation by RNA Pol II through BRD4 and CDK9 (19,20). Transcriptional pausing puts genes in a poised state and acts as a key checkpoint that ensures the release of fully activated and mature Pol II through the promoter region allowing for rapid activation of gene expression. Pausing has also been linked to proper mRNA processing including 5’ capping and splicing of the nascent mRNA (64). We hypothesize that YAP promotes Pol II Ser5 phosphorylation in addition to controlling pause-release in order to balance initiation with elongation and to keep up with the high demand on mRNA processing due to enhanced transcription. The main kinase responsible for CTD Ser5 phosphorylation is CDK7, a subunit of the general transcription factor TFIIH. While co-immunoprecipitations did not show a direct biochemical interaction between CDK7 and MMB, ChIP and PLA experiments provide evidence for increased YAP-dependent recruitment of CDK7 to MMB target genes which correlates with their activation. Although we do not know whether this is a direct effect of YAP or whether it occurs indirectly as a consequence of gene activation, the functional importance of CDK7 in activation of MMB-target genes is demonstrated by the pharmacological inhibition of CDK7. Blocking CDK7 abolished the YAP-mediated induction of MMB-target genes in the MDA-MB-231 cancer cell line, which expresses high levels of endogenous YAP and is known to be addicted to YAP (54).

Although CDK7 has recently been shown to phosphorylate YAP in the nucleus and to prevent its proteasomal degradation (59), in our experimental system, we did not observe any effect of CDK7 inhibition on YAP expression. It is therefore unlikely that the dependence of MMB-target gene expression by YAP on CDK7 is a consequence of the previously described role of CDK7 in YAP turnover. Activation of the MMB-target gene CDC20 was also accompanied by increased histone acetylation at the promoter of this gene. Since H3K9 acetylation at the CDC20 promoter was dependent on enhancer activation, GCN5/PCAF-containing SAGA and ATAC complexes may have a more direct role in MMB-target gene activation than other histone acetyltransferases as they are known to catalyze this modification (65).

In addition to enhancer activation, we also identified regions with decreased chromatin accessibility following expression of YAP5SA, which is explained, at least in part, by a previously unknown function of YAP in downregulating ΔNp63. p63, which plays an important role in mammary epithelial development and self-renewal can be expressed as two isoforms, TAp63 and ΔNp63 (66,67). While ΔNp63 functions as an oncogene by inhibiting the function of p53, TAp63 and TAp73, there is also evidence that reduced expression of ΔNp63α plays roles in EMT, cell motility and cancer metastasis (68–73). ΔNp63 is an unstable protein that is rapidly turned over by proteasomal degradation (74). Although it has previously been reported that YAP physically interacts with ΔNp63 in JHU-22 cells to reduce its half-life (75), YAP does not regulate ΔNp63 protein stability in MCF10A cells, but inhibits the transcription of the ΔNp63 mRNA. The inhibition of ΔNp63 expression by YAP is reminiscent of down-regulation of ΔNp63 expression by oncogenic H-Ras, PI3-K and HER2 signaling (68,69). As it has previously been reported that YAP can suppress PTEN (76), it is tempting to speculate that PI3-K pathway activation contributes to ΔNp63 suppression by YAP. Notably, ΔNp63 binds to its own intronic enhancer to maintain its sustained expression (77). Thus, the signals that lead to downregulation of ΔNp63 expression could be transient and once the positive feedback loop has been interrupted, ΔNp63 could be permanently silenced. More work will be required to determine how YAP suppresses ΔNp63 expression. More importantly, we show that repression of ΔNp63 is pivotal for YAP-induced cell migration. We identified a group of enhancer-associated genes regulated by ΔNp63 and YAP5SA, many of which have previously been shown to be involved in cell migration and adhesion. One example is interferon regulatory factor 6 (IRF6) which was strongly downregulated by YAP5SA and which has been shown to inhibit migration and cell invasion in squamous cell carcinomas and colorectal cancer cells (78,79). Further investigation is required to determine whether suppression of IRF6 contributes to YAP-mediated migration.

**Figure 9.**
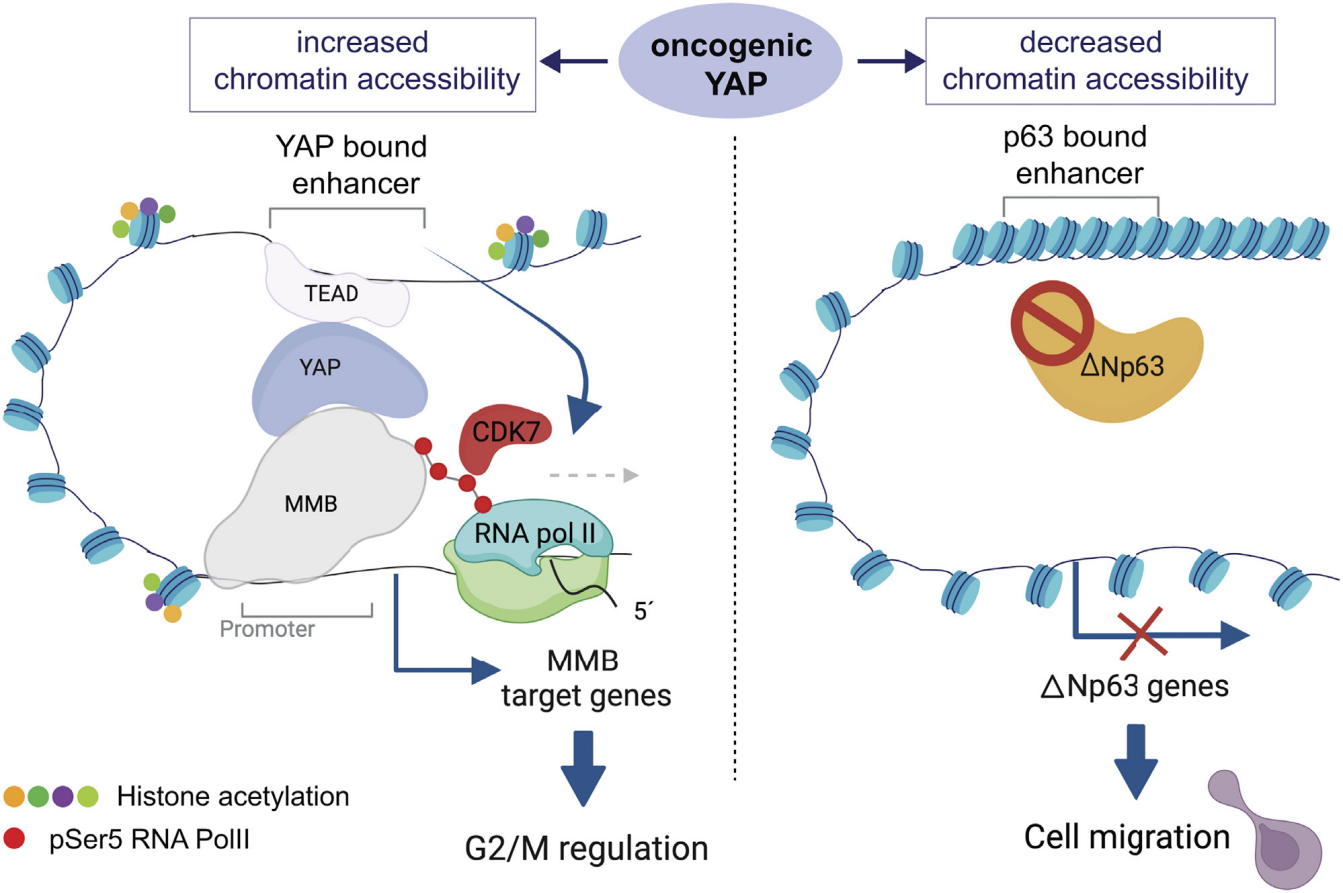
Summary of the results and model. Oncogenic YAP5SA invades a subset of partially accessible enhancers leading to their further opening and hyperactivation. YAP-mediated enhancer activation promotes the recruitment of RNA Pol II and the subsequent CDK7-dependent phosphorylation of RNA Pol II at Ser5 at the TSS of MMB-regulated promoters of cell cycle genes. YAP5SA also leads to less accessible ‘closed’ chromatin regions, which are not directly YAP-bound but which contain b*inding motifs* p63. Diminished accessibility at these regions is a consequence of reduced expression and chromatin-binding of ΔNp63 resulting in downregulation of ΔNp63-target genes and promoting YAP-mediated cell migration. Created with Biorender.com.

In conclusion, we have analyzed global changes in the chromatin status by oncogenic YAP in untransformed MCF10A cells and linked these changes to the expression of genes relevant for cell cycle regulation and migration (Figure 9). Altogether, our findings point to oncogenic activities driven by YAP that may have relevance in cancer biology. We note that a limitation of our study is that it is mainly based on the overexpression of oncogenic YAP, which may not accurately reflect the complexity of tumor development and progression *in vivo*. While overexpression studies in cell culture systems can provide valuable insights into cancer biology, it will be important to confirm our findings in future studies in an *in vivo* setting.

## DATA AVAILIBILITY

ATAC-seq and ChIP-sequencing datasets are available at the NCBI’s Gene Expression Omnibus (80) under the accession number: GSE193704.

## Supplementary Material

gkad107_Supplemental_File
