# Supplementary material for: Oncogenic YAP mediates changes in chromatin accessibility and activity that drive cell cycle gene expression and cell migration
Source: Nucleic Acids Res. 2023 Mar 2;51(9):4266–83. doi: 10.1093/nar/gkad107 (PMC10201438; doi:10.1093/nar/gkad107)
Supplement: gkad107_Supplemental_File [file gkad107_supplemental_file.pdf]

SUPPLEMENTAL MATERIAL

SUPPLEMENTAL FIGURE AND TABLE LEGENDS

Supplementary Figure S1: Subcellular localization of endogenous YAP and overexpressed YAP5SA in MCF10A cells

A) and B) The localization of endogenous YAP (-dox) and of doxycycline-induced YAP5SA (+dox) was investigated by immunostaining (A) and cellular fractionation (B). Immunoblotting for GAPDH (cytoplasmic) and Histone 2B (nuclear), served as controls for the successful fractionization. Endogenous YAP was expressed at very low levels in the cytoplasm, while overexpressed YAP5SA was enriched in the nucleus

Supplementary Figure S2: Motifs enriched in opened promoters

Significantly enriched sequence motifs in promoter regions that become more accessible by YAP5SA (“opened promoters”) as determined by the MEME-ChIP suit.

Supplementary Figure S3: Motifs enriched in closed promoters

Significantly enriched sequence motifs in promoter regions that become less accessible by YAP5SA (“closed promoters”) as determined by the MEME-ChIP suit.

Supplementary Figure S4: Motifs enriched in promoters of MMB target genes

Significantly enriched sequence motifs in promoter regions of high confidence MMB-target genes as determined by the MEME-ChIP suit.

Supplementary Figure S5: YAP5S does not increase chromatin accessibility at MMB-regulated promoters

A) Line plots depicting chromatin accessibility (ATAC), nucleosome signal (NucleoATAC), and the levels of the indicated proteins by ChIPseq at the TSS of YAP-MMB target genes in MCF10A cells with (+) and without (-) expression of YAP5SA. B) Line plots depicting chromatin accessibility (ATAC), nucleosome signal (NucleoATAC), and the levels of the indicated proteins by ChIPseq at the TSS of genes with gained ATAC-peaks in their promoter with and without expression of YAP5SA. C) Genome browser tracks showing chromatin accessibility and YAP-binding to two *CDC20* enhancers (E1 and E2). Long-range interactions between the *CDC20* promoter and enhancers as determined by 4C-seq are indicated by arrows (Pattschull et al., 2019). D) Gene browser tracks of the *CDC20* TSS and enhancer regions depicting the indicating ChIPseq and ATACseq data before and after expression of YAP5SA.

Supplementary Figure S6: Validation of doxycycline-inducible dCas9-KRAB

A) Expression of Cas9-KRAB and YAP5SA in MCF10A cells before and after induction with doxycycline was analyzed by immunoblotting. Actin served as a control. B) Expression of Cas9-KRAB was analyzed by immunostaining with an HA-antibody. Nuclei were stained with Hoechst. +dox: cells were treated with doxycycline to induce the expression of Cas9-KRAB and YAP5SA.

Supplementary Figure S7: Control precipitations with IgG for ChIP assays

Results of control precipitations with IgG for the ChIP assays shown in Figure 3.

Supplementary Figure S8: A role for CDK7 in YAP-mediated activation of MMB-target genes

A) Immunoblotting of the indicated proteins in lysates of MDA-MB-231 cells treated as described in Figure 5B demonstrating that the CDK7 inhibitor THZ1 does not affect the levels of YAP. B) Nuclear lysates of MCF10A-YAP5SA cells treated with (+) or without (-) doxycycline were subjected to immunoprecipitation with CDK7 antibodies. Bound proteins were detected

by immunoblotting. XPD was used as positive control. Immunoprecipitations with nonspecific IgG served as a negative control. C) Example photos of proximity ligation assays (PLA) of the respective antibody controls from Figure 5D, IgG M-R (IgG Mouse- IgG Rabbit control). Scale bar: 10 µm. D) Quantification of PLA shown in C. Shown is a single-cell analysis of one representative replicate (n=3). E) ChIP-qPCRs of CDK7 binding to the CDC20 locus before and after YAP5SA induction in MCF10A-Cas9-KRAB/ YAP5SA cells expressing either a control guide RNA or CDC20-enhancer specific guide RNAs. Shown is the fold-change of all biological replicates compared to the +dox condition. See also Figure 5F. Student's t-test. * = p<0.05, ns = not significant. F) ChIP-qPCRs of CDK7 binding to a control region (CR), the promoters of MMB target genes (KIF23, TOP2A and NCAPH) and DREAM target genes (RRM1 and CDC6). Precipitations with IgG served as a control. Mean and SDs of technical replicates of a representative experiment (n=3 biological replicates).

Supplementary Figure S9: YAP and p63 co-regulated genes involved in migration

A) GO analysis of YAP and p63 co-regulated genes that were identified by integrating p63 ChIPseq and YAP5SA RNAseq data sets. The identified genes are involved in diverse biological processes including development and wound healing. B) Genome browser track of the *MINK1* locus, showing ChIP-seq and ATAC-seq data in control MCF10A cells, before (-) and after expression of YAP5SA (+). E: enhancer. C) MCF10A-YAP5SA and MCF10-YAP5SA-ΔNp63 cells were treated with doxycycline to either induce YAP5SA or simultaneously induce YAP5SA and ΔNp63. Binding of ΔNp63 to the indicated genomic regions was analyzed by ChIP-qPCRs. Control region (CR). Precipitations with IgG served as a control. Shown are the means and SDs of technical triplicates of a representative experiment (n=2 biological replicates).

Supplementary Table 1: Transcription factor motifs associated with regions with increased chromatin accessibility following expression of YAP5SA as determined by chromVAR.

Supplementary Table 2: Transcription factor motifs associated with regions with increased chromatin accessibility following expression of YAP5SA as determined by chromVAR.

Supplementary Table 3: List of oligonucleotide and siRNA sequences

Supplementary Table 4: List of antibodies used in this study

Supplementary Figure S1

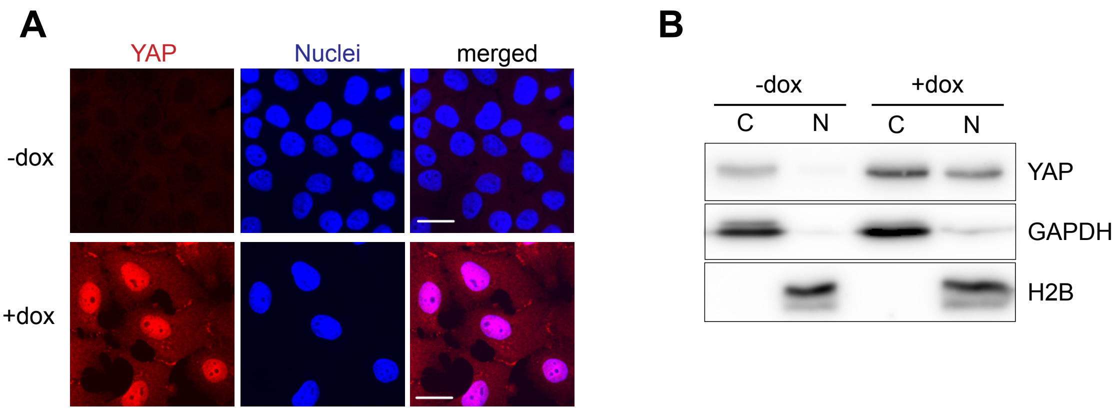

Supplementary Figure S2

significant motifs in opened promoters (E-value ≤ 0.05) found by the programs MEME, STREME and CentriMo

Motif	Discovery/ Enrichment program	E-value	known or similar motifs
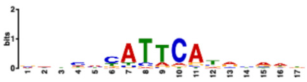	MEME	1.2e-132	TEAD1_full_1<br>TEAD4_DBD<br>TEAD1_full_2
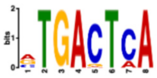	MEME	3.5e-055	Atf3 (MA1988.1)<br>Jun (MA0489.2)<br>BNC2 (MA1928.1)
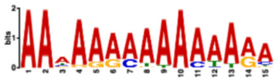	MEME	2.7e-020	ZNF384 (MA1125.1)<br>Mtf1_secondary (UP00097_2)<br>Zfp105_primary (UP00037_1)
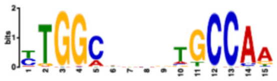	CentriMo	5.8e-011	NFIX_full_1
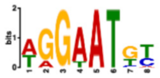	CentriMo	5.4e-004	Sox17_secondary (UP00014_2)
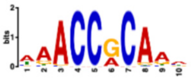	CentriMo	2.6e-003	RUNX3_DBD_2
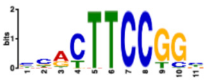	CentriMo	2.9e-003	ELK4 (MA0076.2)
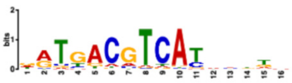	CentriMo	7.4e-003	FOS::JUN (MA1126.1)
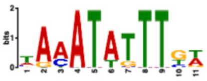	CentriMo	8.5e-003	
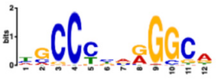	CentriMo	2.7e-002	TFAP2B (MA0811.1)
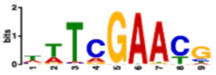	CentriMo	4.9e-002	HSFY2_DBD_2

Supplementary Figure S3

significant motifs in closed promoters (E-value ≤ 0.05) found by the programs MEME, STREME and CentriMo

Motif	Discovery/ Enrichment program	E-value	known or similar motifs
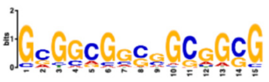	MEME	1.1e-111	ZNF93 (MA1721.1)<br>ZNF610 (MA1713.1)<br>EGR1 (MA0162.4)
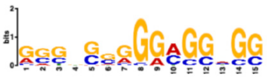	MEME	1.8e-032	ZNF263 (MA0528.2)<br>PATZ1 (MA1961.1)<br>ZNF610 (MA1713.1)
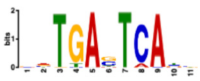	CentriMo	6.5e-027	Atf3 (MA1988.1)<br>JUND (MA0491.2)<br>FOS::JUNB (MA1134.1)
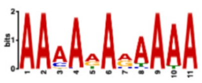	MEME	4.0e-021	ZNF384 (MA1125.1)<br>Srf_secondary (UP00077_2)<br>Mtf1_secondary (UP00097_2)
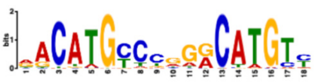	CentriMo	1.2e-018	Tp53_DBD_3
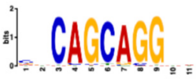	CentriMo	3.1e-009	Zic1::Zic2 (MA1628.1)
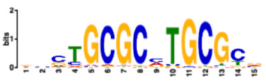	CentriMo	4.3e-009	Nrf1 (MA0506.2)
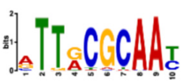	CentriMo	2.6e-008	CEBPG_full
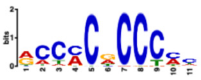	CentriMo	4.7e-008	SP1_DBD
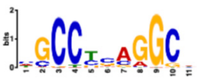	CentriMo	1.1e-004	TFAP2A_DBD_2
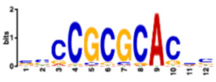	CentriMo	2.1e-004	ZBTB14 (MA1650.1)
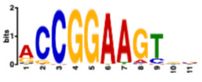	CentriMo	6.7e-004	ELK3 (MA0759.2)
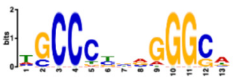	CentriMo	1.7e-003	TFAP2A_DBD_6
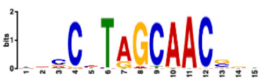	CentriMo	2.1e-003	Rfx4_primary (UP00056_1)
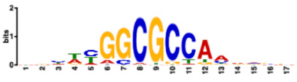	CentriMo	3.1e-003	E2F3_secondary (UP00003_2)
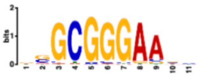	CentriMo	3.5e-003	TFDP1 (MA1122.1)
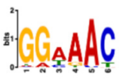	Streme	8.0e-003	TFAP4::FLI1 (MA1967.1)

Supplementary Figure S4

MMB target genes

significant motifs (E-value ≤ 0.05) found by the programs MEME, STREME and CentriMo

Motif	Discovery/ Enrichment program	E-value	known or similar motifs
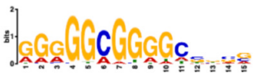	MEME	1.5e-048	SP2 (MA0516.3)<br>SP1 (MA0079.5)<br>SP4 (MA0685.2)
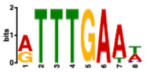	MEME	6.1e-022	LIN54 (MA0619.1)
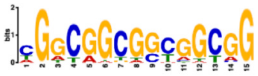	MEME	5.1e-012	ZNF93 (MA1721.1)<br>ZNF740 (MA0753.2)<br>ZNF610 (MA1713.1)
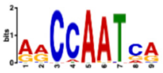	MEME	3.7e-010	NFYA (MA0060.3)<br>NFYC (MA1644.1)<br>Hoxa13 (MA0650.3)
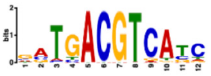	CentriMo	6.4e-008	XBP1_DBD_1
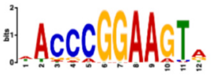	CentriMo	1.0e-003	ELF3_DBD

Supplementary Figure S5

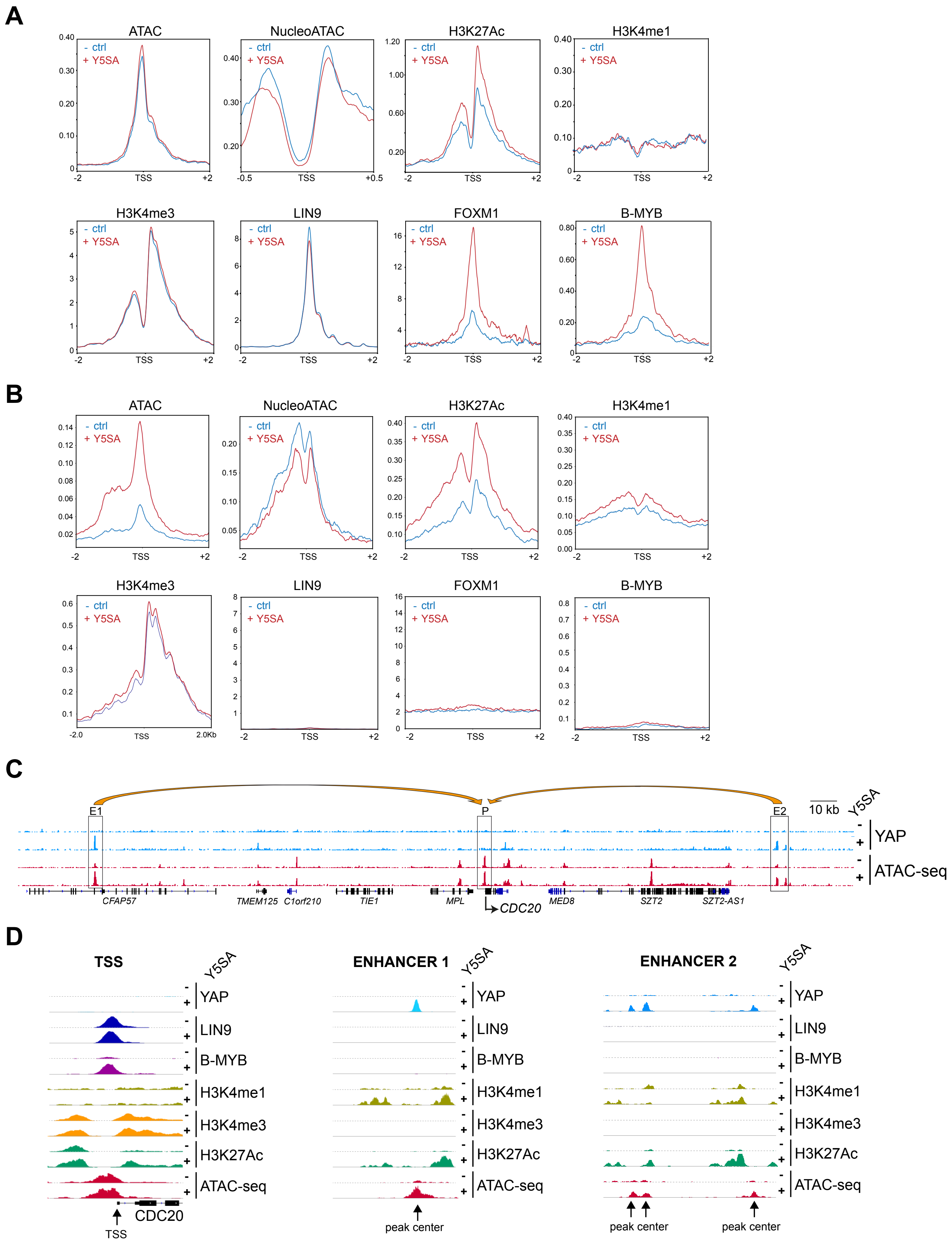

Supplementary Figure S6

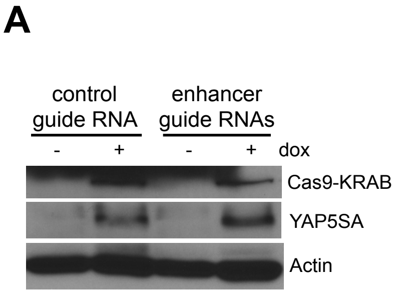

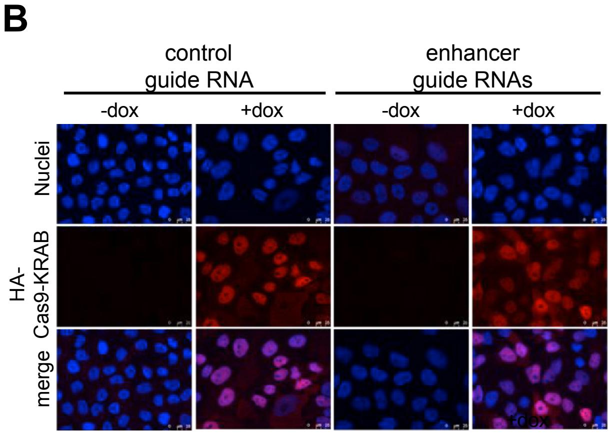

Supplementary Figure S7

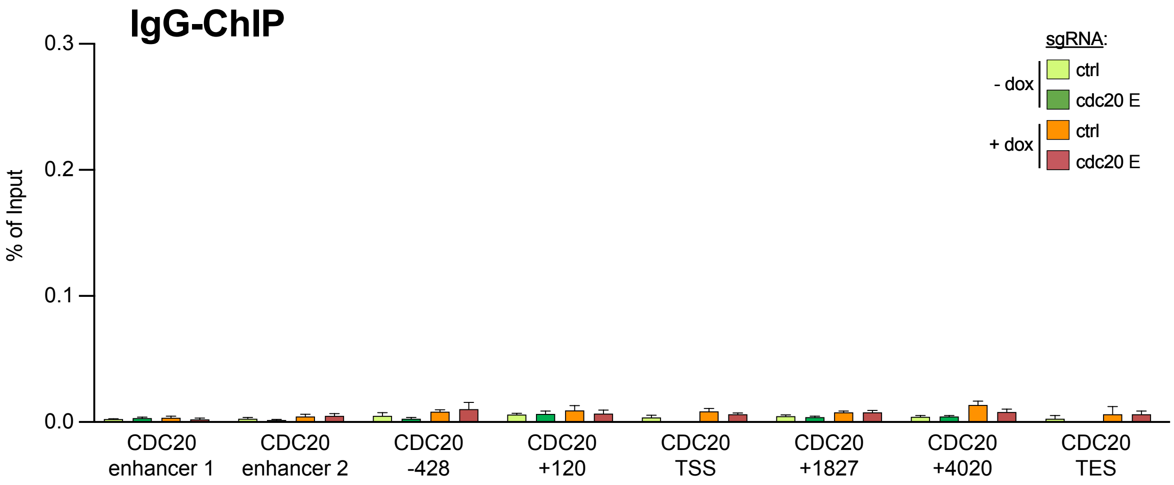

Supplementary Figure S8

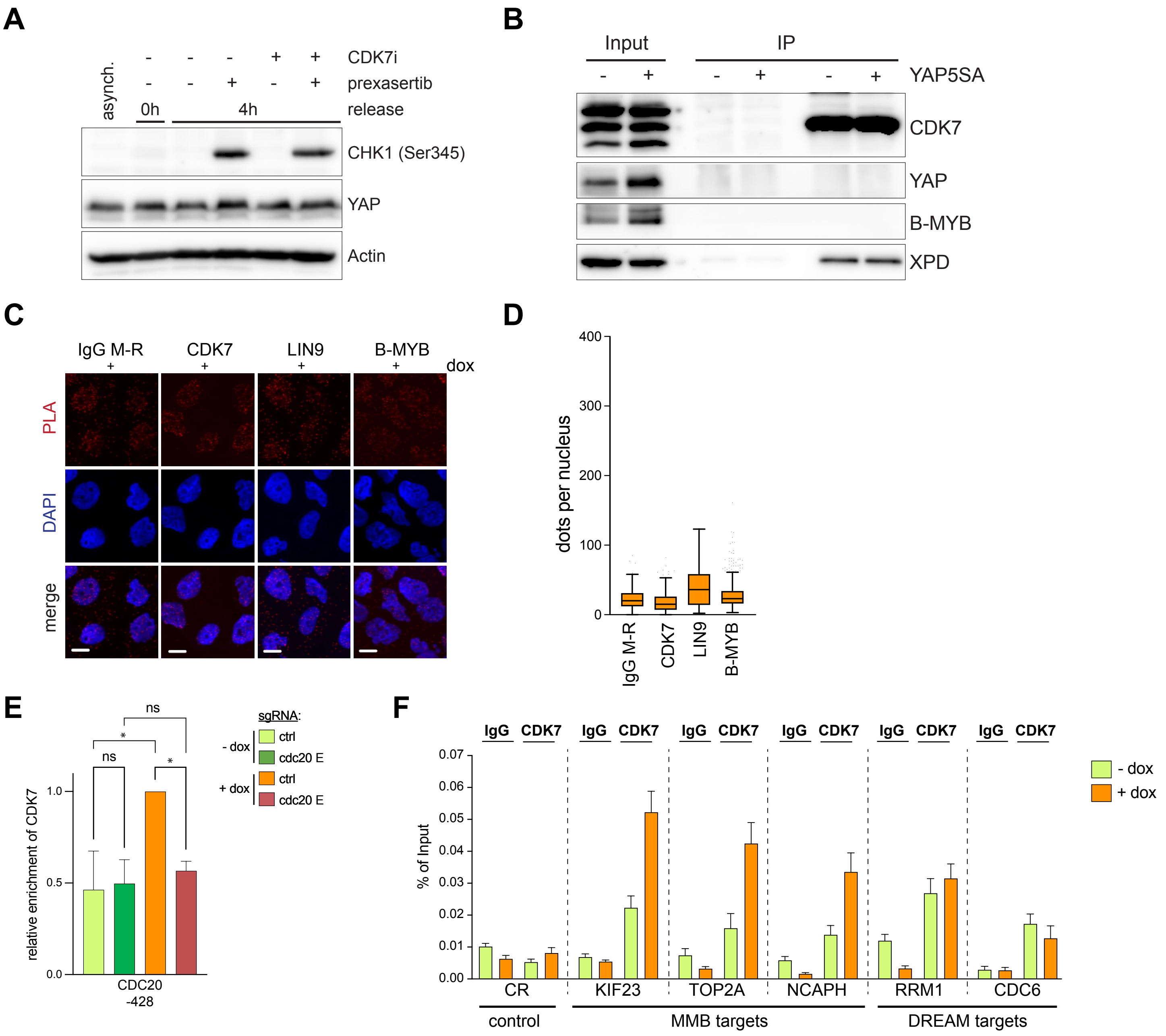

Supplementary Figure S9

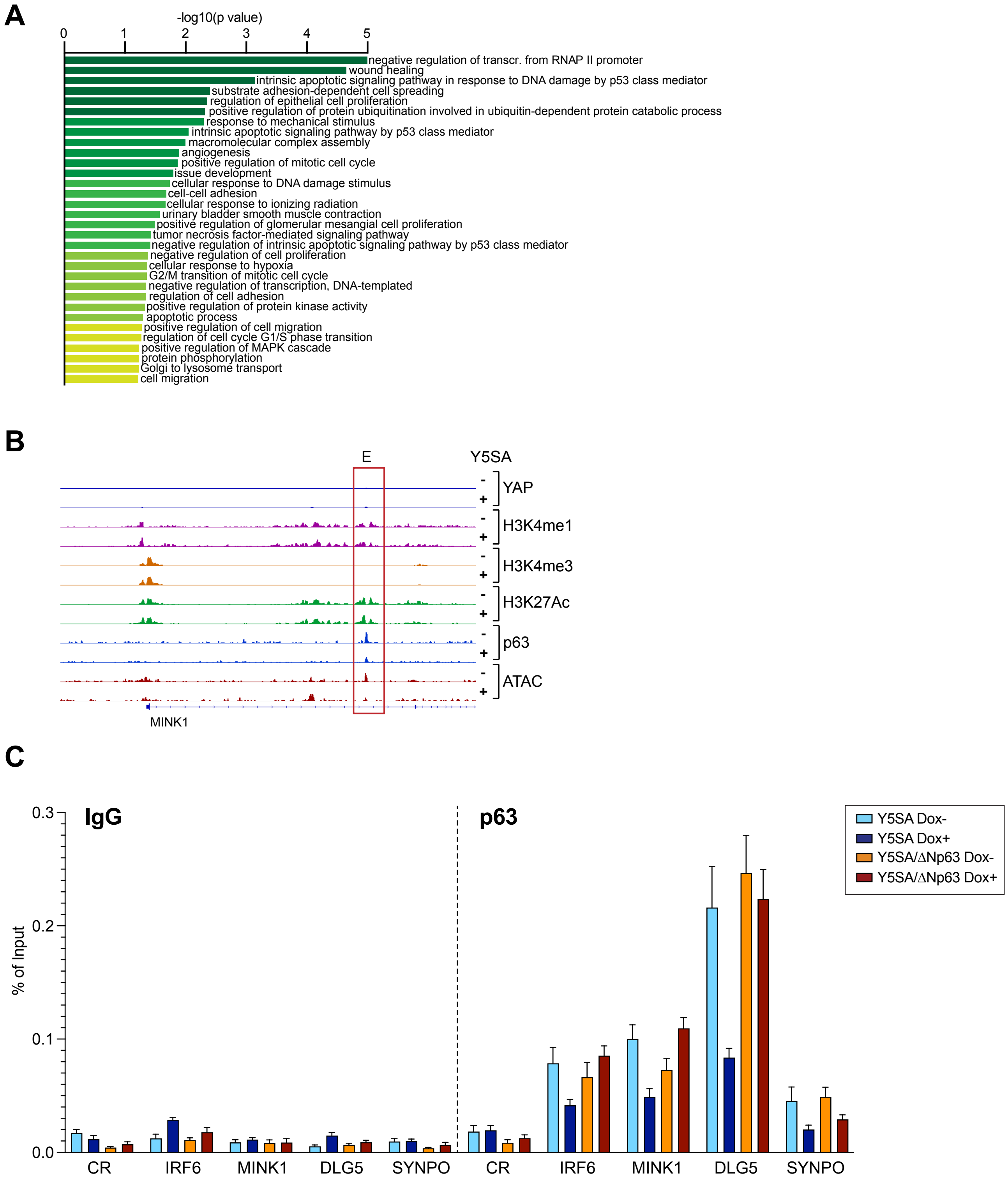

Supplementary Table 1

motifID	name	variability	bootstrap_lower_bound	bootstrap_upper_bound	p_value	p_value_adj
MA0477.1_FOSL1	FOSL1	17.98987005	0.412104515	19.70590534	3.68E-210	1.42E-207
MA0490.1_JUNB	JUNB	17.38345924	0.986094605	19.55546168	3.35E-196	6.47E-194
MA0478.1_FOSL2	FOSL2	16.34383219	0.375602572	17.60316755	2.19E-173	2.82E-171
MA0476.1_FOS	FOS	16.11376173	1.298080863	17.56572437	1.58E-168	1.53E-166
MA0099.2_FOS::JUN	FOS::JUN	15.95608783	0.690824246	17.47256852	3.08E-165	2.38E-163
MA0491.1_JUND	JUND	15.87339004	0.291231996	16.5539259	1.59E-163	1.02E-161
MA0489.1_JUN(var.2)	JUN(var.2)	14.90109462	0.231751543	15.75432283	4.64E-144	2.56E-142
MA0841.1_NFE2	NFE2	13.11174753	0.045376253	13.55814952	1.84E-111	8.87E-110
MA0655.1_JDP2	JDP2	13.01783839	0.298342176	13.7621077	7.24E-110	3.11E-108
MA0462.1_BATF::JUN	BATF::JUN	11.7142826	0.192935075	12.87523254	6.55E-89	2.53E-87
MA0090.2_TEAD1	TEAD1	9.2820071	0.134225343	9.561852092	9.65E-56	3.39E-54
MA0808.1_TEAD3	TEAD3	9.192828361	0.237991935	9.631690525	1.13E-54	3.64E-53
MA0809.1_TEAD4	TEAD4	8.380519858	0.243027288	8.85398153	2.06E-45	6.10E-44
MA0501.1_MAF::NFE2	MAF::NFE2	7.965919449	0.648909857	8.7247638	5.08E-41	1.40E-39
MA0102.3_CEBPA	CEBPA	6.198812782	0.624740355	6.670567706	8.03E-25	2.07E-23
MA0838.1_CEBPG	CEBPG	5.824275418	0.156449774	6.014773596	6.48E-22	1.56E-20
MA0836.1_CEBPD	CEBPD	5.530719251	0.158477186	5.880894151	9.14E-20	2.08E-18
MA0837.1_CEBPE	CEBPE	5.472718793	0.271616082	5.824303884	2.36E-19	5.05E-18
MA0466.2_CEBPB	CEBPB	5.358266978	0.224497468	5.682757202	1.48E-18	3.01E-17
MA0833.1_ATF4	ATF4	4.254687417	0.123321662	4.651085216	9.65E-12	1.86E-10
MA0043.2_HLF	HLF	4.204606312	0.32662258	4.691630266	1.80E-11	3.31E-10
MA0731.1_BCL6B	BCL6B	3.939114435	0.613312842	4.702141048	4.33E-10	7.60E-09
MA0050.2_IRF1	IRF1	3.481110046	0.636756149	3.815790117	6.30E-08	1.06E-06
MA0495.1_MAFF	MAFF	3.456113682	0.196664527	3.723414266	8.12E-08	1.31E-06
MA0088.2_ZNF143	ZNF143	3.209394001	0.021869299	3.466484314	8.92E-07	1.38E-05
MA0707.1_MNX1	MNX1	3.123045702	0.095445762	3.620005498	1.97E-06	2.93E-05
MA0093.2_USF1	USF1	3.016164204	0.155857851	3.196250674	5.11E-06	7.31E-05
MA0843.1_TEF	TEF	2.99423699	0.45011733	3.304157778	6.19E-06	8.53E-05
MA0669.1_NEUROG2	NEUROG2	2.82926127	0.190993349	3.259652276	2.48E-05	0.000330237
MA0842.1_NRL	NRL	2.806778393	0.112200176	3.294644101	2.98E-05	0.000383216
MA0783.1_PKNOX2	PKNOX2	2.779574475	0.565878051	3.235775117	3.71E-05	0.000461632
MA0664.1_MLXIPL	MLXIPL	2.742802399	0.287593046	3.228125518	4.97E-05	0.000586762
MA0639.1_DBP	DBP	2.741528692	0.272985397	3.179249698	5.02E-05	0.000586762
MA0740.1_KLF14	KLF14	2.703844772	0.006850557	3.024336899	6.74E-05	0.000764967
MA0464.2_BHLHE40	BHLHE40	2.665764699	0.032413148	3.276767037	9.04E-05	0.000996783
MA0496.1_MAFK	MAFK	2.629347568	0.044675822	2.718141903	0.000119186	0.001277944
MA0144.2_STAT3	STAT3	2.584176131	0.079675392	2.817016235	0.000167019	0.001742411
MA0470.1_E2F4	E2F4	2.546531687	0.41160081	3.159482076	0.00022018	0.002236563
MA0106.3_TP53	TP53	2.53741503	0.781302891	3.043200901	0.000235263	0.002328499
MA0140.2_GATA1::TAL1	GATA1::TAL1	2.482593539	0.380038668	3.085776405	0.000348514	0.003363157
MA0153.2_HNF1B	HNF1B	2.442348236	0.185257692	2.626607767	0.000462292	0.00435231
MA0662.1_MIXL1	MIXL1	2.387354791	0.194198804	2.99019063	0.000674564	0.006199567
MA0667.1_MYF6	MYF6	2.38027268	0.13397129	2.625139401	0.000707716	0.006352982
MA0668.1_NEUROD2	NEUROD2	2.357410453	0.316491808	2.820583003	0.00082538	0.007240833
MA0025.1_NFIL3	NFIL3	2.342037987	0.68492268	2.673029088	0.000914465	0.00784408
MA0073.1_RREB1	RREB1	2.332212613	0.189599934	2.709955934	0.000976	0.008189913
MA0745.1_SNAI2	SNAI2	2.288901674	0.046952212	2.325702753	0.001295854	0.010361119
MA0710.1_NOTO	NOTO	2.2880999	0.521683956	2.976927133	0.001302599	0.010361119
MA0137.3_STAT1	STAT1	2.284287437	0.083483608	2.618042884	0.001335122	0.010361119
MA0634.1_ALX3	ALX3	2.283479014	0.058153621	2.8284046	0.001342114	0.010361119
MA0037.2_GATA3	GATA3	2.262445176	0.059302465	2.651830949	0.001536381	0.011404676
MA0766.1_GATA5	GATA5	2.262445176	0.059302465	2.651830949	0.001536381	0.011404676
MA0163.1_PLAG1	PLAG1	2.233971357	0.1725478	2.579377024	0.001840834	0.013406831
MA0666.1_MSX1	MSX1	2.223639488	0.174948716	2.504036932	0.001964406	0.013786557
MA0708.1_MSX2	MSX2	2.223639488	0.174948716	2.504036932	0.001964406	0.013786557
MA0036.2_GATA2	GATA2	2.219754183	0.141939664	2.57912267	0.002012817	0.013874063
MA0048.2_NHLH1	NHLH1	2.199771644	0.49930078	2.816697241	0.002279597	0.01543727
MA0139.1_CTCF	CTCF	2.179277024	0.024897094	2.215454754	0.002586609	0.01721433
MA0525.2_TP63	TP63	2.166658745	0.40705733	2.588324754	0.002794023	0.018279543
MA0119.1_NFIC::TLX1	NFIC::TLX1	2.156060838	0.043983426	2.429454321	0.002979871	0.019170505
MA0823.1_HEY1	HEY1	2.145642542	0.035885644	2.315959209	0.003173521	0.020081625
MA0527.1_ZBTB33	ZBTB33	2.121977153	0.106428285	2.438756656	0.003656809	0.022766588
MA0861.1_TP73	TP73	2.103991443	0.502359539	2.500972983	0.004067958	0.024924313
MA0831.1_TFE3	TFE3	2.079914601	0.157529161	2.376729082	0.004684131	0.028251164
MA0820.1_FIGLA	FIGLA	2.064366059	0.075917451	2.393049767	0.005125821	0.030439488
MA0141.3_ESRRB	ESRRB	2.055787184	0.143667774	2.316321879	0.00538535	0.031496139
MA0763.1_ETV3	ETV3	2.046213262	0.346786945	2.828734645	0.005688967	0.032775243
MA0057.1_MZF1(var.2)	MZF1(var.2)	2.036242727	0.332551571	2.31152391	0.006021519	0.034180978
MA0759.1_ELK3	ELK3	2.023235765	0.3646221	2.65596194	0.006481681	0.036259841
MA0692.1_TFEB	TFEB	2.019404261	0.187099823	2.358250959	0.006623147	0.036521923
MA0647.1_GRHL1	GRHL1	1.993197353	0.158939991	2.210546543	0.007667599	0.041685818
MA0862.1_GMEB2	GMEB2	1.975268992	0.53332262	2.25098666	0.008464906	0.0453813
MA0754.1_CUX1	CUX1	1.95357102	0.446095855	2.350523039	0.009528568	0.050383936
MA0105.4_NFKB1	NFKB1	1.947599487	0.450222838	2.334276134	0.009841505	0.051335416
MA0160.1_NR4A2	NR4A2	1.935930357	0.175266655	2.23543077	0.010479581	0.05393491
MA0644.1_ESX1	ESX1	1.920116546	0.658525051	2.527451241	0.011402963	0.057915049
MA0028.2_ELK1	ELK1	1.908904576	0.576133951	2.573006083	0.012100712	0.060660711
MA0761.1_ETV1	ETV1	1.89980918	0.589379693	2.598242444	0.012694273	0.062820375
MA0875.1_BARX1	BARX1	1.868159381	0.445086137	2.491696868	0.01496539	0.073122031
MA0081.1_SPIB	SPIB	1.861686584	0.115576004	1.957679903	0.015471651	0.073851841
MA0597.1_THAP1	THAP1	1.861362288	0.38396557	2.300156253	0.015497407	0.073851841
MA0755.1_CUX2	CUX2	1.857820502	0.298601667	2.230760688	0.015781163	0.074286939
MA0526.1_USF2	USF2	1.839486985	0.12564933	1.9801541	0.017324152	0.080567743
MA0782.1_PKNOX1	PKNOX1	1.834989266	0.483757941	2.084711056	0.017722301	0.081438194
MA0660.1_MEF2B	MEF2B	1.816868841	0.088270151	2.225855137	0.019408595	0.088137853
MA0610.1_DMRT3	DMRT3	1.807910207	0.03527888	2.127607791	0.020292818	0.091081717
MA0046.2_HNF1A	HNF1A	1.802460614	0.120525909	2.099172117	0.020847684	0.092496621
MA0653.1_IRF9	IRF9	1.78971289	0.359677226	2.173575024	0.022197419	0.09736595
MA0630.1_SHOX	SHOX	1.77793367	0.080560439	2.116789193	0.023511353	0.098645461
MA0702.1_LMX1A	LMX1A	1.77793367	0.080560439	2.116789193	0.023511353	0.098645461
MA0703.1_LMX1B	LMX1B	1.77793367	0.080560439	2.116789193	0.023511353	0.098645461
MA0721.1_UNCX	UNCX	1.77793367	0.080560439	2.116789193	0.023511353	0.098645461

MA0824.1_ID4	ID4	1.771030784	0.219383946	2.083859037	0.024312213	0.100908757
MA0663.1_MLX	MLX	1.765478304	0.201561741	2.219871957	0.024973406	0.101567786
MA0714.1_PITX3	PITX3	1.765280424	0.321980425	2.216000538	0.024997253	0.101567786
MA0830.1_TCF4	TCF4	1.74707966	0.175072321	2.167151744	0.027276409	0.109673894
MA0636.1_BHLHE41	BHLHE41	1.726530552	0.011856692	2.103464129	0.030062185	0.119628902
MA0697.1_ZIC3	ZIC3	1.722353768	0.250689294	2.004518834	0.030657261	0.12075207
MA0481.1_FOXP1	FOXP1	1.715669943	0.161988917	2.084680736	0.031630464	0.122903086
MA0902.1_HOXB2	HOXB2	1.71376972	0.199415302	2.112424527	0.031911915	0.122903086
MA0259.1_ARNT::HIF1A	ARNT::HIF1A	1.712116079	0.066710169	2.417190417	0.032158579	0.122903086
MA0797.1_TGIF2	TGIF2	1.70929679	0.192630426	1.845526867	0.032582866	0.123303786
MA0818.1_BHLHE22	BHLHE22	1.705536774	0.206782234	2.022262495	0.033156142	0.124255057
MA0825.1_MNT	MNT	1.689439988	0.230456163	2.018573414	0.035708382	0.132533034
MA0719.1_RHOXF1	RHOXF1	1.677321532	0.007961122	1.752303571	0.037738061	0.138732299
MA0659.1_MAFG	MAFG	1.662268731	0.073721475	1.960318871	0.040394194	0.145419409
MA0652.1_IRF8	IRF8	1.659383319	0.027704318	1.940378873	0.040920914	0.145419409
MA0778.1_NFKB2	NFKB2	1.658615503	0.353790462	1.999096085	0.041062045	0.145419409
MA0077.1_SOX9	SOX9	1.65860472	0.231994128	1.839023343	0.04106403	0.145419409
MA0122.2_NKX3-2	NKX3-2	1.648813182	0.014484017	1.885940259	0.042900046	0.150540163
MA0522.2_TCF3	TCF3	1.621945918	0.049396111	1.874579628	0.048294554	0.167943224
MA0658.1_LHX6	LHX6	1.594690882	0.013036269	1.95504662	0.054332064	0.18724076
MA0600.2_RFX2	RFX2	1.592625136	0.036832616	1.736996217	0.054814005	0.18724076
MA0775.1_MEIS3	MEIS3	1.58497886	0.290554512	1.946398714	0.056628649	0.191742619
MA0888.1_EVX2	EVX2	1.571426395	0.206016087	1.882593345	0.05996623	0.19974202
MA0863.1_MTF1	MTF1	1.571189	0.000716482	1.85474069	0.060026099	0.19974202
MA0472.2_EGR2	EGR2	1.555885673	0.070428358	1.742161894	0.063989893	0.209259002
MA0642.1_EN2	EN2	1.554644965	0.160596305	2.189350261	0.064320385	0.209259002
MA0497.1_MEF2C	MEF2C	1.552407597	0.209198276	1.770604531	0.064919871	0.209259002
MA0718.1_RAX	RAX	1.55190704	0.307032721	2.020979092	0.065054612	0.209259002
MA0027.2_EN1	EN1	1.546606644	0.085455001	1.755468817	0.066495375	0.210387007
MA0068.2_PAX4	PAX4	1.546606644	0.085455001	1.755468817	0.066495375	0.210387007
MA0855.1_RXRB	RXRB	1.54233739	0.17600491	1.945470769	0.067674586	0.212377155
MA0734.1_GLI2	GLI2	1.532636318	0.26485361	1.86085313	0.070417144	0.219201754
MA0871.1_TFEC	TFEC	1.529596518	0.305181387	1.87824416	0.071294747	0.220158179
MA0155.1_INSM1	INSM1	1.526148104	0.210140292	1.783703969	0.072300973	0.220581072
MA0765.1_ETV5	ETV5	1.525217046	0.323383323	1.914584527	0.072574601	0.220581072
MA0903.1_HOXB3	HOXB3	1.513027521	0.244708767	1.834327278	0.076234414	0.229894404
MA0798.1_RFX3	RFX3	1.493908093	0.469845676	2.03748818	0.082271083	0.246175488
MA0502.1_NFYB	NFYB	1.443468673	0.07659961	1.564027529	0.100025544	0.296998924
MA0737.1_GLIS3	GLIS3	1.424309965	0.305103048	1.884028472	0.107501382	0.316759797
MA0091.1_TAL1::TCF3	TAL1::TCF3	1.416678445	0.10641364	1.537678056	0.110596197	0.323410091
MA0649.1_HEY2	HEY2	1.409360709	0.000793822	1.427727913	0.113627396	0.329775752
MA0773.1_MEF2D	MEF2D	1.407308441	0.185837359	1.714861953	0.114488775	0.329796025
MA0733.1_EGR4	EGR4	1.400817244	0.239208757	1.637039072	0.117246021	0.335236771
MA0794.1_PROX1	PROX1	1.390553403	0.059271126	1.437446002	0.121708188	0.345436476
MA0822.1_HES7	HES7	1.377991618	0.071867125	1.685067646	0.127342314	0.358789292
MA0052.3_MEF2A	MEF2A	1.373428408	0.143452085	1.650511534	0.129436634	0.362047396
MA0779.1_PAX1	PAX1	1.359975178	0.410126327	1.755130548	0.135760673	0.377004458
MA0768.1_LEF1	LEF1	1.351506724	0.11832921	1.617946683	0.139857156	0.38560616
MA0017.2_NR2F1	NR2F1	1.347807404	0.440748938	1.836331356	0.141674939	0.387847705
MA0628.1_POU6F1	POU6F1	1.336071563	0.420048745	1.753025226	0.147556471	0.401104211
MA0906.1_HOXC12	HOXC12	1.320733518	0.036427971	1.453338602	0.155508742	0.419764856
MA0748.1_YY2	YY2	1.302287122	0.171630234	1.464664357	0.165475837	0.442024557
MA0889.1_GBX1	GBX1	1.299108712	0.15776162	1.804774557	0.167238081	0.442024557
MA0803.1_TBX15	TBX15	1.297005526	0.178289718	1.593858952	0.168411462	0.442024557
MA0030.1_FOXF2	FOXF2	1.295804239	0.147862159	1.497294356	0.169084275	0.442024557
MA0839.1_CREB3L1	CREB3L1	1.295097627	0.005849593	1.544817246	0.169480918	0.442024557
MA0844.1_XBP1	XBP1	1.286993651	0.196268392	1.674807594	0.174076943	0.45096443
MA0876.1_BSX	BSX	1.278398866	0.382031345	1.506112168	0.179046099	0.46017032
MA0865.1_E2F8	E2F8	1.276742795	0.048248496	1.486440128	0.180014814	0.46017032
MA0079.3_SP1	SP1	1.26926453	0.012619692	1.496685987	0.184434554	0.463586307
MA0756.1_ONECUT2	ONECUT2	1.265699663	0.070864753	1.566300759	0.186567603	0.463586307
MA0528.1_ZNF263	ZNF263	1.265250297	0.014012887	1.359970594	0.186837683	0.463586307
MA0900.1_HOXA2	HOXA2	1.264500336	0.187989072	1.570481855	0.187289028	0.463586307
MA0635.1_BARHL2	BARHL2	1.262773148	0.048508708	1.504110223	0.188331341	0.463586307
MA0084.1_SRY	SRY	1.262399875	0.142840654	1.616693238	0.188557125	0.463586307
MA0513.1_SMAD2::SMAD3::SMAD4	SMAD2::SMAD3::SMAD4	1.24900782	0.069634935	1.49609844	0.196780629	0.479022306
MA0161.1_NFIC	NFIC	1.247032382	0.165090232	1.515993046	0.19801394	0.479022306
MA0813.1_TFAP2B(var.3)	TFAP2B(var.3)	1.24616284	0.271260775	1.648839202	0.198558469	0.479022306
MA0744.1_SCRT2	SCRT2	1.228089299	0.002618433	1.464431556	0.21010561	0.502710556
MA0672.1_NKX2-3	NKX2-3	1.226744687	0.067382588	1.333286731	0.210982151	0.502710556
MA0162.2_EGR1	EGR1	1.224712721	0.169377892	1.556785767	0.212311361	0.502774144
MA0479.1_FOXH1	FOXH1	1.213908637	0.021185816	1.253854827	0.219471602	0.51656121
MA0791.1_POU4F3	POU4F3	1.206202197	0.092214625	1.308264193	0.224674227	0.523881353
MA0764.1_ETV4	ETV4	1.203655288	0.071961513	1.508831912	0.226411069	0.523881353
MA0523.1_TCF7L2	TCF7L2	1.203301038	0.020132758	1.408284338	0.226653331	0.523881353
MA0051.1_IRF2	IRF2	1.198621716	0.14859947	1.431470002	0.229869086	0.528151591
MA0892.1_GSX1	GSX1	1.190631831	0.33470314	1.556716642	0.235427259	0.533632674
MA0156.2_FEV	FEV	1.188180042	0.230061639	1.654353421	0.237149839	0.533632674
MA0475.2_FLI1	FLI1	1.187642276	0.322939086	1.526292736	0.237528728	0.533632674
MA0886.1_EMX2	EMX2	1.18561108	0.366599277	1.606208993	0.238963286	0.533632674
MA0058.3_MAX	MAX	1.1853233	0.28077314	1.47838602	0.239166976	0.533632674
MA0805.1_TBX1	TBX1	1.18298274	0.084465501	1.414606536	0.240827686	0.534249924
MA0648.1_GSC	GSC	1.175629814	0.168572212	1.419027579	0.246091894	0.542808406
MA0694.1_ZBTB7B	ZBTB7B	1.17251316	0.160156672	1.467921882	0.248344699	0.544665079
MA0593.1_FOXP2	FOXP2	1.16659635	0.181436032	1.40577419	0.252656611	0.548368506
MA0873.1_HOXD12	HOXD12	1.165360823	0.01126845	1.478615751	0.253562795	0.548368506
MA0868.1_SOX8	SOX8	1.164364161	0.386918803	1.372384018	0.25429524	0.548368506
MA0901.1_HOXB13	HOXB13	1.142011921	0.291363665	1.524451553	0.27106066	0.578979149
MA0738.1_HIC2	HIC2	1.141450274	0.0049578	1.166525745	0.271490223	0.578979149
MA0700.1_LHX2	LHX2	1.139470063	0.081530523	1.573248985	0.273007959	0.579016881
MA0673.1_NKX2-8	NKX2-8	1.129919916	0.123195135	1.346946523	0.280397792	0.591440152
MA0059.1_MAX::MYC	MAX::MYC	1.125647282	0.370297854	1.531505334	0.283741302	0.595239905
MA0767.1_GCM2	GCM2	1.112575163	0.08335472	1.397027304	0.294112663	0.609640507
MA0070.1_PBX1	PBX1	1.111623247	0.153725252	1.360544723	0.294876195	0.609640507
MA0894.1_HESX1	HESX1	1.11104074	0.095871892	1.553941623	0.295343976	0.609640507
MA0893.1_GSX2	GSX2	1.097690764	0.1469186	1.404985244	0.306178425	0.628642937
MA0595.1_SREBF1	SREBF1	1.093045898	0.35889993	1.451698719	0.309998694	0.633119027
MA0774.1_MEIS2	MEIS2	1.087959381	0.332767464	1.436062141	0.314211804	0.638346086

MA0665.1_MSC	MSC	1.085549537	0.02223647	1.298624374	0.316218576	0.63905953
MA0786.1_POU3F1	POU3F1	1.077504855	0.055486483	1.30882108	0.322967124	0.648508954
MA0698.1_ZBTB18	ZBTB18	1.075980391	0.262810322	1.372361126	0.324254477	0.648508954
MA0024.3_E2F1	E2F1	1.073821825	0.06233017	1.301614896	0.326081905	0.648802141
MA0688.1_TBX2	TBX2	1.059476447	0.109199769	1.277962056	0.338361793	0.66816237
MA0138.2_REST	REST	1.057030828	0.027321525	1.25698148	0.340478397	0.66816237
MA0638.1_CREB3	CREB3	1.056423388	0.344468904	1.259318772	0.341005147	0.66816237
MA0812.1_TFAP2B(var.2)	TFAP2B(var.2)	1.027511277	0.106070258	1.39830447	0.366537516	0.713644961
MA0511.2_RUNX2	RUNX2	1.024882962	0.147567634	1.249140323	0.368902019	0.713644961
MA0828.1_SREBF2(var.2)	SREBF2(var.2)	1.023874754	0.130080047	1.24724317	0.369810888	0.713644961
MA0498.2_MEIS1	MEIS1	1.021878969	0.170330038	1.209557559	0.371613049	0.713644961
MA0914.1_ISL2	ISL2	1.018230086	0.128504322	1.232927185	0.374918231	0.716427906
MA0802.1_TBR1	TBR1	1.014409567	0.280454413	1.221808739	0.378393001	0.719505904
MA0071.1_RORA	RORA	1.01104808	0.009363743	1.242458447	0.381462076	0.721786085
MA0154.3_EBF1	EBF1	1.006464782	0.06295864	1.220074505	0.385664219	0.72309736
MA0072.1_RORA(var.2)	RORA(var.2)	1.003145157	0.194369633	1.223388251	0.388720243	0.72309736
MA0474.2_ERG	ERG	1.003063114	0.360754856	1.355231591	0.388795902	0.72309736
MA0732.1_EGR3	EGR3	1.000288228	0.146581442	1.293037969	0.39135858	0.72309736
MA0758.1_E2F7	E2F7	1.000111941	0.098553055	1.157991297	0.391521627	0.72309736
MA0815.1_TFAP2C(var.3)	TFAP2C(var.3)	0.992196417	0.279386998	1.365324006	0.398872002	0.732852069
MA0872.1_TFAP2A(var.3)	TFAP2A(var.3)	0.990343839	0.287749783	1.325985674	0.400600484	0.732852069
MA0784.1_POU1F1	POU1F1	0.987461185	0.132125116	1.371429902	0.403296097	0.734303271
MA0661.1_MEOX1	MEOX1	0.971005644	0.016661882	1.144102572	0.418820658	0.758049713
MA0131.2_HINFP	HINFP	0.969485768	0.03046079	1.183321956	0.420265903	0.758049713
MA0060.2_NFYA	NFYA	0.967280804	0.167461711	1.199321379	0.422365895	0.758294118
MA0777.1_MYBL2	MYBL2	0.956528726	0.237231575	1.283589874	0.43266064	0.771103028
MA0752.1_ZNF410	ZNF410	0.955660544	0.265894956	1.237830707	0.433495744	0.771103028
MA0148.3_FOXA1	FOXA1	0.952641901	0.099047333	1.176218925	0.436403727	0.772714855
MA0711.1_OTX1	OTX1	0.949847877	0.161352431	1.215498928	0.439101267	0.773941047
MA0706.1_MEOX2	MEOX2	0.941138587	0.323643577	1.113202374	0.447545378	0.782734896
MA0469.2_E2F3	E2F3	0.938406857	0.02479194	1.134751088	0.450204689	0.782734896
MA0887.1_EVX1	EVX1	0.938084707	0.056450564	1.103434106	0.450518628	0.782734896
MA0650.1_HOXA13	HOXA13	0.936358751	0.011775748	1.097605526	0.452201766	0.782734896
MA0014.2_PAX5	PAX5	0.916715773	0.001795899	1.067572192	0.471489292	0.806021491
MA0641.1_ELF4	ELF4	0.91435048	0.053696462	1.056326475	0.473827135	0.806021491
MA0806.1_TBX4	TBX4	0.914040571	0.109756154	1.096569747	0.474133677	0.806021491
MA0807.1_TBX5	TBX5	0.914040571	0.109756154	1.096569747	0.474133677	0.806021491
MA0130.1_ZNF354C	ZNF354C	0.912058354	0.274665934	1.098320104	0.476095596	0.806021491
MA0103.2_ZEB1	ZEB1	0.895350565	0.113524366	1.108336524	0.492712378	0.827656392
MA0799.1_RFX4	RFX4	0.891473965	0.0822678	1.002683789	0.496586774	0.827656392
MA0712.1_OTX2	OTX2	0.889675302	0.187549772	1.114073631	0.498386633	0.827656392
MA0101.1_REL	REL	0.888488094	0.1798241	1.051828326	0.49957538	0.827656392
MA0690.1_TBX21	TBX21	0.888467807	0.260117605	1.02284299	0.499595698	0.827656392
MA0891.1_GSC2	GSC2	0.882001333	0.032257354	1.058698535	0.506080641	0.834816784
MA0729.1_RARA	RARA	0.877244147	0.220488598	1.14561295	0.510861641	0.839117419
MA0895.1_HMBOX1	HMBOX1	0.865705522	0.18772159	1.038688229	0.522490122	0.854581301
MA0800.1_EOMES	EOMES	0.862480974	0.208916121	1.046160073	0.525747044	0.856279996
MA0750.1_ZBTB7A	ZBTB7A	0.855020546	0.033890936	1.046843178	0.533292868	0.864920366
MA0646.1_GCM1	GCM1	0.849671006	0.103951022	1.027382252	0.538711616	0.870053071
MA0792.1_POU5F1B	POU5F1B	0.846785376	0.026350285	1.036790986	0.541636985	0.871132818
MA0780.1_PAX3	PAX3	0.834662958	0.006643015	0.994319146	0.553941165	0.879986331
MA0076.2_ELK4	ELK4	0.831664354	0.139717722	1.086953797	0.556987621	0.879986331
MA0115.1_NR1H2::RXRA	NR1H2::RXRA	0.82828932	0.159489505	1.010237176	0.560417491	0.879986331
MA0730.1_RARA(var.2)	RARA(var.2)	0.823164858	0.019680502	0.897190826	0.565626691	0.879986331
MA0080.4_SPI1	SPI1	0.821850039	0.034865599	1.004678479	0.566963456	0.879986331
MA0506.1_NRF1	NRF1	0.820435804	0.011000752	0.995809372	0.568401359	0.879986331
MA0821.1_HES5	HES5	0.817416462	0.086791588	0.993510539	0.571471368	0.879986331
MA0890.1_GBX2	GBX2	0.816896385	0.077222831	1.074579534	0.572000184	0.879986331
MA0598.2_EHF	EHF	0.816842286	0.059821889	1.099992413	0.572055191	0.879986331
MA0524.2_TFAP2C	TFAP2C	0.813814516	0.204901281	1.11368267	0.575133812	0.879986331
MA0484.1_HNF4G	HNF4G	0.811511063	0.052379354	0.927717465	0.577475861	0.879986331
MA0866.1_SOX21	SOX21	0.806932166	0.148823953	0.970743395	0.582130924	0.879986331
MA0845.1_FOXB1	FOXB1	0.806153431	0.139179166	1.126034044	0.582922507	0.879986331
MA0517.1_STAT1::STAT2	STAT1::STAT2	0.798770445	0.106129731	1.030150504	0.590424974	0.879986331
MA0724.1_VENTX	VENTX	0.798534329	0.273597149	1.066566458	0.590664826	0.879986331
MA0018.2_CREB1	CREB1	0.794918537	0.165533477	1.054715978	0.594337013	0.879986331
MA0699.1_LBX2	LBX2	0.794609878	0.114636295	0.957351556	0.59465041	0.879986331
MA0826.1_OLIG1	OLIG1	0.793525214	0.032669736	0.975522999	0.595751619	0.879986331
MA0817.1_BHLHE23	BHLHE23	0.789764492	0.084991952	1.012186244	0.599568361	0.879986331
MA0785.1_POU2F1	POU2F1	0.784382529	0.16608468	0.895187186	0.605026339	0.879986331
MA0618.1_LBX1	LBX1	0.780761614	0.134005634	0.944154608	0.608695208	0.879986331
MA0654.1_ISX	ISX	0.780761614	0.134005634	0.944154608	0.608695208	0.879986331
MA0675.1_NKX6-2	NKX6-2	0.780761614	0.134005634	0.944154608	0.608695208	0.879986331
MA0701.1_LHX9	LHX9	0.780761614	0.134005634	0.944154608	0.608695208	0.879986331
MA0716.1_PRRX1	PRRX1	0.780761614	0.134005634	0.944154608	0.608695208	0.879986331
MA0717.1_RAX2	RAX2	0.780761614	0.134005634	0.944154608	0.608695208	0.879986331
MA0882.1_DLX6	DLX6	0.780761614	0.134005634	0.944154608	0.608695208	0.879986331
MA0743.1_SCRT1	SCRT1	0.775695135	0.050757368	0.927359621	0.61382383	0.884089546
MA0069.1_Pax6	Pax6	0.760660026	0.134935613	0.930118501	0.629001752	0.894918848
MA0003.3_TFAP2A	TFAP2A	0.758101589	0.097660275	0.89552114	0.631577215	0.894918848
MA0753.1_ZNF740	ZNF740	0.758054102	0.02048379	0.922158842	0.631624996	0.894918848
MA0152.1_NFATC2	NFATC2	0.75676594	0.163714917	0.976671694	0.632920807	0.894918848
MA0471.1_E2F6	E2F6	0.756751993	0.047558994	0.925351906	0.632934833	0.894918848
MA0640.1_ELF3	ELF3	0.751084756	0.026903426	0.894581602	0.638628124	0.898330231
MA0670.1_NFIA	NFIA	0.749715134	0.23844105	0.864661089	0.640002107	0.898330231
MA0687.1_SPIC	SPIC	0.746475828	0.159717676	0.975032436	0.643248563	0.899615743
MA0827.1_OLIG3	OLIG3	0.742257	0.08406405	0.869109838	0.647469739	0.902250251
MA0689.1_TBX20	TBX20	0.735210887	0.204698119	0.874768838	0.654500981	0.90580949
MA0159.1_RARA::RXRA	RARA::RXRA	0.734993795	0.029707795	0.992531	0.654717222	0.90580949
MA0098.3_ETS1	ETS1	0.732295031	0.021581817	0.906704188	0.657403364	0.906277494
MA0158.1_HOXA5	HOXA5	0.72752236	0.237877332	0.864802324	0.662144162	0.907529625
MA0789.1_POU3F4	POU3F4	0.726645423	0.229384367	0.86336161	0.663013871	0.907529625
MA0793.1_POU6F2	POU6F2	0.71766611	0.030103369	0.839317177	0.671893293	0.910272219
MA0083.3_SRF	SRF	0.717137716	0.237210012	0.837870435	0.672414283	0.910272219
MA0637.1_CENPB	CENPB	0.716640579	0.06660836	1.010254135	0.672904296	0.910272219
MA0149.1_EWSR1-FLI1	EWSR1-FLI1	0.715070949	0.054106366	0.866850861	0.674450401	0.910272219
MA0596.1_SREBF2	SREBF2	0.700584261	0.027072152	0.962791105	0.688642154	0.914003832
MA0801.1_MGA	MGA	0.696080604	0.171629073	0.788929756	0.693023646	0.914003832

MA0042.2_FOXI1	FOXI1	0.695290774	0.032750594	0.811517673	0.693790473	0.914003832
MA0847.1_FOXD2	FOXD2	0.695290774	0.032750594	0.811517673	0.693790473	0.914003832
MA0848.1_FOXO4	FOXO4	0.695290774	0.032750594	0.811517673	0.693790473	0.914003832
MA0849.1_FOXO6	FOXO6	0.695290774	0.032750594	0.811517673	0.693790473	0.914003832
MA0850.1_FOXP3	FOXP3	0.695290774	0.032750594	0.811517673	0.693790473	0.914003832
MA0442.1_SOX10	SOX10	0.680219661	0.161871792	0.902750216	0.708327367	0.929980829
MA0684.1_RUNX3	RUNX3	0.675589003	0.240511143	0.792667831	0.712755556	0.931129857
MA0504.1_NR2C2	NR2C2	0.674255707	0.023067467	0.928689268	0.714027041	0.931129857
MA0772.1_IRF7	IRF7	0.664473157	0.012599633	0.785994481	0.723306237	0.937506854
MA0727.1_NR3C2	NR3C2	0.662131032	0.059834523	0.798179544	0.725514443	0.937506854
MA0795.1_SMAD3	SMAD3	0.658743076	0.074631383	0.802107488	0.728699207	0.937506854
MA0796.1_TGIF1	TGIF1	0.652831206	0.020089094	0.796431895	0.734229034	0.937506854
MA0599.1_KLF5	KLF5	0.652499397	0.055491381	0.7829052	0.734538345	0.937506854
MA0696.1_ZIC1	ZIC1	0.651637587	0.118440756	0.834865623	0.735341189	0.937506854
MA0074.1_RXRA::VDR	RXRA::VDR	0.651017263	0.074720793	0.79717313	0.735918593	0.937506854
MA0095.2_YY1	YY1	0.645864075	0.129823594	0.765852647	0.740699609	0.940030955
MA0776.1_MYBL1	MYBL1	0.643622373	0.119995866	0.816888042	0.742770573	0.940030955
MA0516.1_SP2	SP2	0.633497986	0.004051617	0.647503386	0.752054914	0.947057634
MA0486.2_HSF1	HSF1	0.632207766	0.048653094	0.715569092	0.753229776	0.947057634
MA0770.1_HSF2	HSF2	0.627170584	0.01503041	0.744716852	0.757798101	0.947515778
MA0810.1_TFAP2A(var.2)	TFAP2A(var.2)	0.626389779	0.065856768	0.728826055	0.758503563	0.947515778
MA0762.1_ETV2	ETV2	0.6140938	0.044248269	0.747267844	0.769515919	0.955433592
MA0811.1_TFAP2B	TFAP2B	0.602175726	0.197181486	0.711818001	0.780008692	0.955433592
MA0867.1_SOX4	SOX4	0.600073463	0.072863687	0.771334253	0.781840325	0.955433592
MA0473.2_ELF1	ELF1	0.599637384	0.040369431	0.750588916	0.78221953	0.955433592
MA0788.1_POU3F3	POU3F3	0.598585255	0.131293635	0.687132933	0.783133393	0.955433592
MA0741.1_KLF16	KLF16	0.597303303	0.121297488	0.778578017	0.784244869	0.955433592
MA0804.1_TBX19	TBX19	0.594874815	0.062753251	0.722491895	0.78634433	0.955433592
MA0787.1_POU3F2	POU3F2	0.593630524	0.078125177	0.720929003	0.787416932	0.955433592
MA0145.3_TFCP2	TFCP2	0.593543488	0.092069199	0.724861123	0.787491879	0.955433592
MA0107.1_RELA	RELA	0.589596964	0.096805342	0.717230503	0.790879323	0.955433592
MA0683.1_POU4F2	POU4F2	0.588204593	0.114618153	0.813314583	0.792069299	0.955433592
MA0651.1_HOXC11	HOXC11	0.581889645	0.160338622	0.697876007	0.797432122	0.958905916
MA0009.2_T	T	0.566684951	0.104349334	0.677423771	0.810108167	0.971123455
MA0510.2_RFX5	RFX5	0.561446925	0.176169462	0.767111886	0.814395398	0.973240321
MA0680.1_PAX7	PAX7	0.556793119	0.187757874	0.656196173	0.818169273	0.973848113
MA0645.1_ETV6	ETV6	0.549716058	0.083744359	0.667848358	0.823843668	0.973848113
MA0679.1_ONECUT1	ONECUT1	0.547999981	0.027223471	0.593040511	0.825207732	0.973848113
MA0735.1_GLIS1	GLIS1	0.537482097	0.076029631	0.679345447	0.833464893	0.973848113
MA0033.2_FOXL1	FOXL1	0.535555403	0.086016054	0.645772042	0.834957979	0.973848113
MA0671.1_NFIX	NFIX	0.522774492	0.024339759	0.586024197	0.844706446	0.973848113
MA0771.1_HSF4	HSF4	0.515362248	0.000868308	0.608484699	0.850233535	0.973848113
MA0686.1_SPDEF	SPDEF	0.515027752	0.008558812	0.572284627	0.850480734	0.973848113
MA0258.2_ESR2	ESR2	0.513339225	0.028301369	0.621903738	0.851725635	0.973848113
MA0468.1_DUX4	DUX4	0.511787174	0.009274908	0.626289971	0.852865557	0.973848113
MA0056.1_MZF1	MZF1	0.511478035	0.082542658	0.668465497	0.853092107	0.973848113
MA0715.1_PROP1	PROP1	0.51100778	0.126741656	0.620468171	0.853436412	0.973848113
MA0066.1_PPARG	PPARG	0.51051527	0.049115548	0.623049295	0.853796599	0.973848113
MA0136.2_ELF5	ELF5	0.502163947	0.03645902	0.60227186	0.8598394	0.973848113
MA0909.1_HOXD13	HOXD13	0.501112336	0.035475482	0.595996943	0.860591592	0.973848113
MA0725.1_VSX1	VSX1	0.500253508	0.074164151	0.604226395	0.861204434	0.973848113
MA0726.1_VSX2	VSX2	0.500253508	0.074164151	0.604226395	0.861204434	0.973848113
MA0612.1_EMX1	EMX1	0.498715667	0.096436321	0.679685487	0.862298522	0.973848113
MA0508.1_PRDM1	PRDM1	0.496435865	0.066745948	0.589030768	0.863912702	0.973848113
MA0814.1_TFAP2C(var.2)	TFAP2C(var.2)	0.484147214	0.014022766	0.51466875	0.872452062	0.973848113
MA0757.1_ONECUT3	ONECUT3	0.481585485	0.057373247	0.620965385	0.874197556	0.973848113
MA0834.1_ATF7	ATF7	0.480634993	0.077469226	0.583255278	0.874842129	0.973848113
MA0465.1_CDX2	CDX2	0.479754566	0.050619935	0.566324096	0.875437703	0.973848113
MA0113.3_NR3C1	NR3C1	0.478181466	0.117453379	0.656212998	0.876498284	0.973848113
MA0781.1_PAX9	PAX9	0.475976663	0.099105725	0.573258332	0.877977055	0.973848113
MA0819.1_CLOCK	CLOCK	0.45886127	0.04278572	0.54381498	0.889147581	0.98121732
MA0657.1_KLF13	KLF13	0.452913197	0.094299942	0.630184338	0.892900072	0.98121732
MA0625.1_NFATC3	NFATC3	0.452496625	0.049474423	0.623415157	0.893160348	0.98121732
MA0488.1_JUN	JUN	0.436949877	0.019206793	0.508429822	0.902635502	0.98121732
MA0846.1_FOXC2	FOXC2	0.426135264	0.036920675	0.515688057	0.90895027	0.98121732
MA0736.1_GLIS2	GLIS2	0.425909973	0.052442142	0.512620253	0.909079391	0.98121732
MA0678.1_OLIG2	OLIG2	0.422519227	0.071587164	0.511121865	0.911010718	0.98121732
MA0864.1_E2F2	E2F2	0.419138833	0.013295137	0.51142472	0.912913687	0.98121732
MA0112.3_ESR1	ESR1	0.414938944	0.097448705	0.504820799	0.915246675	0.98121732
MA0907.1_HOXC13	HOXC13	0.40999789	0.096243468	0.507653398	0.917946853	0.98121732
MA0691.1_TFAP4	TFAP4	0.40706703	0.002019662	0.471535768	0.919525718	0.98121732
MA0613.1_FOXG1	FOXG1	0.39277738	0.03772665	0.552848637	0.92697991	0.98121732
MA0157.2_FOXO3	FOXO3	0.39277738	0.03772665	0.552848637	0.92697991	0.98121732
MA0032.2_FOXC1	FOXC1	0.392402457	0.097240873	0.450930828	0.927170034	0.98121732
MA0856.1_RXRG	RXRG	0.388185028	0.076049239	0.539913284	0.929289464	0.98121732
MA0746.1_SP3	SP3	0.370894008	0.0330171	0.502222953	0.937609347	0.98121732
MA0749.1_ZBED1	ZBED1	0.37007546	0.049975789	0.447745879	0.937988479	0.98121732
MA0751.1_ZIC4	ZIC4	0.365217441	0.111320434	0.435333028	0.94021123	0.98121732
MA0790.1_POU4F1	POU4F1	0.364115456	0.028751583	0.439290277	0.940708921	0.98121732
MA0908.1_HOXD11	HOXD11	0.357015592	0.045597318	0.431358274	0.943857753	0.98121732
MA0695.1_ZBTB7C	ZBTB7C	0.351007268	0.007951209	0.363739346	0.946444595	0.98121732
MA0656.1_JDP2(var.2)	JDP2(var.2)	0.345124502	0.090968414	0.410020852	0.948908398	0.98121732
MA0760.1_ERF	ERF	0.344060374	0.046999255	0.386050172	0.9493468	0.98121732
MA0031.1_FOXD1	FOXD1	0.331109933	0.00942845	0.350845464	0.954504348	0.98121732
MA0674.1_NKX6-1	NKX6-1	0.327819846	0.023256013	0.39867762	0.955762509	0.98121732
MA0132.2_PDX1	PDX1	0.327819846	0.023256013	0.39867762	0.955762509	0.98121732
MA0723.1_VAX2	VAX2	0.327819846	0.023256013	0.39867762	0.955762509	0.98121732
MA0878.1_CDX1	CDX1	0.312202643	0.079549842	0.37842163	0.961449302	0.98121732
MA0899.1_HOXA10	HOXA10	0.309231698	0.033315776	0.378186405	0.962478158	0.98121732
MA0492.1_JUND(var.2)	JUND(var.2)	0.306336548	0.033009066	0.369794937	0.963464607	0.98121732
MA0835.1_BATF3	BATF3	0.303067084	0.021202081	0.360745512	0.964559491	0.98121732
MA0884.1_DUXA	DUXA	0.298778009	0.02295291	0.349987458	0.965965237	0.98121732
MA0905.1_HOXC10	HOXC10	0.266979823	0.047357559	0.365957346	0.975325601	0.988125149
MA0685.1_SP4	SP4	0.255696752	0.000274002	0.30346481	0.978209617	0.98845265
MA0507.1_POU2F2	POU2F2	0.22934576	0.06292825	0.276405472	0.984095997	0.989724092
MA0722.1_VAX1	VAX1	0.226839614	0.061710089	0.300232207	0.984595988	0.989724092
MA0713.1_PHOX2A	PHOX2A	0.189906351	0.039039165	0.231457686	0.990836393	0.993409994
MA0747.1_SP8	SP8	0.151217088	0.017532201	0.179599177	0.995318521	0.995318521

Supplementary Table 2

motifID	name	variability	bootstrap_lower_bound	bootstrap_upper_bound	p_value	p_value_adj
MA0861.1_TP73	TP73	18.13308758	2.731799037	19.92219115	1.58E-213	6.11E-211
MA0106.3_TP53	TP53	15.23643808	0.732869497	16.64912957	1.24E-150	2.39E-148
MA0525.2_TP63	TP63	15.00820154	2.101934961	16.22211041	3.83E-146	4.92E-144
MA0077.1_SOX9	SOX9	4.566939186	0.411254682	4.896853808	1.66E-13	1.60E-11
MA0102.3_CEBPA	CEBPA	4.029686206	0.455172416	4.737191825	1.50E-10	1.16E-08
MA0161.1_NFIC	NFIC	3.906329949	0.737335588	4.299763749	6.32E-10	4.07E-08
MA0639.1_DBP	DBP	3.419250293	0.106736648	3.988904421	1.18E-07	6.48E-06
MA0706.1_MEOX2	MEOX2	3.376559698	0.024962136	3.896974696	1.79E-07	8.66E-06
MA0025.1_NFIL3	NFIL3	3.317932005	0.197808646	4.004664549	3.18E-07	1.36E-05
MA0489.1_JUN(var.2)	JUN(var.2)	3.305982064	0.212673503	3.6357347	3.57E-07	1.38E-05
MA0043.2_HLF	HLF	3.287626413	0.087628486	3.850422698	4.26E-07	1.49E-05
MA0139.1_CTCF	CTCF	3.262531947	0.041221975	3.497796226	5.41E-07	1.74E-05
MA0841.1_NFE2	NFE2	3.240822694	0.083302551	3.658110459	6.64E-07	1.97E-05
MA0843.1_TEF	TEF	3.152551201	0.213711491	3.839085757	1.51E-06	4.16E-05
MA0798.1_RFX3	RFX3	3.128974719	0.254149015	3.698676109	1.87E-06	4.81E-05
MA0478.1_FOSL2	FOSL2	3.067936852	0.149315695	3.421916984	3.24E-06	7.81E-05
MA0119.1_NFIC::TLX1	NFIC::TLX1	2.972181124	0.817413224	3.390412653	7.49E-06	0.000170026
MA0655.1_JDP2	JDP2	2.964041947	0.188388198	3.468150479	8.03E-06	0.00017221
MA0510.2_RFX5	RFX5	2.885974674	0.388956726	3.382556895	1.55E-05	0.000315724
MA0908.1_HOXD11	HOXD11	2.871656543	0.348286657	3.249055741	1.75E-05	0.000337855
MA0477.1_FOSL1	FOSL1	2.848019047	0.159467805	3.09791457	2.13E-05	0.0003911
MA0476.1_FOS	FOS	2.840357967	0.220465838	3.23243881	2.27E-05	0.000395052
MA0491.1_JUND	JUND	2.835698317	0.095326392	3.28963027	2.35E-05	0.000395052
MA0797.1_TGIF2	TGIF2	2.817681519	0.28151757	3.145272479	2.73E-05	0.000438492
MA0517.1_STAT1::STAT2	STAT1::STAT2	2.807981289	0.034585969	2.931540579	2.95E-05	0.000455405
MA0727.1_NR3C2	NR3C2	2.798941201	0.162127553	3.093433553	3.17E-05	0.000471074
MA0470.1_E2F4	E2F4	2.762853428	0.277785301	3.299014328	4.24E-05	0.000605687
MA0838.1_CEBPG	CEBPG	2.717306919	0.252286904	3.204654562	6.07E-05	0.000836382
MA0770.1_HSF2	HSF2	2.67557474	0.263473853	3.271889119	8.38E-05	0.001115834
MA0490.1_JUNB	JUNB	2.659734364	0.09256919	2.828658348	9.46E-05	0.001217772
MA0799.1_RFX4	RFX4	2.618881379	0.077291526	3.025495774	0.000128951	0.00160565
MA0773.1_MEF2D	MEF2D	2.578806929	0.203906173	3.151866477	0.00017378	0.00209622
MA0113.3_NR3C1	NR3C1	2.562875958	0.325307474	2.820691769	0.000195392	0.002285493
MA0105.4_NFKB1	NFKB1	2.531441086	0.284354436	3.249749037	0.000245668	0.002789054
MA0775.1_MEIS3	MEIS3	2.514511049	0.361526747	2.863224796	0.000277557	0.003061052
MA0017.2_NR2F1	NR2F1	2.504960676	0.24665256	3.032699612	0.000297221	0.00318687
MA0466.2_CEBPB	CEBPB	2.490030279	0.418683997	2.882576458	0.000330603	0.003358232
MA0837.1_CEBPE	CEBPE	2.490030279	0.418683997	2.882576458	0.000330603	0.003358232
MA0836.1_CEBPD	CEBPD	2.482743352	0.24395083	2.985472689	0.000348144	0.003445733
MA0796.1_TGIF1	TGIF1	2.453083871	0.169287571	2.626890203	0.000428945	0.004139316
MA0600.2_RFX2	RFX2	2.393882087	0.342707352	2.759467962	0.000645297	0.006071706
MA0873.1_HOXD12	HOXD12	2.389414588	0.62706081	2.746394479	0.000665198	0.006071706
MA0527.1_ZBTB33	ZBTB33	2.386532759	0.397235037	2.674646357	0.000678337	0.006071706
MA0052.3_MEF2A	MEF2A	2.383566775	0.306640083	2.911628028	0.000692112	0.006071706
MA0651.1_HOXC11	HOXC11	2.36985292	0.403138205	2.653536279	0.000759258	0.006474499
MA0810.1_TFAP2A(var.2)	TFAP2A(var.2)	2.36746121	0.407755735	2.802739233	0.000771572	0.006474499
MA0795.1_SMAD3	SMAD3	2.359643538	0.726452849	2.738800911	0.000813132	0.006678061
MA0524.2_TFAP2C	TFAP2C	2.330715389	0.574774787	2.655929421	0.000985708	0.007926732
MA0737.1_GLIS3	GLIS3	2.326934098	0.209494367	2.756035925	0.001010625	0.007961248
MA0778.1_NFKB2	NFKB2	2.284820167	0.118893464	2.765497992	0.001330533	0.010271711
MA0660.1_MEF2B	MEF2B	2.264876977	0.236303204	2.744270851	0.001512663	0.011448782
MA0710.1_NOTO	NOTO	2.242902767	0.14399508	2.64006206	0.001739825	0.012914854
MA0808.1_TEAD3	TEAD3	2.236735866	0.293774505	2.602775797	0.001809006	0.013175027
MA0844.1_XBP1	XBP1	2.204453398	0.595842562	2.658980333	0.002214331	0.015828367
MA0887.1_EVX1	EVX1	2.189017782	0.680824894	2.626755789	0.002436252	0.017098058
MA0502.1_NFYB	NFYB	2.184225246	0.396455504	2.518943435	0.002509197	0.01729554
MA0811.1_TFAP2B	TFAP2B	2.172210782	0.526599389	2.577603466	0.002700954	0.018290671
MA0777.1_MYBL2	MYBL2	2.151812633	0.505977799	2.554957398	0.003057491	0.02027478
MA0771.1_HSF4	HSF4	2.149581413	0.352627804	2.515509397	0.003098995	0.02027478
MA0486.2_HSF1	HSF1	2.122323976	0.120294313	2.561791855	0.003649267	0.023139327
MA0079.3_SP1	SP1	2.121980667	0.079825091	2.19080999	0.003656733	0.023139327
MA0465.1_CDX2	CDX2	2.113106677	0.062012304	2.541261058	0.003854605	0.023998022
MA0814.1_TFAP2C(var.2)	TFAP2C(var.2)	2.077052681	0.159791653	2.524525545	0.004762742	0.029181247
MA0671.1_NFIX	NFIX	2.073406173	0.162124121	2.470133568	0.004864637	0.029339845
MA0653.1_IRF9	IRF9	2.068683683	0.530167733	2.340691784	0.004999534	0.029689539
MA0158.1_HOXA5	HOXA5	2.064930432	0.262479676	2.402323689	0.005109151	0.02988079
MA0612.1_EMX1	EMX1	2.055571956	0.481015876	2.300832599	0.005392011	0.031064424
MA0099.2_FOS::JUN	FOS::JUN	2.045705953	0.131387141	2.204226615	0.005705479	0.032386981
MA0462.1_BATF::JUN	BATF::JUN	2.038069155	0.165618187	2.40694525	0.00595932	0.033270486
MA0050.2_IRF1	IRF1	2.034116759	0.588161731	2.388039847	0.006094657	0.033270486
MA0070.1_PBX1	PBX1	2.033032582	0.058847032	2.403439956	0.006132263	0.033270486
MA0107.1_RELA	RELA	2.030927052	0.152604169	2.43413033	0.006205894	0.033270486
MA0809.1_TEAD4	TEAD4	2.019037174	0.036869927	2.191139515	0.006636845	0.035093454
MA0090.2_TEAD1	TEAD1	2.005886054	0.045547942	2.082962129	0.007144733	0.037268475
MA0886.1_EMX2	EMX2	1.996756273	0.218435005	2.365786158	0.007517586	0.038690512
MA0481.1_FOXP1	FOXP1	1.993605408	0.216602653	2.437848973	0.007650264	0.038855286
MA0003.3_TFAP2A	TFAP2A	1.989791343	0.196545801	2.392085088	0.007813675	0.039169852
MA0895.1_HMBOX1	HMBOX1	1.933883089	0.121422116	2.185021761	0.010595248	0.052432893
MA0695.1_ZBTB7C	ZBTB7C	1.928932198	0.29683293	2.145617331	0.010879671	0.0531589
MA0076.2_ELK4	ELK4	1.870393089	0.011499126	2.171390169	0.014794099	0.071381529
MA0668.1_NEUROD2	NEUROD2	1.85686287	0.174204495	2.03248577	0.015858666	0.075573394
MA0597.1_THAP1	THAP1	1.84650164	0.008324025	2.157062788	0.016718831	0.07870084
MA0748.1_YY2	YY2	1.827980998	0.297511302	2.230142585	0.018358654	0.0853788
MA0774.1_MEIS2	MEIS2	1.812721226	0.362708406	2.004580273	0.019813701	0.091048675
MA0766.1_GATA5	GATA5	1.810142076	0.385669407	2.075854747	0.020069312	0.091138286
MA0084.1_SRY	SRY	1.803765479	0.12347569	2.172882318	0.020713637	0.092970512
MA0497.1_MEF2C	MEF2C	1.794784031	0.361301167	2.017148882	0.021651662	0.096063694
MA0658.1_LHX6	LHX6	1.77722869	0.403617128	2.150190277	0.023592084	0.103483457
MA0756.1_ONECUT2	ONECUT2	1.760937045	0.502103631	2.094713485	0.025525664	0.110706811
MA0806.1_TBX4	TBX4	1.755941317	0.330060353	2.110062148	0.026145323	0.110902141
MA0807.1_TBX5	TBX5	1.755941317	0.330060353	2.110062148	0.026145323	0.110902141
MA0027.2_EN1	EN1	1.741246175	0.464534094	2.1105392	0.028043732	0.115895092

MA0068.2_PAX4	PAX4	1.741246175	0.464534094	2.1105392	0.028043732	0.115895092
MA0906.1_HOXC12	HOXC12	1.736729056	0.326890851	2.009906102	0.028650567	0.115895092
MA0689.1_TBX20	TBX20	1.736064737	0.192925097	1.850311767	0.028740756	0.115895092
MA0804.1_TBX19	TBX19	1.735455725	0.233456767	2.111825734	0.02882365	0.115895092
MA0732.1_EGR3	EGR3	1.727291159	0.647071259	2.026884084	0.02995489	0.119201935
MA0822.1_HES7	HES7	1.717914504	0.590417482	1.990377702	0.031300743	0.123286599
MA0048.2_NHLH1	NHLH1	1.711238589	0.115827386	2.092385179	0.032290129	0.125542579
MA0788.1_POU3F3	POU3F3	1.709686169	0.042883427	2.041015822	0.032523984	0.125542579
MA0692.1_TFEB	TFEB	1.705249171	0.175944542	2.087275564	0.033200343	0.126884481
MA0511.2_RUNX2	RUNX2	1.696988099	0.213137348	2.0494924	0.034491554	0.130526859
MA0812.1_TFAP2B(var.2)	TFAP2B(var.2)	1.684961715	0.226176201	2.059500541	0.036447385	0.136589228
MA0060.2_NFYA	NFYA	1.649193923	0.097770875	1.750281852	0.042827388	0.158928623
MA0828.1_SREBF2(var.2)	SREBF2(var.2)	1.647081173	0.029462343	2.014876616	0.043231879	0.158928623
MA0772.1_IRF7	IRF7	1.643413157	0.019436821	1.952725378	0.043941736	0.160014246
MA0051.1_IRF2	IRF2	1.640132216	0.334444066	1.980099537	0.044584928	0.160839087
MA0506.1_NRF1	NRF1	1.637350144	0.24186135	2.002997286	0.045136476	0.16132111
MA0833.1_ATF4	ATF4	1.634689091	0.509373641	1.911990181	0.045669358	0.161728185
MA0610.1_DMRT3	DMRT3	1.620206162	0.334618609	1.956880403	0.048662515	0.169437034
MA0495.1_MAFF	MAFF	1.619915954	0.068727281	1.982313813	0.048724121	0.169437034
MA0672.1_NKX2-3	NKX2-3	1.60161269	0.269971588	1.759181613	0.052742622	0.179080997
MA0031.1_FOXD1	FOXD1	1.594645583	0.566353267	1.856408915	0.054342595	0.179080997
MA0157.2_FOXO3	FOXO3	1.594645583	0.566353267	1.856408915	0.054342595	0.179080997
MA0042.2_FOXI1	FOXI1	1.588980695	0.454780438	1.90351689	0.055672849	0.179080997
MA0033.2_FOXL1	FOXL1	1.588980695	0.454780438	1.90351689	0.055672849	0.179080997
MA0847.1_FOXD2	FOXD2	1.588980695	0.454780438	1.90351689	0.055672849	0.179080997
MA0848.1_FOXO4	FOXO4	1.588980695	0.454780438	1.90351689	0.055672849	0.179080997
MA0849.1_FOXO6	FOXO6	1.588980695	0.454780438	1.90351689	0.055672849	0.179080997
MA0850.1_FOXP3	FOXP3	1.588980695	0.454780438	1.90351689	0.055672849	0.179080997
MA0652.1_IRF8	IRF8	1.585266643	0.158646163	1.927563001	0.056559467	0.179400995
MA0779.1_PAX1	PAX1	1.584674594	0.156866956	1.914433129	0.056701869	0.179400995
MA0867.1_SOX4	SOX4	1.58016685	0.360170269	1.913841077	0.05779578	0.180190891
MA0839.1_CREB3L1	CREB3L1	1.579801657	0.414398988	1.913719147	0.057885157	0.180190891
MA0755.1_CUX2	CUX2	1.574333704	0.087295292	1.985148309	0.059236991	0.182923827
MA0736.1_GLIS2	GLIS2	1.568490176	0.146798796	1.832406978	0.060710165	0.185985107
MA0080.4_SPI1	SPI1	1.560742037	0.166835898	1.747795933	0.06270956	0.190597561
MA0131.2_HINFP	HINFP	1.554149012	0.505735598	1.795999569	0.064452882	0.194365723
MA0037.2_GATA3	GATA3	1.546682853	0.193939804	1.731915708	0.066474478	0.198908128
MA0685.1_SP4	SP4	1.542500668	0.461357275	1.860181124	0.067629177	0.199685539
MA0687.1_SPIC	SPIC	1.541998448	0.256678475	1.884434917	0.067768926	0.199685539
MA0081.1_SPIB	SPIB	1.537111182	0.10762556	1.786674098	0.06914112	0.200771262
MA0782.1_PKNOX1	PKNOX1	1.536982105	0.093497113	1.692020758	0.069177663	0.200771262
MA0871.1_TFEC	TFEC	1.527765317	0.05374171	1.871470332	0.071827665	0.206906558
MA0802.1_TBR1	TBR1	1.520921595	0.066860213	1.641410649	0.073847802	0.210010382
MA0698.1_ZBTB18	ZBTB18	1.520434538	0.290605573	1.85041155	0.073993295	0.210010382
MA0825.1_MNT	MNT	1.518140818	0.343534906	1.843315178	0.074681571	0.210416688
MA0472.2_EGR2	EGR2	1.511411165	0.083676571	1.596429988	0.076730626	0.214623344
MA0670.1_NFIA	NFIA	1.495672899	0.421070698	1.8040473	0.081698419	0.226874747
MA0891.1_GSC2	GSC2	1.490274314	0.143259326	1.774937116	0.083460254	0.230111842
MA0665.1_MSC	MSC	1.482591288	0.136741428	1.782192158	0.08601944	0.235485843
MA0842.1_NRL	NRL	1.478449022	0.334408504	1.78645706	0.087424741	0.237647535
MA0059.1_MAX::MYC	MAX::MYC	1.476610108	0.334036242	1.784143967	0.088054389	0.237685273
MA0638.1_CREB3	CREB3	1.466404945	0.070200418	1.795466774	0.09161388	0.245576094
MA0593.1_FOXP2	FOXP2	1.457137066	0.32490997	1.682050188	0.094943425	0.252745945
MA0498.2_MEIS1	MEIS1	1.451935201	0.143512403	1.572698057	0.096853249	0.256064069
MA0792.1_POU5F1B	POU5F1B	1.448150946	0.005890906	1.530187539	0.09826133	0.258019548
MA0659.1_MAFG	MAFG	1.445391307	0.214283282	1.56235391	0.09929817	0.258980362
MA0650.1_HOXA13	HOXA13	1.443486353	0.075983522	1.743316084	0.100018837	0.259109202
MA0817.1_BHLHE23	BHLHE23	1.438131622	0.358618414	1.673045392	0.102066352	0.262650746
MA0675.1_NKX6-2	NKX6-2	1.428138715	0.215087136	1.620786823	0.105973999	0.269334431
MA0046.2_HNF1A	HNF1A	1.427924139	0.015063927	1.661521938	0.106059154	0.269334431
MA0071.1_RORA	RORA	1.418749235	0.146385392	1.71586627	0.10974976	0.274319776
MA0754.1_CUX1	CUX1	1.418261119	0.026437449	1.979622908	0.109948829	0.274319776
MA0761.1_ETV1	ETV1	1.417757981	0.009155927	1.718254181	0.110154314	0.274319776
MA0056.1_MZF1	MZF1	1.415134398	0.156641495	1.618951195	0.111230574	0.275224368
MA0901.1_HOXB13	HOXB13	1.40475358	0.21677355	1.712946147	0.115568043	0.284036446
MA0781.1_PAX9	PAX9	1.402142629	0.038320065	1.663223928	0.116678984	0.284036446
MA0009.2_T	T	1.401392933	0.044554154	1.655271754	0.116999469	0.284036446
MA0501.1_MAF::NFE2	MAF::NFE2	1.391056626	0.209611516	1.683440707	0.121486468	0.293086104
MA0679.1_ONECUT1	ONECUT1	1.384489079	0.012956804	1.473359338	0.124404191	0.29826098
MA0803.1_TBX15	TBX15	1.380895817	0.376782005	1.656599035	0.126022687	0.300276278
MA0479.1_FOXH1	FOXH1	1.370199707	0.227468856	1.492948433	0.130933937	0.310064415
MA0801.1_MGA	MGA	1.367343758	0.169329549	1.661203098	0.132269103	0.311316304
MA0088.2_ZNF143	ZNF143	1.354711506	0.189644997	1.587693532	0.138296314	0.322449245
MA0751.1_ZIC4	ZIC4	1.353942062	0.444907989	1.60850093	0.138669883	0.322449245
MA0516.1_SP2	SP2	1.338955861	0.132716192	1.550002601	0.146094742	0.335301201
MA0758.1_E2F7	E2F7	1.337834998	0.268629176	1.629108108	0.146661522	0.335301201
MA0528.1_ZNF263	ZNF263	1.337555986	0.046220991	1.55518803	0.146802857	0.335301201
MA0018.2_CREB1	CREB1	1.332340602	0.143046056	1.542384849	0.149463046	0.339369033
MA0700.1_LHX2	LHX2	1.327756381	0.310569256	1.557454594	0.151830077	0.342727542
MA0715.1_PROP1	PROP1	1.323471574	0.159665092	1.442690802	0.154066945	0.345754889
MA0613.1_FOXG1	FOXG1	1.321034235	0.016704771	1.615886303	0.155349917	0.346618891
MA0793.1_POU6F2	POU6F2	1.313081326	0.108300865	1.465470659	0.159589684	0.353375214
MA0866.1_SOX21	SOX21	1.311932411	0.470805151	1.530867072	0.16020897	0.353375214
MA0760.1_ERF	ERF	1.308907553	0.037550817	1.559892033	0.161847643	0.354961307
MA0036.2_GATA2	GATA2	1.302172216	0.003912975	1.5919609	0.165539315	0.361006641
MA0148.3_FOXA1	FOXA1	1.29254364	0.062459496	1.335788492	0.170920028	0.369510099
MA0162.2_EGR1	EGR1	1.290584788	0.060032947	1.351098809	0.172029614	0.369510099
MA0680.1_PAX7	PAX7	1.290090486	0.426472255	1.496361468	0.172310409	0.369510099
MA0757.1_ONECUT3	ONECUT3	1.286301595	0.003789265	1.489059627	0.174473445	0.372081491
MA0743.1_SCRT1	SCRT1	1.28177308	0.122324122	1.540524462	0.177083609	0.375572928
MA0492.1_JUND(var.2)	JUND(var.2)	1.269432467	0.070137651	1.525216857	0.184334485	0.388814815
MA0690.1_TBX21	TBX21	1.252081406	0.008965163	1.533364703	0.19487209	0.406586696
MA0156.2_FEV	FEV	1.250847285	0.142477079	1.417954185	0.195636898	0.406586696
MA0691.1_TFAP4	TFAP4	1.249723071	0.04745198	1.486314476	0.196335367	0.406586696
MA0905.1_HOXC10	HOXC10	1.246294504	0.267451416	1.434006519	0.198475952	0.406586696
MA0058.3_MAX	MAX	1.244437262	0.428548582	1.43917627	0.199642064	0.406586696
MA0674.1_NKX6-1	NKX6-1	1.241988412	0.241002041	1.50351226	0.201186681	0.406586696
MA0722.1_VAX1	VAX1	1.241988412	0.241002041	1.50351226	0.201186681	0.406586696

MA0723.1_VAX2	VAX2	1.241988412	0.241002041	1.50351226	0.201186681	0.406586696
MA0696.1_ZIC1	ZIC1	1.238742754	0.08044622	1.320571406	0.203246252	0.407343252
MA0764.1_ETV4	ETV4	1.238075165	0.008897682	1.472294658	0.203671626	0.407343252
MA0831.1_TFE3	TFE3	1.235131918	0.084407879	1.507079114	0.205554116	0.408340853
MA0864.1_E2F2	E2F2	1.23399221	0.028566948	1.469652033	0.206286182	0.408340853
MA0095.2_YY1	YY1	1.229549864	0.299349577	1.68637414	0.209156222	0.411909703
MA0902.1_HOXB2	HOXB2	1.227533491	0.190482022	1.482131758	0.210467642	0.412388376
MA0697.1_ZIC3	ZIC3	1.2159091	0.174078755	1.399539303	0.218134054	0.425251236
MA0878.1_CDX1	CDX1	1.211619249	0.178267511	1.362249528	0.221008891	0.428690614
MA0101.1_REL	REL	1.20276465	0.176644892	1.364507632	0.227020472	0.438149511
MA0154.3_EBF1	EBF1	1.197398754	0.092002288	1.442207063	0.230714343	0.443063365
MA0888.1_EVX2	EVX2	1.191558003	0.299724228	1.434168938	0.234778623	0.448303136
MA0740.1_KLF14	KLF14	1.190149311	0.157410514	1.443929565	0.235765639	0.448303136
MA0152.1_NFATC2	NFATC2	1.183829453	0.289558477	1.42028211	0.240226077	0.454545419
MA0115.1_NR1H2::RXRA	NR1H2::RXRA	1.162034294	0.051631464	1.461477082	0.256012513	0.48205283
MA0464.2_BHLHE40	BHLHE40	1.155426603	0.034100809	1.382388213	0.260921287	0.488910762
MA0787.1_POU3F2	POU3F2	1.152847176	0.049163693	1.428942884	0.262852893	0.490150805
MA0821.1_HES5	HES5	1.150018153	0.201226125	1.394004276	0.264981305	0.491744153
MA0066.1_PPARG	PPARG	1.145489455	0.109278224	1.39816222	0.268409952	0.495723643
MA0900.1_HOXA2	HOXA2	1.142746418	0.128256009	1.396867648	0.270499508	0.497203857
MA0907.1_HOXC13	HOXC13	1.134740563	0.026191424	1.603139833	0.276653132	0.506104781
MA0636.1_BHLHE41	BHLHE41	1.126496049	0.164561659	1.371799205	0.283075277	0.515410509
MA0786.1_POU3F1	POU3F1	1.123871193	0.222265061	1.525618497	0.28513792	0.515410509
MA0819.1_CLOCK	CLOCK	1.123099813	0.125678026	1.37271535	0.285745723	0.515410509
MA0872.1_TFAP2A(var.3)	TFAP2A(var.3)	1.113307159	0.03330745	1.29685324	0.29352629	0.526982083
MA0496.1_MAFK	MAFK	1.11116548	0.226012683	1.342388443	0.295243768	0.527611549
MA0057.1_MZF1(var.2)	MZF1(var.2)	1.106710354	0.003645795	1.317598806	0.298834554	0.531567456
MA0474.2_ERG	ERG	1.085640502	0.368021701	1.28738189	0.316142701	0.557125636
MA0730.1_RARA(var.2)	RARA(var.2)	1.084980435	0.157970413	1.323693637	0.316693489	0.557125636
MA0747.1_SP8	SP8	1.08397585	0.118239063	1.306489649	0.317532746	0.557125636
MA0513.1_SMAD2::SMAD3::SMAD4	SMAD2::SMAD3::SMAD4	1.080902082	0.145381868	1.305071556	0.320108011	0.55910268
MA0744.1_SCRT2	SCRT2	1.052823153	0.160211266	1.285697054	0.344135502	0.589742363
MA0618.1_LBX1	LBX1	1.049751623	0.283337353	1.266599408	0.3468174	0.589742363
MA0701.1_LHX9	LHX9	1.049751623	0.283337353	1.266599408	0.3468174	0.589742363
MA0716.1_PRRX1	PRRX1	1.049751623	0.283337353	1.266599408	0.3468174	0.589742363
MA0717.1_RAX2	RAX2	1.049751623	0.283337353	1.266599408	0.3468174	0.589742363
MA0882.1_DLX6	DLX6	1.049751623	0.283337353	1.266599408	0.3468174	0.589742363
MA0759.1_ELK3	ELK3	1.043227288	0.239496727	1.269832119	0.352548069	0.596857696
MA0738.1_HIC2	HIC2	1.038625892	0.201113116	1.202156948	0.356617138	0.60111011
MA0899.1_HOXA10	HOXA10	1.033185332	0.194895025	1.22934953	0.361457097	0.606619301
MA0153.2_HNF1B	HNF1B	1.02775581	0.261147974	1.247014577	0.366317884	0.612115599
MA0098.3_ETS1	ETS1	1.025137483	0.322188431	1.225102616	0.368672738	0.61251628
MA0762.1_ETV2	ETV2	1.02290104	0.028323039	1.438099505	0.370689636	0.61251628
MA0791.1_POU4F3	POU4F3	1.022205239	0.242243347	1.240113494	0.37131816	0.61251628
MA0714.1_PITX3	PITX3	1.012218081	0.033363244	1.174339748	0.380392608	0.624815093
MA0768.1_LEF1	LEF1	1.004297493	0.002943958	1.226054571	0.387658234	0.632818451
MA0712.1_OTX2	OTX2	1.002845828	0.035848933	1.145719083	0.388996313	0.632818451
MA0093.2_USF1	USF1	1.001559761	0.058436429	1.412681549	0.390183397	0.632818451
MA0468.1_DUX4	DUX4	0.997145431	0.012747935	1.180454167	0.394269641	0.636724075
MA0488.1_JUN	JUN	0.995399638	0.287848423	1.182835099	0.395890616	0.636724075
MA0856.1_RXRG	RXRG	0.983950615	0.147734606	1.123667691	0.40658872	0.651216788
MA0598.2_EHF	EHF	0.976788398	0.057065511	1.057346552	0.413339105	0.655231913
MA0884.1_DUXA	DUXA	0.975143915	0.287784603	1.119494089	0.414895128	0.655231913
MA0745.1_SNAI2	SNAI2	0.974582139	0.111401365	1.100650715	0.415427199	0.655231913
MA0644.1_ESX1	ESX1	0.974098429	0.188805675	1.174789706	0.41588554	0.655231913
MA0845.1_FOXB1	FOXB1	0.971511344	0.018693652	1.160441384	0.418340205	0.656419996
MA0741.1_KLF16	KLF16	0.969095051	0.064523996	1.174594792	0.420637735	0.656489493
MA0865.1_E2F8	E2F8	0.967889283	0.093546072	1.231169884	0.421785995	0.656489493
MA0471.1_E2F6	E2F6	0.965980252	0.249478772	1.161398209	0.423606347	0.6566749
MA0526.1_USF2	USF2	0.963340968	0.00726698	0.997462186	0.426127763	0.657941265
MA0475.2_FLI1	FLI1	0.949219011	0.04225068	1.149522234	0.439709195	0.676206172
MA0855.1_RXRB	RXRB	0.945373624	0.000651333	1.109560062	0.443432666	0.679226226
MA0805.1_TBX1	TBX1	0.942593791	0.093507181	1.142933775	0.446130815	0.680658081
MA0868.1_SOX8	SOX8	0.929664524	0.045241911	1.120100719	0.458748211	0.695070954
MA0673.1_NKX2-8	NKX2-8	0.929225031	0.223561792	1.118097975	0.459178998	0.695070954
MA0784.1_POU1F1	POU1F1	0.927249784	0.043114271	1.135360853	0.461116592	0.695277362
MA0091.1_TAL1::TCF3	TAL1::TCF3	0.923456943	0.014017342	1.048502764	0.46484381	0.69817008
MA0507.1_POU2F2	POU2F2	0.920354566	0.12139576	1.190830838	0.467898884	0.700034764
MA0823.1_HEY1	HEY1	0.911810735	0.126625926	1.019166808	0.476340828	0.707426942
MA0628.1_POU6F1	POU6F1	0.909695999	0.156568702	1.107168852	0.478436517	0.707426942
MA0780.1_PAX3	PAX3	0.909236686	0.260588249	1.092802245	0.478892006	0.707426942
MA0484.1_HNF4G	HNF4G	0.90785729	0.018080638	1.106806828	0.480260579	0.707426942
MA0258.2_ESR2	ESR2	0.906102186	0.030483096	1.187568171	0.482003331	0.707426942
MA0103.2_ZEB1	ZEB1	0.897837271	0.050164995	1.09970095	0.49023064	0.716776617
MA0827.1_OLIG3	OLIG3	0.895208474	0.223252048	1.073253772	0.492854271	0.71789339
MA0889.1_GBX1	GBX1	0.891483863	0.175274089	1.087129211	0.496576873	0.720596515
MA0661.1_MEOX1	MEOX1	0.888915755	0.149853407	0.989934047	0.499147096	0.721613405
MA0163.1_PLAG1	PLAG1	0.884824776	0.183208843	1.049170442	0.503247094	0.724826038
MA0473.2_ELF1	ELF1	0.87666689	0.135225461	1.050646365	0.511442345	0.733891246
MA0699.1_LBX2	LBX2	0.873692998	0.000145789	1.039251502	0.51443582	0.73545269
MA0818.1_BHLHE22	BHLHE22	0.86270469	0.056784351	0.948013556	0.525520988	0.748127656
MA0122.2_NKX3-2	NKX3-2	0.861065098	0.163411792	1.194667972	0.527178038	0.748127656
MA0599.1_KLF5	KLF5	0.85715726	0.046036161	0.962538915	0.53113029	0.749849377
MA0752.1_ZNF410	ZNF410	0.856024627	0.053757543	1.107397208	0.532276501	0.749849377
MA0903.1_HOXB3	HOXB3	0.854033805	0.100699365	0.987887374	0.534291909	0.749951553
MA0684.1_RUNX3	RUNX3	0.83688892	0.250915824	0.953037298	0.551680309	0.771552895
MA0820.1_FIGLA	FIGLA	0.831677262	0.103809429	1.009479235	0.556974504	0.776144977
MA0663.1_MLX	MLX	0.828156997	0.158382759	1.001723081	0.560551982	0.778320378
MA0625.1_NFATC3	NFATC3	0.822278674	0.293794713	0.955743193	0.566527659	0.783798123
MA0640.1_ELF3	ELF3	0.815105583	0.130643927	0.989086434	0.57382107	0.789093687
MA0074.1_RXRA::VDR	RXRA::VDR	0.814493089	0.05186203	1.020254811	0.57444385	0.789093687
MA0707.1_MNX1	MNX1	0.811823117	0.178096405	0.974173429	0.577158586	0.790011398
MA0641.1_ELF4	ELF4	0.806454642	0.212577058	0.968594716	0.582616331	0.79466397
MA0719.1_RHOXF1	RHOXF1	0.802659527	0.073074346	0.905661543	0.586473538	0.7971084
MA0508.1_PRDM1	PRDM1	0.796966703	0.073595672	1.0135084	0.592257097	0.802144699
MA0667.1_MYF6	MYF6	0.79188256	0.096969683	1.114279247	0.597419008	0.806306773
MA0596.1_SREBF2	SREBF2	0.780139541	0.184595996	0.906775472	0.609325234	0.815948328
MA0892.1_GSX1	GSX1	0.779765951	0.075943489	0.886849674	0.609703559	0.815948328

MA0504.1_NR2C2	NR2C2	0.777374457	0.072132123	0.946353602	0.612124592	0.815948328
MA0790.1_POU4F1	POU4F1	0.776179486	0.012408944	0.919600131	0.613333807	0.815948328
MA0733.1_EGR4	EGR4	0.774401785	0.042731946	0.947335742	0.61513203	0.815948328
MA0694.1_ZBTB7B	ZBTB7B	0.768726475	0.015993776	0.910422802	0.620867135	0.818308276
MA0159.1_RARA::RXRA	RARA::RXRA	0.768445232	0.090045591	0.926732463	0.621151101	0.818308276
MA0469.2_E2F3	E2F3	0.756573693	0.018676644	0.927913222	0.633114142	0.831231493
MA0783.1_PKNOX2	PKNOX2	0.749585828	0.077677619	0.905480029	0.640131784	0.833485744
MA0637.1_CENPB	CENPB	0.749065044	0.007292903	0.878848542	0.640653992	0.833485744
MA0724.1_VENTX	VENTX	0.74841168	0.163485069	0.90804576	0.64130898	0.833485744
MA0648.1_GSC	GSC	0.743457982	0.019000901	0.910372523	0.646268915	0.837113426
MA0763.1_ETV3	ETV3	0.730508965	0.02611459	0.892574155	0.659178957	0.848370989
MA0073.1_RREB1	RREB1	0.730331094	0.205322491	0.871435048	0.659355691	0.848370989
MA0729.1_RARA	RARA	0.726810314	0.040216407	0.872782145	0.662850372	0.850034032
MA0683.1_POU4F2	POU4F2	0.718120758	0.069403524	0.910232085	0.671444875	0.858204377
MA0141.3_ESRRB	ESRRB	0.714872706	0.146456126	0.869678343	0.67464556	0.859449459
MA0750.1_ZBTB7A	ZBTB7A	0.7020429	0.181450231	0.834569173	0.687219864	0.87258838
MA0678.1_OLIG2	OLIG2	0.696374602	0.090189244	0.841235688	0.692738088	0.875380996
MA0523.1_TCF7L2	TCF7L2	0.695121368	0.115570526	0.847726217	0.693954883	0.875380996
MA0813.1_TFAP2B(var.3)	TFAP2B(var.3)	0.689489732	0.090186943	0.933469636	0.6994077	0.879385577
MA0734.1_GLI2	GLI2	0.682403594	0.219204599	0.811777261	0.706232471	0.885083552
MA0442.1_SOX10	SOX10	0.678407639	0.026948837	0.801773364	0.710062397	0.887003512
MA0826.1_OLIG1	OLIG1	0.670551216	0.00829829	0.739073336	0.717551356	0.893279608
MA0656.1_JDP2(var.2)	JDP2(var.2)	0.668270451	0.024188084	0.920770169	0.719714917	0.893279608
MA0894.1_HESX1	HESX1	0.663395896	0.034694902	0.798522643	0.72432256	0.895214882
MA0635.1_BARHL2	BARHL2	0.661708142	0.108575398	0.806413786	0.725912586	0.895214882
MA0909.1_HOXD13	HOXD13	0.658163161	0.028459142	0.793604824	0.729243204	0.896458206
MA0024.3_E2F1	E2F1	0.652132169	0.091759822	0.847738648	0.734880541	0.897854509
MA0890.1_GBX2	GBX2	0.651765209	0.187188732	0.760577329	0.735222348	0.897854509
MA0144.2_STAT3	STAT3	0.647291723	0.1298387	0.786132713	0.739377884	0.897854509
MA0028.2_ELK1	ELK1	0.644198015	0.14081105	0.762534193	0.742239292	0.897854509
MA0785.1_POU2F1	POU2F1	0.644069282	0.024748204	0.862976931	0.742358135	0.897854509
MA0767.1_GCM2	GCM2	0.641924671	0.11884625	0.784758442	0.744335344	0.897854509
MA0130.1_ZNF354C	ZNF354C	0.632245389	0.09598958	0.764866485	0.753195544	0.901887364
MA0654.1_ISX	ISX	0.627562341	0.011219988	0.721710894	0.757443875	0.901887364
MA0702.1_LMX1A	LMX1A	0.627562341	0.011219988	0.721710894	0.757443875	0.901887364
MA0794.1_PROX1	PROX1	0.626899145	0.06097941	0.753030194	0.75804343	0.901887364
MA0863.1_MTF1	MTF1	0.622654619	0.077148809	0.758521546	0.761868252	0.901887364
MA0647.1_GRHL1	GRHL1	0.622499394	0.076980525	0.801925114	0.762007718	0.901887364
MA0014.2_PAX5	PAX5	0.619203063	0.00726074	0.746967768	0.764962493	0.901887364
MA0030.1_FOXF2	FOXF2	0.615207206	0.055583457	0.75189343	0.768526409	0.901887364
MA0686.1_SPDEF	SPDEF	0.613506274	0.075195301	0.737996374	0.770037439	0.901887364
MA0069.1_Pax6	Pax6	0.612371382	0.026208331	0.690997504	0.771043601	0.901887364
MA0862.1_GMEB2	GMEB2	0.6071845	0.008142724	0.741814581	0.775621263	0.90450093
MA0657.1_KLF13	KLF13	0.58175491	0.138804386	0.690878801	0.797545927	0.925053148
MA0137.3_STAT1	STAT1	0.581171888	0.059081487	0.67673133	0.798038078	0.925053148
MA0746.1_SP3	SP3	0.577805444	0.042323725	0.701647533	0.800870274	0.925556664
MA0914.1_ISL2	ISL2	0.568866394	0.068082652	0.686286412	0.808310479	0.928923948
MA0824.1_ID4	ID4	0.568519274	0.046878506	0.801658336	0.808597012	0.928923948
MA0160.1_NR4A2	NR4A2	0.561423177	0.13817493	0.679715578	0.814414741	0.932831127
MA0112.3_ESR1	ESR1	0.555283881	0.021969498	0.66389346	0.819385956	0.935748458
MA0800.1_EOMES	EOMES	0.549199334	0.012425476	0.659137943	0.82425489	0.937250822
MA0749.1_ZBED1	ZBED1	0.547558724	0.026226629	0.68076032	0.825557719	0.937250822
MA0642.1_EN2	EN2	0.54138704	0.161388298	0.651438485	0.830420155	0.940006393
MA0522.2_TCF3	TCF3	0.531243468	0.053210723	0.648447671	0.838277326	0.946125871
MA0776.1_MYBL1	MYBL1	0.525106577	0.030790072	0.640219888	0.842948117	0.946483366
MA0649.1_HEY2	HEY2	0.516543983	0.091884252	0.617382682	0.849358665	0.946483366
MA0645.1_ETV6	ETV6	0.512059043	0.104499411	0.615768439	0.852666182	0.946483366
MA0876.1_BSX	BSX	0.51193087	0.040389086	0.626409911	0.852760193	0.946483366
MA0595.1_SREBF1	SREBF1	0.51115009	0.122554356	0.620056474	0.853332258	0.946483366
MA0835.1_BATF3	BATF3	0.50887446	0.093473106	0.613223389	0.854993513	0.946483366
MA0725.1_VSX1	VSX1	0.504432193	0.015891786	0.591855448	0.858210306	0.946483366
MA0726.1_VSX2	VSX2	0.504432193	0.015891786	0.591855448	0.858210306	0.946483366
MA0155.1_INSM1	INSM1	0.488276676	0.089193461	0.681382374	0.869613076	0.951503878
MA0132.2_PDX1	PDX1	0.487705479	0.061082318	0.589607674	0.870007624	0.951503878
MA0830.1_TCF4	TCF4	0.487487997	0.059988104	0.612224864	0.870157692	0.951503878
MA0688.1_TBX2	TBX2	0.477318228	0.056414487	0.577351772	0.877078334	0.956362251
MA0765.1_ETV5	ETV5	0.465585301	0.028011077	0.56956971	0.884824718	0.956851396
MA0753.1_ZNF740	ZNF740	0.460567069	0.044961683	0.649098755	0.888059023	0.956851396
MA0138.2_REST	REST	0.460428066	0.145143091	0.546763513	0.888147934	0.956851396
MA0666.1_MSX1	MSX1	0.450904814	0.033237244	0.483655085	0.894151863	0.956851396
MA0708.1_MSX2	MSX2	0.450904814	0.033237244	0.483655085	0.894151863	0.956851396
MA0711.1_OTX1	OTX1	0.450198523	0.064163569	0.51386085	0.894590246	0.956851396
MA0789.1_POU3F4	POU3F4	0.449732209	0.131267491	0.51159458	0.894879155	0.956851396
MA0136.2_ELF5	ELF5	0.442341504	0.066816586	0.4919424	0.899402322	0.959031205
MA0072.1_RORA(var.2)	RORA(var.2)	0.435049014	0.078565938	0.52896624	0.903761942	0.961025095
MA0630.1_SHOX	SHOX	0.421238597	0.01360869	0.485067172	0.911734281	0.961555826
MA0703.1_LMX1B	LMX1B	0.421238597	0.01360869	0.485067172	0.911734281	0.961555826
MA0721.1_UNCX	UNCX	0.421238597	0.01360869	0.485067172	0.911734281	0.961555826
MA0259.1_ARNT::HIF1A	ARNT::HIF1A	0.414181832	0.024142812	0.499859004	0.915663546	0.962788983
MA0713.1_PHOX2A	PHOX2A	0.405098653	0.019210584	0.487723997	0.920576553	0.962788983
MA0149.1_EWSR1-FLI1	EWSR1-FLI1	0.402569426	0.055017868	0.491957216	0.921915542	0.962788983
MA0815.1_TFAP2C(var.3)	TFAP2C(var.3)	0.39609292	0.046295206	0.477096873	0.925286443	0.962788983
MA0834.1_ATF7	ATF7	0.39592078	0.041617131	0.499441009	0.925374903	0.962788983
MA0664.1_MLXIPL	MLXIPL	0.385510826	0.002766103	0.502663788	0.930615049	0.965638196
MA0140.2_GATA1::TAL1	GATA1::TAL1	0.373657607	0.036552072	0.456558485	0.936319483	0.968952601
MA0646.1_GCM1	GCM1	0.362548483	0.003116284	0.44567452	0.941412469	0.971618217
MA0731.1_BCL6B	BCL6B	0.351501943	0.115308565	0.416554151	0.946234308	0.973990514
MA0145.3_TFCP2	TFCP2	0.33226494	0.09506277	0.397191232	0.954057669	0.976096302
MA0083.3_SRF	SRF	0.329807842	0.012838164	0.40231046	0.955004796	0.976096302
MA0634.1_ALX3	ALX3	0.327545885	0.00968438	0.392161615	0.955866326	0.976096302
MA0032.2_FOXC1	FOXC1	0.303697488	0.01714809	0.324889829	0.964349954	0.982161167
MA0669.1_NEUROG2	NEUROG2	0.292420333	0.000986749	0.348804455	0.967985514	0.983269496
MA0718.1_RAX	RAX	0.274470677	0.026620867	0.311346287	0.973286314	0.985063527
MA0893.1_GSX2	GSX2	0.262512875	0.030377904	0.337389072	0.976494126	0.985063527
MA0735.1_GLIS1	GLIS1	0.253144491	0.069691748	0.301919457	0.978831181	0.985063527
MA0846.1_FOXC2	FOXC2	0.248388331	0.082948605	0.29153925	0.979959571	0.985063527
MA0662.1_MIXL1	MIXL1	0.19616482	0.034625404	0.23710746	0.989922031	0.992493257
MA0875.1_BARX1	BARX1	0.09001576	0.016112251	0.106622441	0.998999329	0.998999329

Supplementary Table 3: Oligonucleotide and siRNA sequences

ATAC-seq

Name	Sequence
Ad1_noMX	AATGATACGGCGACCACCGAGATCTACACTCGTCGGCAGCGTCAGATGT*G
Ad2.1	CAAGCAGAAGACGGCATACGAGATTCGCCTTAGTCTCGTGGGCTCGGAGATG*T
Ad2.2	CAAGCAGAAGACGGCATACGAGATCTAGTACGGTCTCGTGGGCTCGGAGATG*T
Ad2.3	CAAGCAGAAGACGGCATACGAGATTTCTGCCTGTCTCGTGGGCTCGGAGATG*T
Ad2.4	CAAGCAGAAGACGGCATACGAGATGCTCAGGAGTCTCGTGGGCTCGGAGATG*T

ChIP-qPCR

Region	fw primer	bw primer
CDC20 -41	GGTTGCGACGGTTGGATTTT	CTTTAACACGCCTGGCTTACG
CDC20 -428	GGCTCTCCTTCCCCTTCTAG	TCCGAGAACCTGCAGAAGTT
CDC20 +120	GGTGGCCCTGATTTTGTGG	CTGGAGGAAAGGAGGCGAC
CDC20 +1827	TCCACCACCATGATGTTCGG	CACTGGCCAAATGTCGTCCA
CDC20 +4020	GAGTCTGACCATGAGCCCAG	CTCAGGGTCTCATCTGCTGC
CDC20 +4113	ATGGCGCTGTTTTGAGTTGG	ACTGAGGTGATGGGTTGGTC
CDC20 enhancer 1	CCTGCAGAGTGAGCCATGTG	GAGCTGGCTGTGTCCTTTGA
CDC20 enhancer 2	AAATACTCTTGGCCACGCAC	AGTCTCACCATGCTGCCTAG
CDC20 TES	ACCTTTGGCCCAGAAGCTAC	CAAAGGGTCTCGTGGCCTT
CDC6 promoter	CTGTGGCCATTCGGATTT	CCCCTGAACAAACTGCACA
DLG5 enhancer	TGAGTGCTCCTGAGGAAGTG	TCTACTTCCTTACCGCCTGC
GAPDHS (CR)	GGCAGCAAGAGTCACTCCA	TGTCTCTTGAAGCACACAGGTT
IRF6 enhancer	TCCTTGACCCTGATTGAGCC	TCCAGACATTGACGCAGGTA
KIF23 promoter	CCTAACGTCCCGCAGTCTT	GCCTCGTACTCACGCTGAC
MINK1 enhancer	TCACACATCCCTCTGGCATA	AGAAGGAAGGCGAAGAAACC
NCAPH promoter	CTGCCTCACACTCCTCAGTT	CAACGGTAACCACATCGCTT
RRM1 promoter	CAGTCTGTGAAGCCTACCCC	GCGCAACAATCCAAATCCCC
SYNPO enhancer	CACGAGTCCCGTGTACCTG	AGCCTGTGCTACACACAGCA
TOP2A promoter	GGTGCCTTTTGAAGCCTCTC	TCCACCTATGAACGGCTGAG

RT-qPCR primers

Gene	fw primer	bw primer
AMOTL2	AGGCTGCAGAGAGACAATGAG	CTCAGAGAGCCGCTGGATT
AURKA	GCAGATTTTGGGTGGTCAGT	TCCGACCTTCAATCATTTCA
CDC20	CTGTCTGAGTGCCGTGGAT	TCCTTGTAATGGGGAGACCA
DLG5	ACCAGAAGGAGATCGGTGAC	ATCTCGGATGACCCGTTGT
IRF6	TTTGTCTGGAAACATTCCTTAGC	CCCCAAAGCATAAGTAGATCTCAA
MINK1	AGAAGCGGGGTGAGAAAGA	CGTTCATGATGGAGCTTGG
ΔNp63	GAAAACAATGCCCAGACTCAA	TGCGCGTGGTCTGTGTTA
p53	AGGCCTTGGAACTCAAGGAT	CCCTTTTTGGACTTCAGGTG
p63	CCCCACCTCTGAACAAAATGA	GGGTTGATAAGCTGGCTCACA
SYNPO	AGGGAGGACCTAGCAGACG	GTCAGCTGGGCTGCAATC
TOP2A	TCTGGTCCTGAAGATGATGCT	TTAGTTAACCATTCCTTTCGATCA
TAZ	GTATCCCAGCCAAATCTCGT	TTCTGCTGGCTCAGGGTACT
YAP	GACATCTTCTGGTCAGAGATACTTCTT	GGGGCTGTGACGTTCATC

guide RNAs

Region	fw primer	bw primer
cdc20_Enhancer1_sgRNA_a	CACCGGATAGCAAACTGATTTCTGG	AAACCCAGAAATCAGTTTGCTATCC
cdc20_Enhancer1_sgRNA_b	CACCGGGCTGGTGCACGGATCTGCA	AAACTGCAGATCCGTGCACCAGCCC
cdc20_Enhancer2_sgRNA_c	CACCGTTACCCACATTATCAATGGT	AAACACCATTGATAATGTGGGTAAC
cdc20_Enhancer2_sgRNA_d	CACCGGGACGTGTAAGGGAGCTCTG	AAACCAGAGCTCCCTTACACGTCCC
cdc20_Enhancer2_sgRNA_e	CACCGAGTGAGTAATCCATGGCATG	AAACCATGCCATGGATTACTCACTC
sgRNA_control	CACCGAATCTCGCTTATATAACGAG	AAACCTCGTTATATAAGCGAGATTC

siRNAs

Gene	Name/ sequence	Reference
ctrl	Cat#4390843	Thermo Fisher Scientific
YAP	UCUCUGACCAGAAGAUGUC	Azzolin Cell 2014; 158:157–70.
TAZ	ACGUUGACUUAGGAACUUU	Azzolin Cell 2014; 158:157–70.

Supplementary Table 4: Antibodies used in the study

Mouse monoclonal anti-β-Actin	Santa Cruz Biotechnology	Cat# sc-47778; RRID: AB_626632
Rabbit polyclonal anti-LIN9	Bethyl	A300-BL2981
Anti-mouse HRP conjugated	GE Healthcare	Cat# NXA931; RRID: AB_772209
HRP Protein A	BD Biosciences	Cat# 610438; RRID: N/A
IgG from rabbit serum	Sigma	Cat# I5006; RRID: AB_1163659
IgG from mouse serum	Sigma	Cat#I5381;RRID:AB_1163670
Mouse monoclonal anti CDK7	Cell Signaling	Cat#2916; RRID:AB_2077142
Mouse monoclonal anti Pol II 8WG16	Santa Cruz Biotechnology	Cat#:sc-56767; RRID:AB_785522
Mouse monoclonal anti Pol II CTD4H8 (Ser5)	Santa Cruz Biotechnology	Cat# sc-47701; RRID:AB_677353
Mouse monoclonal anti-CDC20	Santa Cruz Biotechnology	Cat# sc-13162; RRID: AB_628089
Mouse monoclonal anti-p53 (DO-1)	Santa Cruz Biotechnology	Cat# sc-126; RRID:AB_628082
Mouse monoclonal anti-p63 (D2K8X)	Cell Signaling	Cat#: 13109; RRID:AB_2637091
Mouse monoclonal anti-YAP	Santa Cruz Biotechnology	Cat# sc-101199;RRID: AB_1131430
Rabbit polyclonal anti-YAP	Novus Biologicals	NB110-58358 ; RRID:AB_922796
Rabbit polyclonal anti acetyl Histone H4 (K5,8,12,16)	Merck	Cat#06-598 RRID:AB_310550
Rabbit monoclonal anti B-MYB (phospho T487	Abcam	Cat# ab76009, RRID:AB_1309969
Rabbit polyclonal anti CDK7	Bethyl	Cat# A300-405A; RRID:AB_2275973
Rabbit polyclonal anti Histone H3 (K27 acetyl)	Merck	Cat# 07-360; RRID: AB_310550
Rabbit polyclonal anti Phospho-Chk1 (Ser345)	Cell Signaling	Cat#2341 ;RRID:AB_330023
Rabbit polyclonal anti RNA Pol II- phospho ser2	Abcam	Cat#ab5095; RRID:AB_304749
Rabbit polyclonal anti RNA Pol II- phospho ser5	Abcam	Cat# ab5131; RRID:AB_449369
GAPDH	Cell Signaling	Cat# 2118, RRID:AB_561053
Histone H2B	Abcam	Cat# ab1790, RRID:AB_302612
